# A Mass Spectrometric-Derived Cell Surface Protein Atlas

**DOI:** 10.1371/journal.pone.0121314

**Published:** 2015-04-20

**Authors:** Damaris Bausch-Fluck, Andreas Hofmann, Thomas Bock, Andreas P. Frei, Ferdinando Cerciello, Andrea Jacobs, Hansjoerg Moest, Ulrich Omasits, Rebekah L. Gundry, Charles Yoon, Ralph Schiess, Alexander Schmidt, Paulina Mirkowska, Anetta Härtlová, Jennifer E. Van Eyk, Jean-Pierre Bourquin, Ruedi Aebersold, Kenneth R. Boheler, Peter Zandstra, Bernd Wollscheid

**Affiliations:** 1 Institute of Molecular Systems Biology, ETH Zurich, Zurich, Switzerland; 2 Laboratory of Molecular Oncology, University Hospital Zurich, Zurich, Switzerland; 3 Department of Biochemistry, Medical College of Wisconsin, Wisconsin, Milwaukee, United States of America; 4 Institute for Biomaterials & Biomedical Engineering, University of Toronto, Toronto, Canada; 5 Oncology Research Laboratory, University Children Hospital Zurich, Zurich, Switzerland; 6 Centre of Advanced Studies, Faculty of Military Health Sciences, University of Defense, Hradec Kralove, Czech Republic; 7 Department of Medicine, Biological Chemistry and Biomedical Engineering, Johns Hopkins University School of Medicine, Baltimore, Maryland, United States of America; 8 Center for Systems Physiology and Metabolic Diseases, Zurich, Switzerland; 9 Faculty of Science, University of Zurich, Zurich, Switzerland; 10 SCRMC, LKS Faculty of Medicine, Hong Kong University, Hong Kong, Hong Kong SAR; 11 Division of Cardiology, Johns Hopkins University School of Medicine, Baltimore, Maryland, United States of America; 12 Department of Health Sciences and Technology, BMPP, ETH Zurich, Zurich, Switzerland; Institute of Molecular and Cell Biology, UNITED STATES

## Abstract

Cell surface proteins are major targets of biomedical research due to their utility as cellular markers and their extracellular accessibility for pharmacological intervention. However, information about the cell surface protein repertoire (the surfaceome) of individual cells is only sparsely available. Here, we applied the Cell Surface Capture (CSC) technology to 41 human and 31 mouse cell types to generate a mass-spectrometry derived Cell Surface Protein Atlas (CSPA) providing cellular surfaceome snapshots at high resolution. The CSPA is presented in form of an easy-to-navigate interactive database, a downloadable data matrix and with tools for targeted surfaceome rediscovery (http://wlab.ethz.ch/cspa). The cellular surfaceome snapshots of different cell types, including cancer cells, resulted in a combined dataset of 1492 human and 1296 mouse cell surface glycoproteins, providing experimental evidence for their cell surface expression on different cell types, including 136 G-protein coupled receptors and 75 membrane receptor tyrosine-protein kinases. Integrated analysis of the CSPA reveals that the concerted biological function of individual cell types is mainly guided by quantitative rather than qualitative surfaceome differences. The CSPA will be useful for the evaluation of drug targets, for the improved classification of cell types and for a better understanding of the surfaceome and its concerted biological functions in complex signaling microenvironments.

## Introduction

According to traditional phenotypic classification systems, the human body contains approximately 210 functionally distinct cell types [1,2]. Although knowledge about molecular features of these cell types is gathered at ever increasing speed, detailed information about the expressed cell surface protein repertoire of individual cell types is sparse due to technological limitations [3,4]. However, such information is a prerequisite to understand concerted biological functions of cell types in complex signaling environments. The surfaceome represents the subgroup of proteins at the plasma membrane with exposed domains towards the extracellular space including for example G-protein coupled receptors, receptor tyrosine kinases and integrins. This subgroup of proteins are of particular interest for basic and applied research due to their unique signaling functions, enabling, limiting and orchestrating cellular communication and interactions [5]. It is predicted, that the qualitative and quantitative cellular surfaceomes are more variable than other protein groups within the cell [6].

Genomic and transcriptomic technologies can provide informative hints about proteins expressed, but ultimately protein abundance, location and protein isoforms, including posttranslational modifications, must be directly measured and quantified in the cell surface location in order to deduce actual signaling capacities and in turn, functional consequences [7,8]. Global mRNA and protein quantification studes were already valuable in this respect, but have shown that correlation between mRNA levels and protein abundance is specifically low in relation to cell surface proteins [6].

Antibodies against cell surface proteins provided initial information and enabled the construction of limited surfaceome maps. The Cluster of Differentiation (CD) antigen panels [9], consisting mainly of antibodies that recognize cell surface proteins, led to the initial definition and partial characterization of various cell types of the hematopoietic system. This concept of defining and using cell surface protein markers for cell sorting and enrichment is beneficial for many research areas, as in the stem cell community [10–12] and in oncology. New cell surface markers for cancer detection, histological diagnosis and prognosis, as well as therapeutic intervention has been one of the key focus areas for academic, as well as industrial research for the last three decades. These combined efforts led to the discovery of over a dozen therapeutic antibodies. Rituximab, targeting CD20 [13], and Herceptin [14], targeting the epidermal growth factor receptor 2, are two prime examples. Multiplexed detection of cell surface proteins with antibodies in the form of serial antibody detection, parallel antibody arrays, bead-based formats, and most recently and noticeably mass cytometry have emerged as powerful tools to study the concerted co-expression of cell surface proteins [15–18]. Information gathered from such antibody-based technologies have been made easily accessible in databases such as UniProt (www.uniprot.org) [19], neXtProt (www.nextprot.org), Human Proteinpedia [20], and the Human Protein Atlas [17], in the latter already with tissue-specific resolution. However, antibody-based exploration of cell surface proteins is hampered by the availability of suitable antibodies to probe specific proteins.

Technological advancements in mass spectrometry (MS)-based proteomic technologies have enabled, in principle, the broad measurement of proteomes of individual cell types and whole organisms [21–23]. However, cell surface proteins are often underrepresented in these studies due to their low abundance and biochemical properties, such as the hydrophobicity of their transmembrane domains. Several biochemical technologies for enriching and analyzing membrane proteins by MS have been developed that typically employ initial density centrifugation [24,25], affinity enrichment by lectins [26], chemical tagging reagents [27,28], metabolic labeling [29] or even in situ labeling [30,31]. A complementary approach for the enrichment of plasma membranes is the employment of colloidal silica-beads [32,33]. Several reviews cover the technical challenges of analyzing plasma membrane proteomes [3,34] or in particular cell surface proteins [35] and the benefit thereof for biomedical research [36].

We previously developed the chemoproteomic Cell Surface Capture (CSC) technology [37], which enables the unbiased and selective discovery-driven assessment of the surfaceome, through a chemical tagging approach on viable cells. The CSC technology utilizes the fact that most cell surface proteins are predicted to be glycosylated. With the affinity enrichment of solely N-glycosylated hydrophilic glycopeptides it circumvents the biochemical difficulty of handling proteins containing hydrophobic transmembrane. This approach results in qualitative and quantitative information of the cellular surfaceome, which proved to be valuable in the context of biomedical applications [38–43].

Here, we used the CSC technology to generate surfaceome snapshots of 78 different human and murine cellular species and to build a Cell Surface Protein Atlas (CSPA) presented in the form of an easy-to-navigate interactive database and downloadable data matrices (http://wlab.ethz.ch/cspa). The CSPA provides experimental evidence on the protein level that quantitative surfaceome differences prevail over qualitative differences. Furthermore, we provide an associated toolbox, which expands and enables the targeted rediscovery of the identified surfaceome to nearly 1500 human and 1300 mouse cell surface proteins.

## Results

### The Cell Surface Protein Atlas at cellular resolution

The application of the CSC technology across community-defined cell types enabled us to measure surfaceome snapshots and to build the first Cell Surface Protein Atlas (CSPA) with cellular resolution. The CSC technology is based on tagging oxidized extracellular exposed glycans with a bifunctional cross linker for subsequent affinity enrichment and MS-based identification of formerly N-glycosylated peptides and their corresponding proteins. The enriched and formerly N-glycosylated peptides can be identified in the MS since the CSC protocol leads to a modification of the mass of the asparagine (N). The used panel of 47 human and 31 mouse cellular species (S1 Table) consists of cells derived from the three primary germ layers, endoderm (3 human, 1 murine cell lines), mesoderm (34 human, 16 murine cell lines) and ectoderm (10 human, 5 murine cell lines), as well as embryonic and adult stem cells with various degrees of lineage commitment. We collected surfaceome snapshots from various cell lines broadly used in research (e.g. A431, HeLa, HEK-293, and Jurkat T cells) as well as cell types used in more specialized research areas (e.g. primary natural killer (NK) cells and induced pluripotent stem (iPS) cells). Furthermore, cancer cell lines, including those derived from renal carcinoma, adenocarcinoma, sarcoma, melanoma and glioblastoma were analyzed. Three tissues samples were also included in the analysis (brain tumor, lymphoma and spleenocytes). The complete list of analyzed cellular species, descriptions, and references can be found in S1 Table.

**Fig 1:**
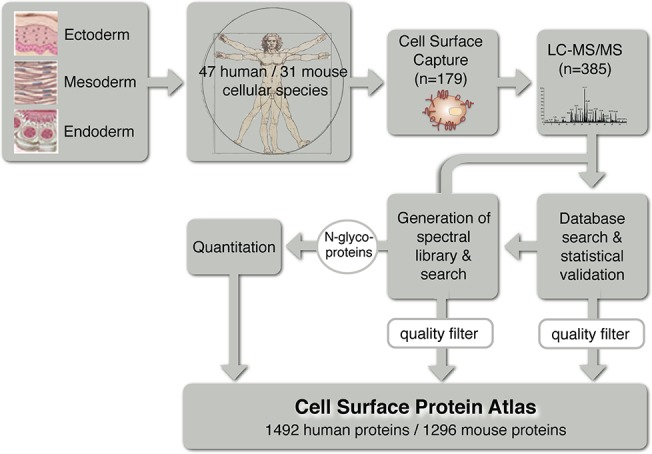
Workflow for building the CSPA with cellular resolution. Cell types of various origins were analyzed using the CSC technology. LC-MS/MS analyses and sequence database searches were performed. The resulting peptide-spectrum matches were used to build a spectral library, against which spectra from all the LC-MS/MS runs were matched. The identified N-glycoproteins were subjected to label-free relative quantification. The quality filtered protein list for N-glycoproteins from the sequence database and spectral library search was incorporated into the Cell Surface Protein Atlas, enriched with relative protein abundances.

The MS-based identification of cell surface exposed glycoproteins was done in two steps (Fig 1). First, probability scored peptide identifications were derived by using the classical protein database (UniProt) search algorithm SEQUEST, which matches the acquired peptide fragmentation pattern to theoretically derived spectra [44,45]. Second, spectra derived from identified peptides above a high confidence probability score (0.9) were used to build a spectral library [46]. This spectral library contains high quality annotated experimental spectra derived from cell surface glycopeptides. We used the SpectraST pattern matching and scoring algorithm to search the generated mass spectra against the high quality spectral library, which led to the assignment of additional peptide identifications of previously low scored peptide spectra, resulting in an average increase of protein identifications of 19% per cell type compared to classical protein identification strategies (Table D in S1 File). The CSPA spectral library is a new research tool, which enables the efficient scoring and rediscovery of cell surface glycopeptides and proteins by using the SpectraST peptide search engine.

**Fig 2:**
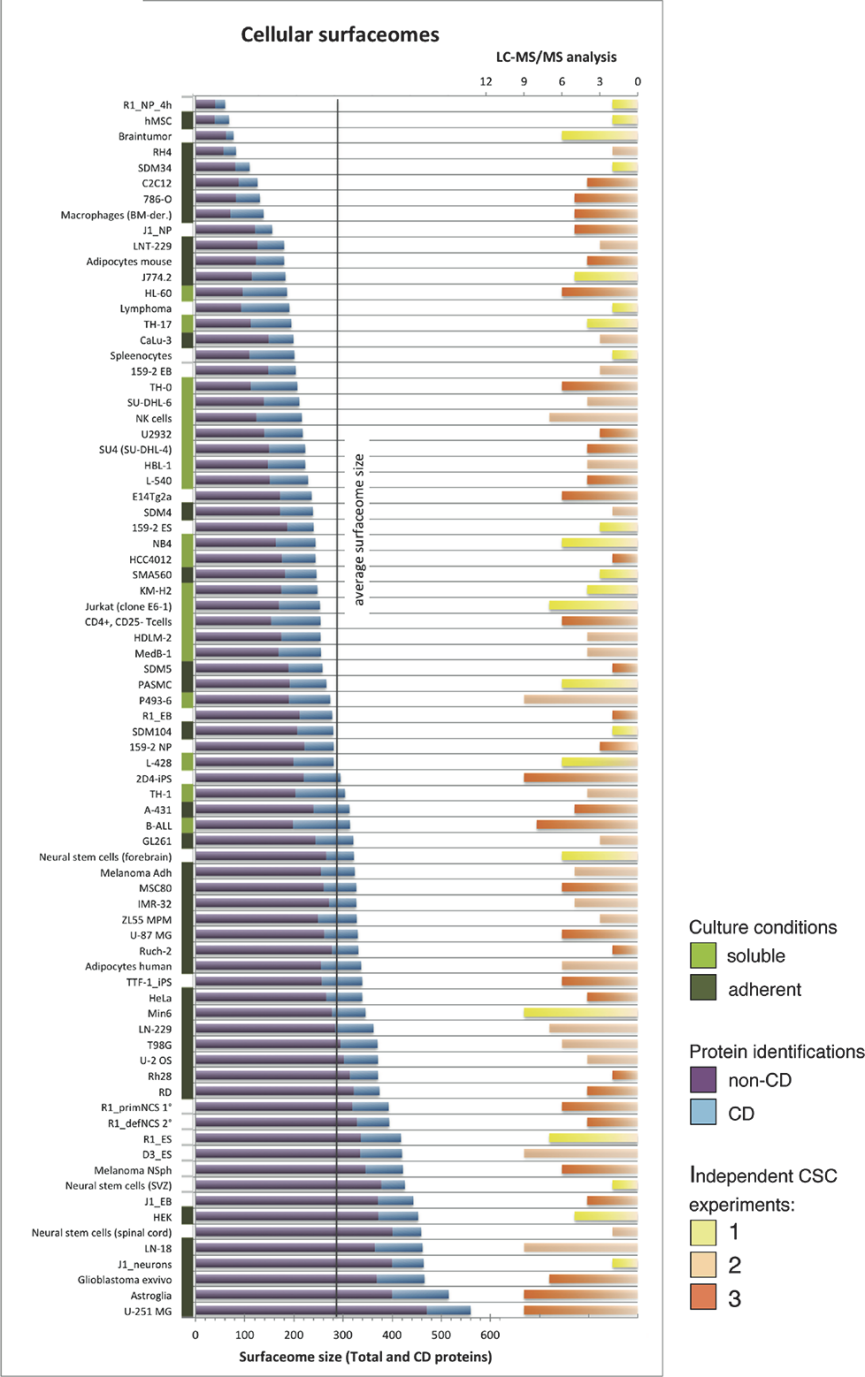
Cell-specific surfaceome sizes in relation to biological and technical replicates. The individual cell types investigated are listed with their surfaceome sizes (blue and purple bars from the left, blue = CD proteins, purple = other surface proteins). Adherent cells are labeled with dark green bars, soluble cells are labeled with light green bars, and cells with other growth properties (e.g. spheres) are not labeled. The bars from the right represent the number of LC-MS/MS runs performed. The color-code symbolizes the numbers of independent CSC experiments performed for that cell type (yellow = 1, orange = 2, red = 3).

The CSC technology and spectral library-based strategy revealed glycoproteins in the form of surfaceome snapshots with cellular resolution. On average, we detected 284 surfaceome-specific glycoproteins (277 in human and 294 in mouse samples) per CSC-analyzed cellular species (Fig 2 and Table D in S1 File). A subgroup of surfaceome members of special interest are the well-characterized CD antigens [9]. We detected on average 60 human and 90 mouse CD antigens per cell type. Notably, the majority of detected proteins were non-CD annotated proteins, surfaceome members invisible to most research strategies using affinity-based probes. Even though individual cellular species were profiled with different depth, the number of biological and technical replicates did not directly correlate with the detected surfaceome size (Fig 2), suggesting that either cellular species differ in their susceptibility for CSC analysis or vary substantially in the number and abundance of cell surface exposed proteins.

**Fig 3:**
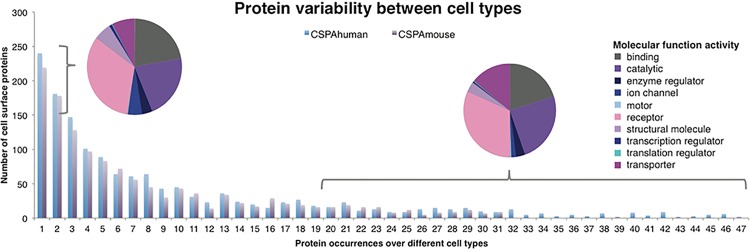
Distribution of protein occurrences over different cellular species. Proteins were classified into different bins (counts) based on the number of different cellular species on which they were detected (different observations). Since only 31 mouse cell types were investigated, the purple bar covers 1 to 31 observations. Human proteins with 1 or 2 observations and proteins with more than 20 observations are shown in two pie charts. The most prominent molecular functions found in both groups were binding, catalytic activity, receptor and transporter. Molecular functions were annotated by Gene Ontology.

The analysis of CSPA surfaceome snapshots further reveals a high degree of common proteins across all 47 human and 31 mouse cell types, with approximately 85% of the glycoproteins identified being present on more than one of the tested cell types (Fig 3 and Table B and Table C in S1 File). However, the remaining 15% (roughly 200 proteins) were not evenly distributed over the different cell types, thus not for every cell type there were specific proteins detected. Most of the proteins seen on one cell type were detected on one of those cell lines with the largest detected surfaceomes (like U-251 MG or HEK). This result provides further evidence that cell types usually cannot be inferred based on a single glycoprotein identification. Importantly, we did not observe major differences in functional annotation between proteins detected on a few cell types compared with proteins detected on more than 20 different cell types. As expected, most cell surface glycoproteins have functions associated with cell surface localization (e.g. receptors or transporters), indicating reliable protein identifications, even for proteins only detected on one cellular species (Fig 3).

**Fig 4:**
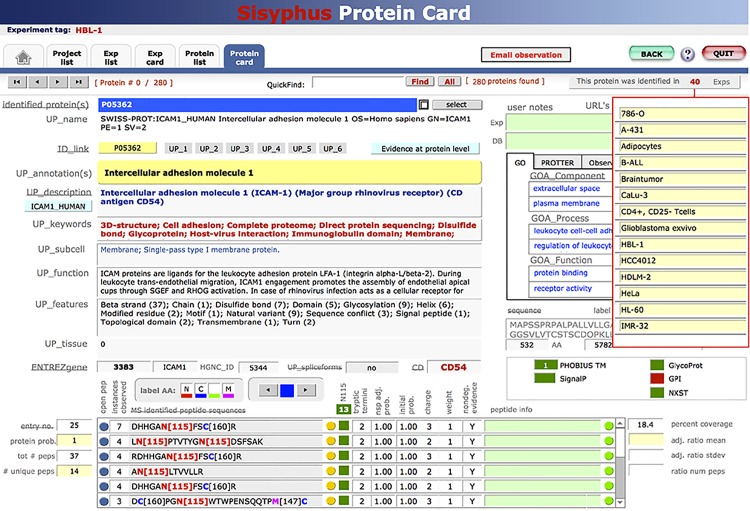
SISYPHUS screenshot of CD54 protein card. The protein card view is displayed for CD54 detected on HBL-1 cells. Annotations from various data sources (UniProt ID, UniProt Accession, ENTREZ gene, CD), UniProt keywords, subcellular locations, functions, molecular features, and tissue specificities are displayed (if known). The peptides identified from CD54 are listed on the bottom, together with the respective peptide probabilities, charge states, and further peptide-specific information. On the right, GO annotations are displayed, and by using the button on the top (“This protein was identified in 40 Exps”) a new window can be opened, displaying other cellular species on which CD54 was found.

The surfaceome snapshots at cellular resolution provided here are accessible in tab-delimited flat files (Table A, B and C in S1 File) and in an annotated form within the in-house developed and easy-to-navigate interactive Laboratory information management system (LIMS), termed SISYPHUS (http://wlab.ethz.ch/cspa). The SISYPHUS-CSPA enables the interrogation of the generated surfaceome snapshots in the context of MS and selected biological annotations (UniProt, Gene Ontology, STRING, PROTTER [47] and predictions (TMHMM, SignalP). The SISYPHUS-CSPA enables non-expert users to query the provided information apart from the downloadable list format on the cellular, protein, and peptide level (Fig 4). As an example, it is immediately visible whether a protein of interest has also been identified in any other surfaceome provided in the CSPA, enabling for example, informed selection of other cell types for antibody testing.

### Relative quantitative cell type specific surfaceome maps

**Fig 5:**
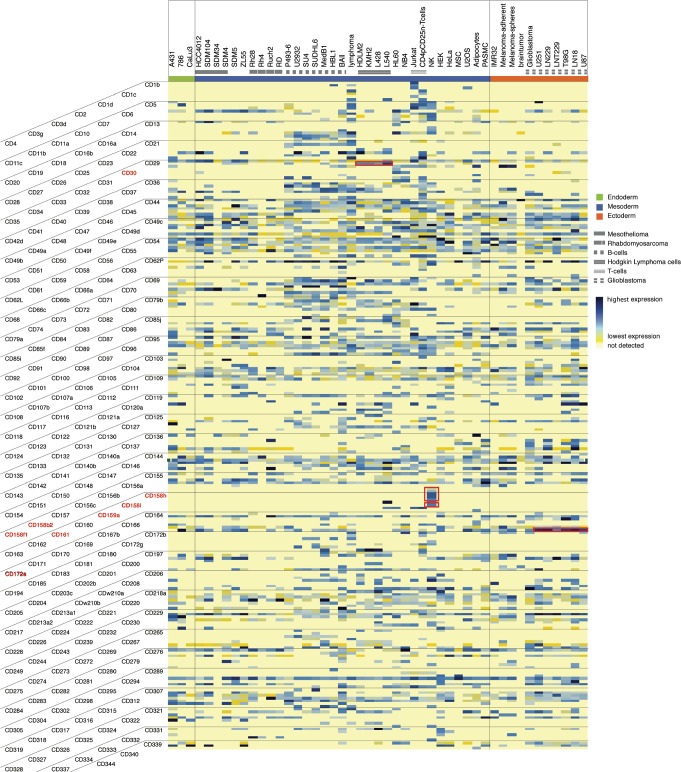
Expression matrix of human CD proteins. The 239 quantified CD proteins are listed according to their annotated number and their computed expression values in 47 human cell lines. Color code indicates expression level (blue = highest expression, yellow = lowest expression, light yellow = not detected). The cells are grouped according to their germ line origin (green = endoderm, blue = mesoderm, red = ectoderm) and functional groups. The most distinct cellular groups are annotated.

Qualitative protein panels containing multiple protein markers, i.e. CD panels, have been shown to be useful as cell-type classifiers [9]. Accordingly, quantitative protein abundance information on a broader range of cell surface proteins could aid in the refinement of cell surface protein panels for the discrimination of functionally different cellular species. Therefore, label-free quantification based on acquired peptide ion signals (peak area) from all detected formerly N-glycosylated peptides was used to determine abundance levels of 1438 human and 1259 murine proteins. The CSC-detectable surfaceome abundance range was up to 5 orders of magnitude (Table E and F in S1 File). The subset of 232 quantified human CD proteins is depicted in Fig 5, reflecting the abundance range of the whole dataset. CD63 and CD148 are highly abundant on certain cell types including the glioblastoma cell lines LN229 and T98G, and CD29 (even though detected on nearly every cell type) and CD142 are generally found at the lower end of the detected abundance range. None of the quantified CD proteins were highly expressed on all evaluated cell types. Protein-type specific quantitative variability across cell lines was in fact detected for essentially all proteins, suggesting that cell-type specific differences stem largely from quantitative differences within the set of membrane proteins, rather than from protein identities. Nevertheless, cell specific protein markers were also detected (Fig 5, highlighted), including immunoglobulin-like receptors CD158b2, CD158f1, CD158h, CD158i, CD159a and CD161 [48] only found on NK cells, as expected. CD30 is known as a characteristic Hodgkin lymphoma antigen [49], and even though we detected it also on other cell types, CD30 was expressed at the highest level on the Hodgkin lymphoma cell lines (HLDM2, KMH2, L428, L540). Similarly, CD172a (SIRPA) was found on the majority of cellular species but was most highly expressed on glioblastoma cells, reflecting published data that showed high expression of CD172a in brain [50]. CD172a was recently identified as a marker for cardiomyocytes derived from human pluripotent stem cells [51] and we compared CD172a expression amongst the mouse cell types within the CSPA (Table F in S1 File). CD172a was present on most murine cell types with expression levels rising from embryonic body (159–2_EB, R1_EB) to precursor cells (159–2_NP, R1_definitiveNCS) and with very high expression levels on induced pluripotent stem cells (2D4-iPS, TTF-1-iPS). The expression levels on embryonic stem cells reveal a diverse picture, as we did not detect CD172a on 159–2_ES cells, observed low expression levels on D3 cells, and found medium to high expression levels on E14Tg2a and on R1 cells. In summary, our analyses demonstrate that the CSPA is a valuable biological resource, which provides concerted protein expression information in the form of surfaceome maps of 78 human and mouse cell types. The quantitative expression matrix allows for both comparative analyses of detected cell surface proteins and categorization of cell surface proteins based on relative quantitative abundance levels.

### Defining the surfaceome

We combined the surfaceomes of the individual cell types to derive a composite surfaceome dataset, resulting in 1492 human and 1296 mouse experimentally verified proteins (Table A and B in S2 File). This experimentally derived and curated dataset was generated by filtering all the measured data according to the technical specifications of peptides observable by CSC. Since UniProt is currently the best curated proteomics database also providing subcellular localization information, we used these annotations for the comparative classification of our experimentally derived composite surfaceome. Proteins with UniProt keywords *Cell junction*, *Cell membrane*, *Secreted*, (*Signal peptide* AND *GPI-anchor)* or (*Signal peptide* AND *Transmembrane*) were regarded as high-confidence surfaceome proteins (1039 human and 881 mouse proteins, Table A and B in S2 File). 247 human and 242 mouse proteins from the remaining pool have predicted transmembrane domains (but none of the above keywords assigned) and are therefore regarded as putative surfaceome proteins. The functional classification of these proteins and the empirical data of this study provide a basis to refine the annotation of the surfaceome. A remaining set of 206 human and 173 mouse proteins were classified as unspecific. These unspecific proteins were co-purified mainly due to their high abundance in the biological samples. In contrast to affinity enriched N-glycosites, chemical deamidation of asparagines is known to occur in solution. Among these co-purified peptides are nevertheless proteins linked to extracellular matrix (as procollagen transferases) and cytoskeletal protein (as tubulin), which could very well be associated with or present at the cell surface. Overall, the composite surfaceome contains 240/197 CD proteins, 346/315 receptors; of which 69/67 are G-protein coupled receptors and 39/36 receptor tyrosine kinases, and 184/207 transporters (human/mouse).

### Topology prediction of cell surface proteins

Since the CSC technology is based on the enrichment of N-glycopeptides exposed to the extracellular space, the N-glycopeptides within the CSPA could deliver cues and restraints for confirmation of predicted or known protein topology, or in turn for protein topology correction. We compared the identified peptides of the CSPA proteins with predicted topology by using the PHOBIUS transmembrane prediction algorithm (http://www.ebi.ac.uk/Tools/pfa/phobius/, [52]) and found several experimentally observed N-glycopeptides located on predicted intracellular domains, thus conflicting with the empirical data. By using the identified N-glycosites as constraints for the topology, we were able to propose refined topology models for 51 human and 39 mouse proteins (S3 File). In the majority of cases, simply flipping the protein within the membrane layer yielded topologies in concordance with the CSC-identified N-glycopeptides. Furthermore, our data suggests that the topology prediction often fails for proteins with many (>10) TM domains (e.g. Q9HD45 in S3 File). This might be caused by the difficulty in defining the exact length of TM helices, which are known to range from 10 to 40 amino acids [53]. Very long TM helices could therefore be wrongly predicted as two short TM helices and vice versa, highlighting the need for experimental validation and the value of the CSPA and the CSC technology for experimental topology confirmation and possibly correction.

### The CSPA toolbox for surfaceome rediscovery

The surfaceome snapshots provided within the CSPA are of direct interest as a clinical resource for prioritizing cell surface-accessible biomarker candidates or targets for therapeutic antibody-drug conjugates. Therefore, the CSPA surfaceome snapshots provide the basis for simplified directed and targeted discovery and quantification of the previously detected surfaceome in other cells of interest. To facilitate fast MS-based detection and rediscovery of low abundant N-glycosylated cell surface proteins in future surfaceome experiments, we generated three different toolboxes. First, human and murine spectral libraries created with SpectraST (S4 and S5 Files) [46] are provided. Spectral library searching can be beneficial compared to classical sequence databases searching in terms of speed, number of identifications, and handling of noise [46]. The libraries contain decoy spectra in order to allow for estimation of the false discovery rate. We provide the spectral cell surface libraries in two flavors, one with those asparagines within N-glycosylation consensus motif (N-X-S/T) deamidated (Folder A in S4 File and in S5 File) and one completely unmodified version, where glycopeptides can be searched with variable modifications on asparagine residues (Folder B in S4 File and in S5 File).

A second toolset derived from our CSPA project includes MS coordinates for directed and targeted quantitative workflows [54]. In contrast to discovery-driven shotgun proteomic experiments, directed and targeted workflows allow for the instruction of the MS to selectively analyze only peptides pre-selected based on prior information. Based on the observed data within the CSPA, N-glycopeptides from proteins of interest can now be selected and specifically analyzed in any sample by using the instrument-specific inclusion list mode (Table A and B in S6 File).

CSPA also incorporates a third toolset, selected reaction monitoring (SRM) assays for the selective and multiplexed targeting of surfaceome members [55,56]. Based on the observed surfaceome-derived peptides, their fragmentation patterns, and consensus spectra, we extracted the most intense ions. This list of transitions provides an advanced starting point for the sensitive analysis and accurate quantification of the surfaceome in future experiments (Table C in S6 File). Together with spiked-in reference peptides, the CSPA observed N-glycopeptides could be analyzed in an absolute quantitative manner.

**Fig 6:**
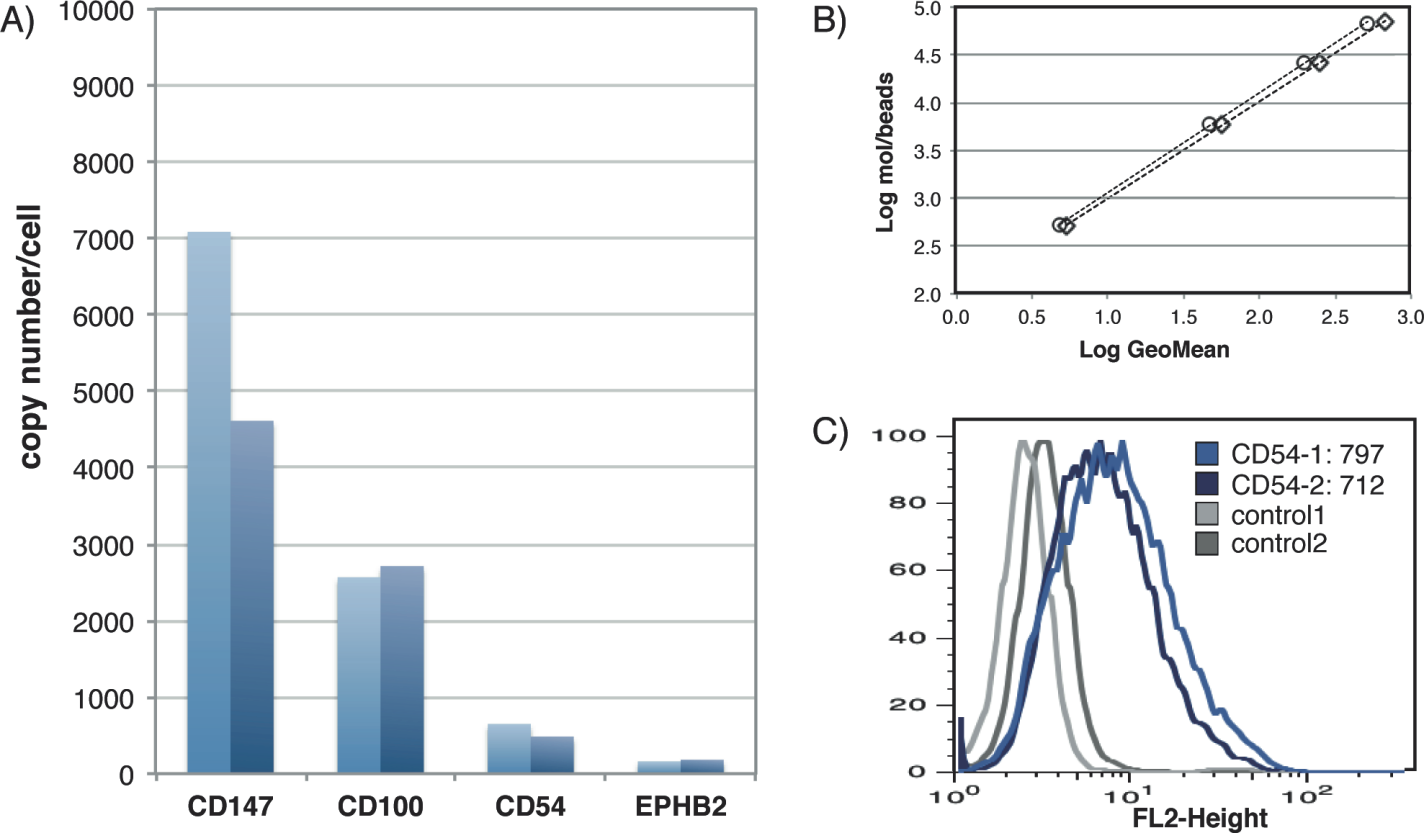
MS- and antibody-based absolute quantification of cell surface proteins. A) Calculated protein copy numbers per single cell from SRM measurements of CD147, CD100, CD54, and EPHB2. Two independent sample preparations and measurements were performed (light and dark blue bars). B) Calibration curve from QuantiBRITE beads. Slope and offset were calculated from the linear fit over the geometric mean of the four populations of beads with known fluorophore molecules bound. C) Flow cytometric analysis of unlabeled (grey) and CD54 labeled (blue) Jurkat cells in two replicates. Based on the calibration curve and the measured geometric mean, an average CD54 protein abundance per single cell was calculated.

To exemplify the applicability of such SRM workflows based on CSPA data, we chose four cell surface proteins, basigin (CD147), ephrin type-B receptor 2 (EPHB2), intercellular adhesion molecule 1 (CD54), and semaphorin-4D (CD100), and quantified their expression on Jurkat T cells by SRM. Except for CD147, where only one observed N-glycosite could be used, two previously observed N-glycosites per protein were chosen for quantification and measured with three transitions (MS1-MS2 mass/charge pairs) each (Table D in S6 File). To absolutely quantify the proteins of interest, we spiked the isotopically labeled analogs of previously observed peptides into the CSC samples in a known concentration. After MS analysis, this internal standard enabled the estimation of absolute protein copy numbers (Fig 6A). The yield for glycoprotein isolation and capturing through the CSC protocol was estimated by monitoring the abundance of a control glycoprotein (transferrin). The accuracy of these SRM measurements was assessed by quantitative flow cytometry (QuantiBRITE) for the cell surface expression of CD54. Unfortunately, there were no assays available at the time for the other proteins. The SRM measurements revealed approximately 550 CD54 molecules per cell (Fig 6A), which correlated well with the 750 molecules per cell obtained from the QuantiBRITE measurements (Fig 6B & 6C). This experiment demonstrated the applicability of the proposed strategy for systematic, targeted measurements of cell surface protein panels in order to obtain reliable quantification of selected cell surface proteins across many samples.

## Discussion

### CSPA as a resource for systems biology

Biologists in the “omics” fields rely on publicly available database information in order to turn the ever-increasing quantities of data into applicable knowledge. Although protein-centric databases, such as UniProt, contain valuable general information about proteins and their proteins, they do lack information about co-expression of protein pools in the context of cell types. Next generation databases such as neXtProt (http://www.nextprot.org) were created in response to the needs of the systems biology and biomedical communities for more detailed information and annotation required for cell-specific modeling approaches and biomarker qualification, respectively. This need for protein-centric resources with cellular resolution is further reflected by approaches to map out the total proteome of cells [21,22]. The CSPA now contains experimentally derived surfaceome snapshots at cellular resolution for a wide range of different human and murine cells. The CSPA data is presented in the interactive database SISYPHUS and online data matrix (http://wlab.ethz.ch/cspa/) that can be queried to provide answers to three main questions in a user-friendly and streamlined manner. First, was a particular protein found at the surface of a CSPA cell? Second, which are the co-expressed proteins or what is the detected surfaceome of a CSPA cell? Third, what are suitable peptides for targeted measurement of a selected CSPA protein? Since CD proteins are of interest for the large community of immunologists one could ask how many CD annotated proteins are co-existent at the cell surface? Typically, we identified an average of 280 proteins, including 70 CD proteins, per cell type. The highest number of CD annotated proteins detected together on one surfaceome dataset was 115 in case of the astroglial cells. This is interesting since CD-annotated proteins were originally described in the context and for the classification of mainly soluble cell types within the immune system. Our CSPA data indicates that CD markers could also prove valuable for the delineation of cell types in the brain. Furthermore, SISYPHUS-CSPA provides the cell-type-derived surfaceomes directly within the context of over 70 different cellular species and simultaneously provides publicly available biological protein annotations of, for example, gene ontology categories or known protein isoforms. The CSPA database provided here is unique, and complementary to the aforementioned resources in respect to the experimental focus on solely cell surface exposed proteins and not the total membranome or proteome, irrespective of specific protein localization. Therefore, the CSPA contains valuable information about concerted protein expression and abundances in the surfaceome location, which is critical for understanding complex cellular communication at the molecular level.

### Tools for the rediscovery of cell surface proteins

The CSPA expands our detection ability to nearly 1500 human and 1300 mouse cell surface proteins. Up until now, a lack of suitable tools for detection, in particular antibodies, most surfaceome members identified by our study could not easily be detected otherwise. Additionally, the lack of applicable parallel detection technologies has led to the characterization of only a small subset of proteins on the cell surface. About 370 antibodies against CD-annotated proteins have been used for flow cytometry-based on DotScan [15] testing. These antibodies could theoretically be used in batches up to twelve (flow cytometry) or sixty antibodies (DotScan) in parallel to scan cell lines in a serial fashion. However, CD annotated proteins account for less than 20% of the composite surfaceome and the specificity of most CD antibodies is actually not known. To alleviate this issue, we also provide a toolbox including spectral libraries and transition lists, enabling the selective and targeted detection of cell surface proteins. The surfaceome targeting workflows are based on state-of-the-art MS technologies, such as AIMS, SRM, and SWATH technology, which allows for reproducible and/or absolute quantitative assessment of cell surface protein expression. Limited independent validation of absolute quantitative cell surface protein estimations indicated the usefulness of the MS assay protocols provided as part of CSPA (Fig 6). The MS coordinates allow for systematic profiling of the surfaceome: For example, one could evaluate tumor model cell lines to establish pharmaceutically relevant tumor fingerprints. Thus, the CSPA not only provides detailed information for the ‘omics’ community, but its associated tools also enable the future quantitative interrogation of the surfaceome data space for biological and pharmaceutical applications.

### Towards the characterization of the complete surfaceome

Although the CSPA represents the most extensive experimentally-derived surfaceome database so far, the provided surfaceome maps are not complete. The issue of incompleteness is also faced by other discovery-driven and non-targeted databases such as PHOSIDA [57], PhosphoPep [58], and PhosphoSitePlus [59]. The reasons for this incompleteness are manyfold and are mainly due to a lack of sensitivity of currently available MS instrumentation, limitations of our workflow (like N-glycosylation) in combination with biological peculiarities (like other unanticipated modifications). The non-detection of a particular peptide does not rule out the absence of a particular glycoprotein and similar to other strategies, its absence needs to be independently verified. Nevertheless, comparisons of the CSPA in its current form to other datasets in the public domain is informative. Comparisons with the quantified proteome of the human cell line U2-OS [23] reveals that the proteins within the CSPA on the same cells span the entire abundance range with a bias towards lower abundance proteins. Specifically, the proteins classified in our analysis as “high confidence” are predominantly located in the lower abundance range of the Beck et al. dataset, whereas contaminant proteins are clearly of higher abundance. Also the limited SRM analysis of the four cell surface proteins presented here, supports this finding. This implies three conclusions: First, contaminant proteins are present in the CSPA mainly due to their high abundance. Second, specifically enriched glycoproteins from the cell surface are generally lower abundant. Third, the proteins that are informative of a cell type tend to be present in lower abundance.

### Quantification of cell surface proteins increases discriminatory power between cellular species

Significant research efforts have been made to define specific cell surface markers for various applications and resarch areas, for examples for stem cell at various differentation stages, for markers allowing for better sorting and enrichment of new functional cellular subsets in cell biology and for targets for antibody drug conjugates in pharmaceutical research. However, finding an appropriate marker from a long list of identified cell surface proteins is a daunting task. The depth and breadth of the CSPA overcomes this limitation, by providing comparative information about the “uniqueness” of potential candidates. As an example of this utility, we recently demonstrated how the list of 502 putative positive selection markers for human pluripotent stem cells (hPSC) could be narrowed down to 206 restricted glycoproteins after comparison with the CSPA [60]. Furthermore, the CSPA enabled the proposition of 15 negative selection markers for hPSC. Although qualitative surfaceome comparisons can be informative, the integrated CSPA reveals that obtaining quantitative surfaceome maps are the crucial next step in surfaceome research. Our relative abundance estimates of cell surface proteins confirm current knowledge about several cell type-specific markers and provide a rich source for user-specific inquiries. Comparison of cell surface protein expression levels can be made between different cellular species and allows ranking of proteins by their estimated relative abundance. Notably, the quantified data within the CSPA suggests that surfaceome differences emerge largely on a quantitative level within cell surface protein expression rather than on a qualitative level (protein identities). Since biomarker candidate lists are often highly enriched for cell surface proteins, the CSPA quantitative matrix could aid in prioritizing candidates based on their abundance level for follow-up screens and could therefore help to reduce cost and time in biomarker development. The CSPA toolbox then allows for specific and sensitive measurement of selected prequalified cell surface proteins.

## Material and Methods

### Cell culture

Cell lines were cultured according to guidelines from the American Type Culture Collection (ATCC) or from published cell culture conditions. The cell types were annotated and classified through information provided by ATCC or the academic laboratory that provided the cells. ATCC catalog numbers and literature references for each cell type are listed in S1 Table. Generally, soluble cells were grown to a density of 0.1 to 1 million cells per ml and adherent cells were passaged at 80% confluence. For each CSC experiment, either 1 x 10^8^ soluble cells were harvested, or 5 x 15 cm dishes with adherent cells at 80% confluence. Tissues were harvested with minimal disturbance of cellular integrity and dissociated mechanically or when necessary with protease mixtures (collagenase, dispase); 200 mg to 1 g tissue was used per CSC experiment.

### Tissues and animal cells

Human and mouse adipocytes were kindly provided by Prof. Christian Wolfrum (Schwerzenbach, Switzerland) and the use thereof approved as stated in [61]. Astroglial cells were kindly provided by Prof. Dr. Burkhardt Becher (Zurich, Switzerland) and the use thereof approved as stated in [62]. B-ALL cells were kindly provided by Prof. Dr. med. Jean-Pierre Bourquin (Zurich, Switzerland) and the use thereof was approved as stated in [42]. The brain tumor sample and the primary glioblastoma cells, which were expanded ex vivo, were kindly provided by Prof. Dr. med. Karl Frei (Zurich, Switzerland) and the use thereof was in accordance with the Declaration of Helsinki and approved by the ethics committee of the Canton Zurich.

The lymphoma sample were kindly provided by PD Dr. med. Marianne Tinguely (Zurich, Switzerland) in accordance with the Helsinki declaration and Swiss laws and was approved by the official authorities of the ethical committee of the Canton Zurich (StV2-2007). The melanoma cells and the neural stem cells from the forebrain were kindly provided by Prof. Dr. Lukas Sommer (Zurich, Switzerland) and the use thereof was in accordance with the Swiss federal and cantonal laws on animal protection and approved by the ethics committee of canton Zurich. Neural stem cells of the spinal cord and the subventricular zone was kindly provided by Dr. Michaela Thallmair (Zurich, Switzerland) and the use thereof approved as stated in [63]. The study of natural killer cells was approved as stated in [64]. The study of spleenocytes was approved as stated in [37]. The T-cell subsets TH-0, TH-1 and TH-17 were kindly provided by Prof. Dr. Manfred Kopf (Zurich, Switzerland) and the use thereof was in accordance with Swiss federal legislation and has been approved by the local overseeing body Gesundheitsdirektion Kanton Zürich, Veterinaeramt (permission 148/2005).

### Cell Surface Capture

For each CSC experiment, either 1x10^8^ suspension cells, 5 x 15cm dished with adherent cells at 80% confluence or 200 mg to 1 g of tissue were used. CSC was performed as described previously [37]. In detail, cells were treated for 15 min at 4°C in the dark with 2 mM sodium meta-periodate (Pierce) in PBS, pH 6.5 and were then incubated with 6.5 mM biocytin hydrazide (Biotium) in PBS, pH 6.5 for 60 min. Homogenization was done in hypotonic lysis buffer (10 mM Tris, pH 7.5, 0.5 mM MgCl_2_, and 10 mM iodoacetamide) using a Dounce homogenizer. Cell debris and nuclei were removed by centrifugation at 1,700 *g* for 10 min and the supernatant was centrifuged again in an ultracentrifuge at ~150,000 *g* for 1 h. The solubilized membrane pellet was reduced (5 mM TCEP, 30 min), alkylated (10 mM iodoacetamide, 30 min) and digested overnight with trypsin. Trypsin was inactivated for 10min at 95°C and biotinylated glycopeptides were bound to Streptavidin Plus UltraLink Resin (SA beads; Pierce). After extensive washing, N-linked glycopeptides were enzymatically released from the SA beads overnight by PNGase F (New England Biolabs). Peptides were desalted on Ultra MicroTIP Columns (The Nest Group) according to the manufacturer’s instructions and dried in a SpeedVac concentrator. Finally, peptides were solubilized in LC-MS grade water containing 0.1% formic acid and 5% acetonitrile.

For the following cell lines: B-ALL, HBL-1, HDLM-2, HL-60, KM-H2, L428, L540, MedB-1, NB4, SU4, SUDHL-6, U2932 (all human) and TH0, TH1, TH17 (all mouse) a slightly adapted CSC protocol was applied in which proteins were not reduced and alkylated before digestion. Instead, after enriching for N-linked glycopeptides, peptides bound via di-sulfide bridges to N-linked glycopeptides were released by reduction in a first elution step. N-linked glycopeptides were thereafter released by PNGase F. For the CSPA, only results from the N-glycopeptide fraction were included.

### Reverse-phase chromatography and mass spectrometry

Peptide samples were analyzed either on a Tempo Nano 1D+ HPLC system (Applied Biosystems/MDS Sciex) connected to a 7 tesla Finnigan LTQ-FT-ICR instrument (Thermo Scientific) or on an Eksigent Nano LC System (Eksigent Technologies) connected to a hybrid LTQ Orbitrap XL (Thermo Scientific). Both systems were equipped with a nanoelectrospray ion source (Thermo Scientific). In total, 385 LC-MS/MS runs were performed. In the following, generic methods for the LTQ-FT and LTQ Orbitrap XL are described. Some samples were analyzed with slightly different settings.

On the LTQ-FT-ICR system, peptides were separated on a RP-HPLC column (75 μm x 15 cm) packed in-house with C18 resin (Magic C18 AQ 3 μm, 200 Å; Michrom BioResources) using a linear gradient from 96% solvent A (0.15% formic acid) and 4% solvent B (98% acetonitrile, 2% water, 0.15% formic acid) to 35% solvent B over 60 or 90 minutes at a flow rate of 0.3 μl/min. Each MS1 scan (acquired in the ICR cell) was followed by collision-induced dissociation (CID, acquired in the LTQ) of the five most abundant precursor ions with dynamic exclusion for 30 seconds. Only MS1 signals exceeding 150 counts were allowed to trigger MS2 scans with wideband activation disabled. Total cycle time was approximately 1 to 1.5 s. For MS1 scans, 3x10^6^ ions were accumulated in the ICR cell over a maximum time of 500 ms and scanned at a resolution of 100,000 FWHM (at 400 m/z). MS2 spectra were acquired using the normal scan mode, a target setting of 10^4^ ions, and an accumulation time of 100 ms. The normalized collision energy was set to 32%, and one microscan was acquired for each spectrum.

On the LTQ Orbitrap XL system, chromatographic separation of peptides was carried out on a RP-HPLC column (75 μm x 10.5 cm) packed in-house with C18 resin (Magic C18 AQ 3 μm, 200 Å; Michrom BioResources) using a linear gradient from 95% solvent A (0.15% formic acid) and 5% solvent B to 35% solvent B (98% acetonitrile, 2% water, 0.1% formic acid) over 60 min at a flow rate of 0.3 μl/min. The data acquisition mode was set to acquire one high-resolution MS scan in the Orbitrap followed by five CID MS/MS scans in the linear ion trap. One microscan was acquired per MS/MS scan. For a high-resolution MS scan, 2 x 10^6^ ions were accumulated over a maximum time of 400 ms and the FWHW resolution was set to 60,000 (at m/z 300). Only MS signals exceeding 250 ion counts triggered a MS/MS attempt, followed by dynamic exclusion for 30 seconds, and 10^4^ ions were acquired for a MS/MS scan over a maximum time of 200 ms. The normalized collision energy was set to 35%. Singly charged ions and ions with unassigned charge states were excluded from triggering MS/MS scans in both systems.

### Database searching

Proteins were identified by searching MS and MS/MS data of peptides with the SEQUEST search engine [44] against the UniProt/SwissProt Protein Knowledgebase (version 57.15 of either *Homo sapiens* or *Mus musculus* taxonomy) concatenated to the reversed sequences of all proteins and common contaminants (40521 entries human, 32455 entries mouse), with a precursor mass tolerance of 0.2 Dalton. Other search parameters were at least one tryptic terminus, two maximal internal cleavage sites, carbamidomethylation of cysteines as fixed modification (add 57.021464 Da), deamidation of asparagines (add 0.984016 Da) and oxidation of methionines (add 15.9949 Da) as variable modifications. Probability scoring was performed by PeptideProphet and ProteinProphet within the Trans-Proteomic Pipeline TPP v4.3.1 [45]. The ProteinProphet probability score was set individually for each cell type to a false discovery rate (FDR) of 1%.

The MS-based proteomics data have been deposited to the ProteomeXchange Consortium (http://proteomecentral.proteomexchange.org) via the PRIDE partner repository [65] with the dataset identifier PXD000589.

### Spectral library generation and searching

Spectral libraries were built and searched with SpectraST 4.0. Peptides with a higher peptide probability than 0.9 were extracted and used to create consensus spectra. Consensus spectra were filtered with the SpectraST quality filter level 2. All asparagines in the motif N-X-[ST] (wherein N stands for asparagine) were set as deamidated and all other asparagines were set as unmodified. An equal number of decoy spectra were appended to the spectral libraries. The original mzXML files were searched against the created spectral libraries with SpectraST with carbamidomethylation as a fixed modification. Probability scoring was performed by PeptideProphet and ProteinProphet using the non-parametric model based on decoy-estimated FDR [45]. The probability cutoff was set individually for each cell type to reach an estimated FDR of 1%.

### CSPA assembly

For all cell types in the CSPA, at least two technical replicates (replicate LC-MS/MS analyses of the sample sample) were acquired. All samples in the CSPA were required to display specificity for N-glycopeptides of over 50%. The average specificity for N-glycopeptides was over 75%. A maximum of three independent CSC experiments per cell type and three LC-MS/MS runs per experiment were integrated in the CSPA. The identified proteins were filtered for the presence of at least one peptide with a deamidated asparagine (N[115]), measured with at least two independent scan events. We also included peptides with deamidated asparagines outside the N-glycosylation motif for two reasons: First, depending on the fragment ions identified, SEQUEST has problems assigning the modification to the correct amino acid when more than one asparagine is present in the peptide sequence. Second, N-linked glycosylation outside the consensus motif has recently been shown to occur in rare cases [66]. Search results from the classical database search and the spectral search were combined to create a non-redundant surfaceome list; members were classified based on UniProt predicted subcellular locations.

### SISYPHUS-CSPA

SISYPHUS is a Filemaker based MAC/PC compatible database, developed in-house (http://wlab.ethz.ch/cspa/). It processes output files from the trans-proteomic pipeline, assigns biologically relevant context information derived from various online databases (e.g., UniProt, Gene Ontology), and presents the data in a user-friendly, browsable format. We provide SISYPHUS populated with the datasets from the spectral searches.

### Label-free quantification and further processing

Peptide precursor intensities were extracted by the label-free option of the XPressPeptideParser, which is an integral part of the TPP [67]. Resulting raw intensities were logarithmized and quantile normalized per LC-MS/MS run. Proteins were quantified by MSstats [68], which applies fixed ANOVA models for each individual protein. No imputation was performed. For proteins only detected in one cell type, the average abundance of all detected features was calculated.

Transmembrane domains were predicted by Phobius, version 1.01 [52]. Gene ontology enrichment was obtained from the PANTHER webserver [69]. Further processing and visualization of the data was performed in R, TIBCO Spotfire Professional 3.1.0 (TIBCO Software Inc.), Protter [47] or by Perl scripts and an in-house database software.

### Selected reaction monitoring

CSC samples from Jurkat cells were produced as described above, except that 100 μg of biotinylated transferrin was spiked into the solubilized microsomal pellet. Holo-transferrin (200 μg, 98% purity, Sigma Aldrich) was biotinylated in a 20-min oxidation with 10 mM sodium meta-periodate (Pierce) in PBS, pH 6.5, cleaned over a C18 column (Sep-Pack Vac C18 cartridge 100 mg, Waters), followed by a 1.5 hour incubation with 500 μg biocytin hydrazide (Biotium) and cleaned again with a C18 column. Transferrin was monitored by SRM together with four cell surface proteins (CD147, CD100, CD54, EPHB2) in order to determine protein loss during the procedure. Table D in S6 File contains the measured peptides with the respective transitions. SRM measurements were done on a 6460 Triple Quadrupole instrument (Agilent Technologies) equipped with an HPLC-Chip cube and connected to an Agilent 1200 series nano-LC system. Peptide samples were loaded first to the 160-nl C18 enrichment column embedded in the HPLC chip (large capacity chip, 150 mm 300 Å C18 with 160 nl trap column, Agilent Technologies) and subsequently separated chromatographically over a 60 min gradient from 97% solvent A (0.2% formic acid) to 35% solvent B (97% acetonitrile, 0.2% formic acid) with a flow rate of 0.3 μl/min. Collision energy was calculated by the formula (([precursor mass/charge] * 3.6)/100–4.8), and fragmenter voltage was set to 130 V. The transitions were measured with a dwell time of 20 ms and a MS1 and MS2 resolution of 0.7 FWHW. Further processing of the raw SRM data was performed in Skyline v0.6 [70].

### Flow cytometry

CD54 levels on Jurkat cells were analyzed by direct immunofluorescence. Briefly, 1 x 10^6^ cells were washed with PBS, 0.1% fetal bovine serum (FBS) and then incubated for 30 min at 4°C with anti-CD54-PE (BD Biosciences) in a 1:50 dilution. Cells were washed again with PBS, 0.1% FBS and analyzed on a FACSCalibur System (BD Biosciences). Ten thousand events were measured per analysis and subsequently exported to FlowJo7 (Tree Star). QuantiBRITE reference beads (BD Biosciences) were resuspended in 500 μl PBS, 0.1% FBS and measured on a FACSCalibur System (BD Biosciences).

## Supporting Information

S1 Table Annotation of cell types. Description and origin of all cell types and tissues used for the CSPA.
(XLS)

S1 File Matrix of all proteins and their detection in the different cell types. Excel file containing 6 tables organized in different sheets. A. List of all proteins identified within the different cell types. B. Matrix of 1492 human proteins against 47 human cell types. C. Matrix of 1296 human proteins against 31 human cell types. D. Table containing the number of identified proteins of each cell type. E. Matrix with human surfaceome proteins and cells and their estimated relative quantities in log2 scale. F. Matrix with mouse surfaceome proteins and cells and their estimated relative quantities in log2 scale.
(XLSX)

S2 File CSPA validated surfaceome proteins. Excel file containing all human and mouse surfaceome proteins in two tables and an additional table with all identified N-glycopeptides. A. List of 1492 human surfaceome proteins and their annotation. B. List of 1296 mouse surfaceome proteins and their annotation. C. List of 13942 mouse and human derived N-glycopeptides, including identified modified form.
(XLSX)

S3 File Corrected topologies. PDF files with original and based on N-glycopeptide identification corrected topology pictures of 51 human proteins and 39 mouse proteins. The pictures were created with PROTTER and identified N-glycopeptides were marked yellow.
(PDF)

S4 File CSPA based spectral libraries for human proteins. ZIP file, containing a README.txt file and two subfolders with the respective spectral libraries. A. The .pepidx, .spidx and .splib file of the human spectral library for proteins within the CSPA. The sequence motif N-X-S/T has been modified to D-X-S/T, which corresponds to a deamidated asparagine (N). Methionines are variable modified by oxidation and a decoy spectral library is appended. B. The .pepidx, .spidx and .splib file of the human spectral library for proteins within the CSPA. Asparagines and methionines can be searched with variable modifications of deamidation and oxidation, respectively and a decoy spectral library is appended.
(ZIP)

S5 File CSPA based spectral libraries for mouse proteins. ZIP file, containing a README.txt file and two subfolders with the respective spectral libraries. A. The .pepidx, .spidx and .splib file of the mouse spectral library for proteins within the CSPA. The sequence motif N-X-S/T has been modified to D-X-S/T, which corresponds to a deamidated asparagine (N). Methionines are variable modified by oxidation and a decoy spectral library is appended. B. The .pepidx, .spidx and .splib file of the mouse spectral library for proteins within the CSPA. Asparagines and methionines can be searched with variable modifications of deamidation and oxidation, respectively and a decoy spectral library is appended.
(ZIP)

S6 File CSPA toolbox. Excel file containing tables for generating inclusion lists and transition list of surfaceome proteins within the CSPA. A. Human inclusion list. B. Mouse inclusion list. C. Transition list. D. Measured transitions of Fig 6.
(XLSX)
