# Supplementary material for: A Mass Spectrometric-Derived Cell Surface Protein Atlas
Source: PLoS One. 2015 Apr 20;10(4):e0121314. doi: 10.1371/journal.pone.0121314 (PMC4404347; doi:10.1371/journal.pone.0121314)

corrected topology of human proteins

A7MBM2

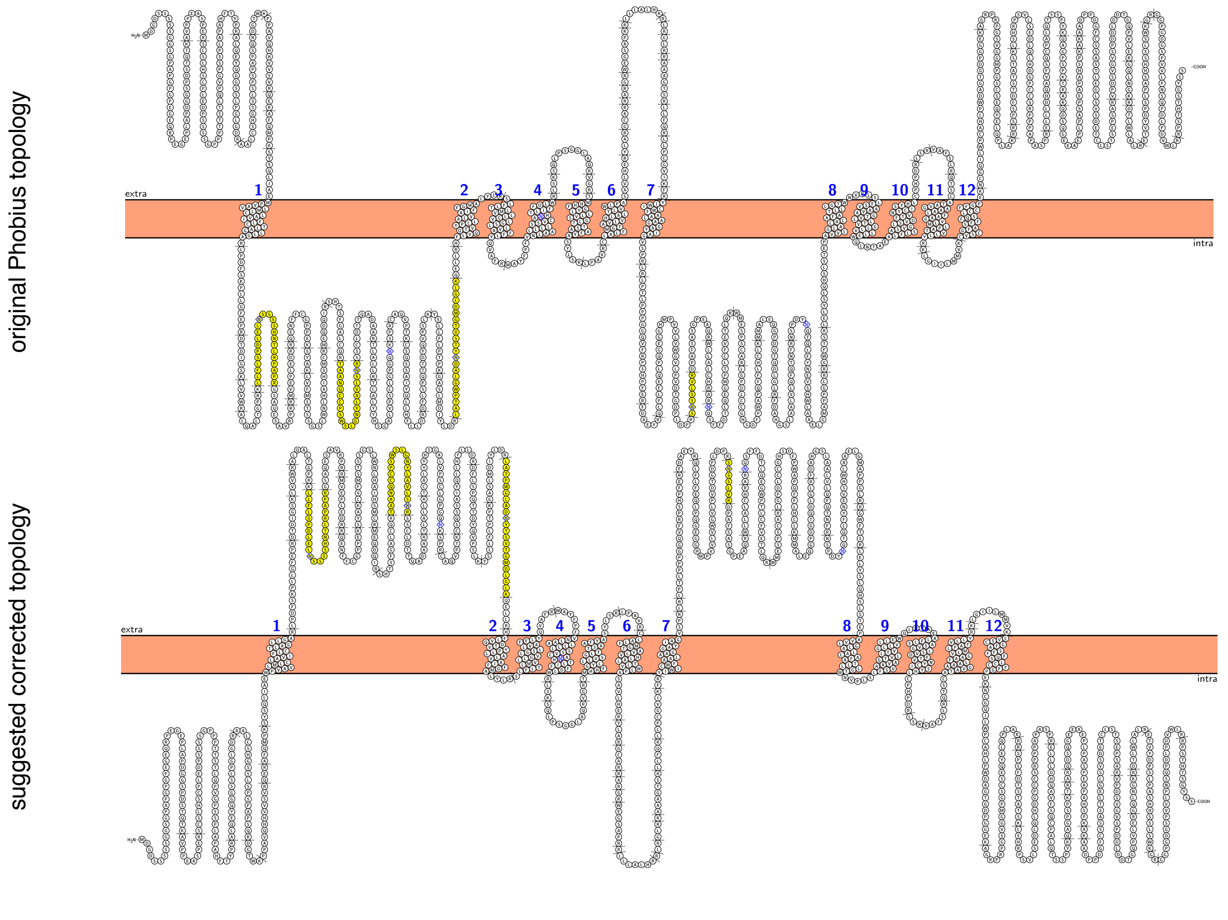

O14524

original Phobius topology

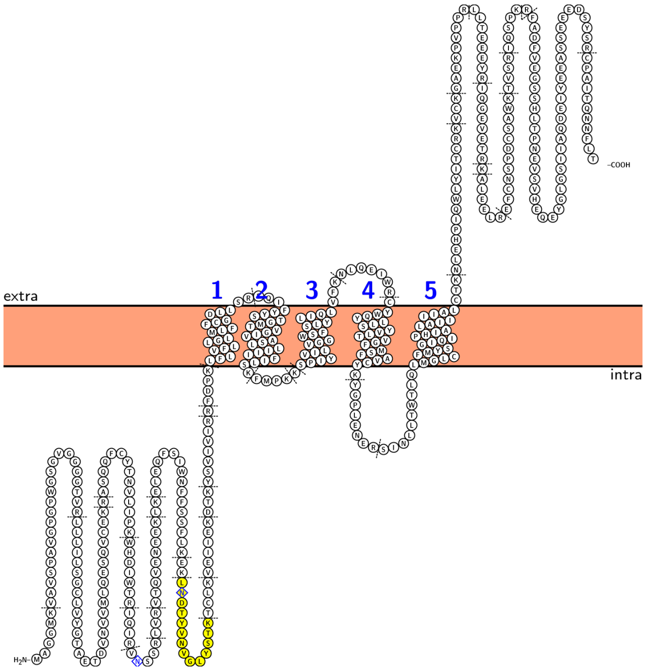

suggested corrected topology

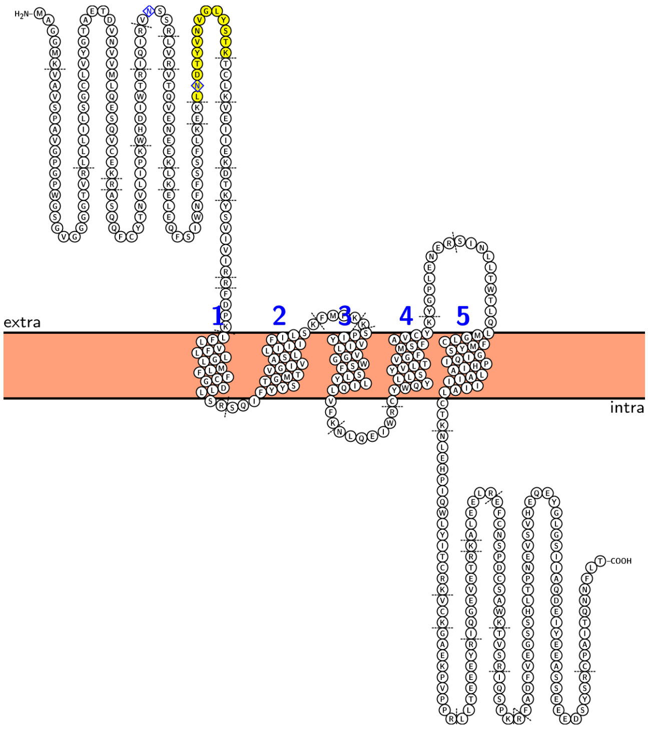

O75396

original Phobius topology

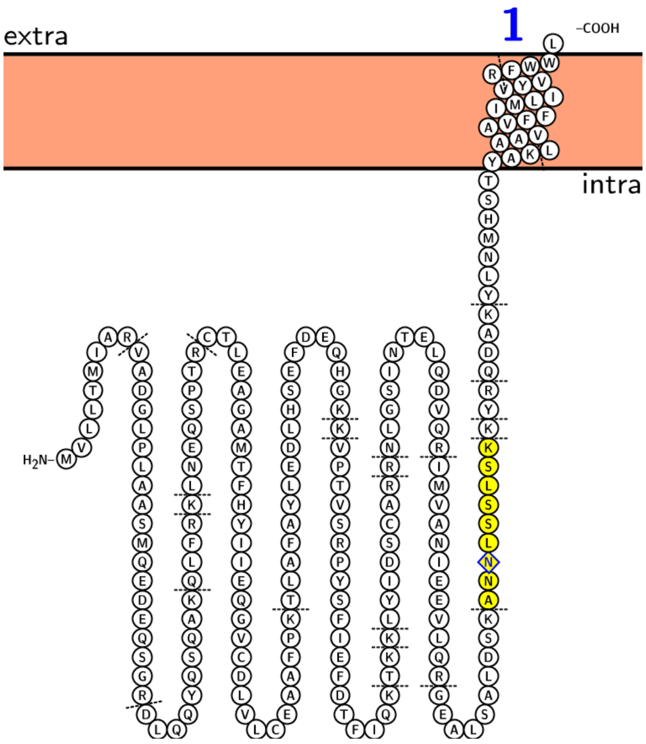

suggested corrected topology

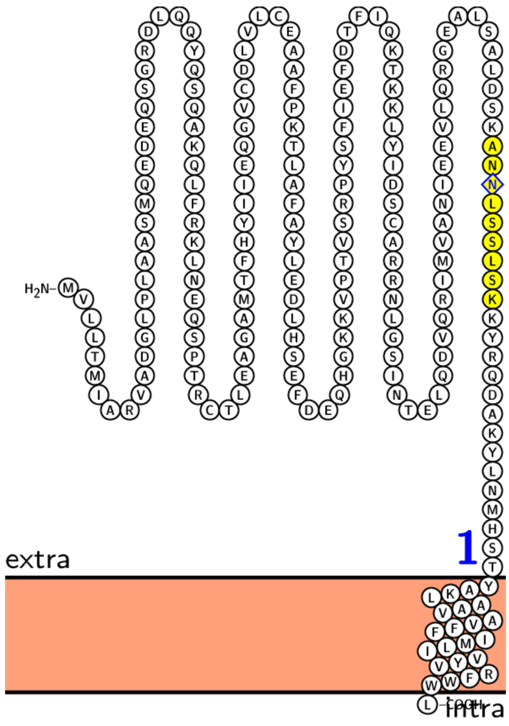

O94933

original Phobius topology

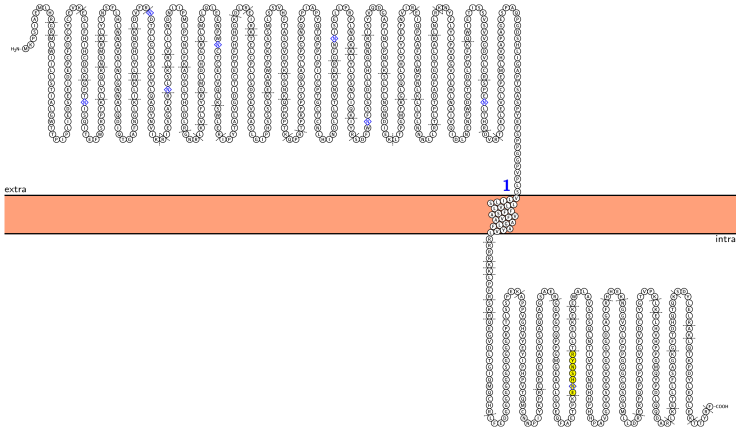

suggested corrected topology

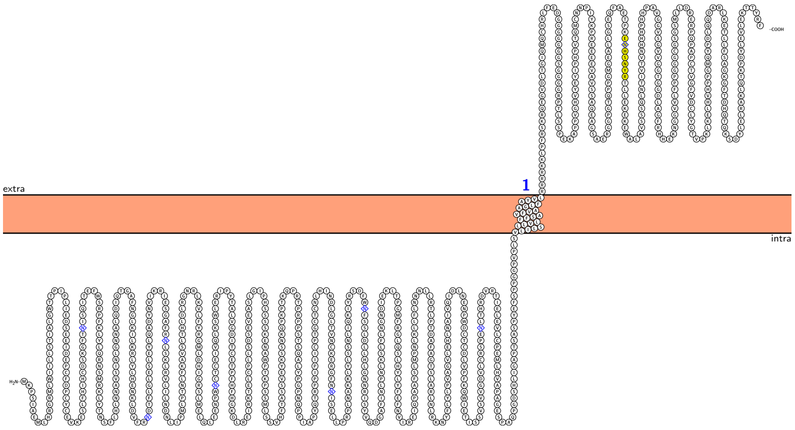

O95183

original Phobius topology

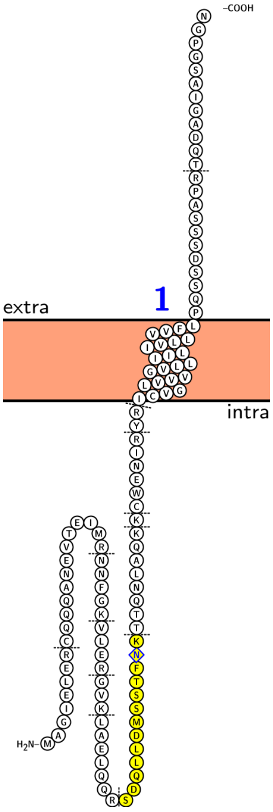

suggested corrected topology

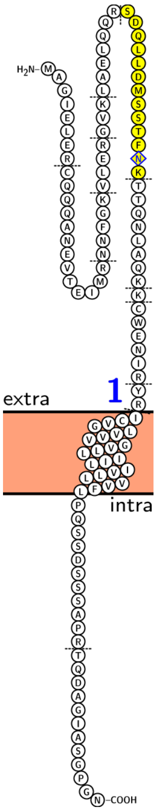

P00533

original Phobius topology

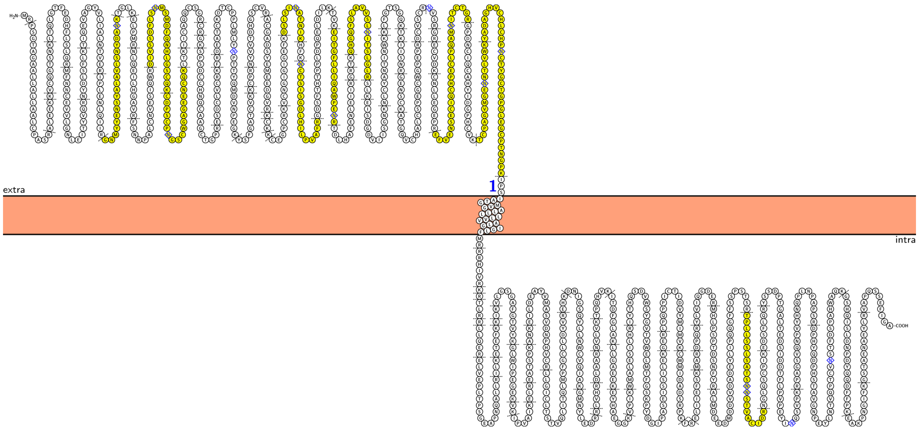

suggested corrected topology

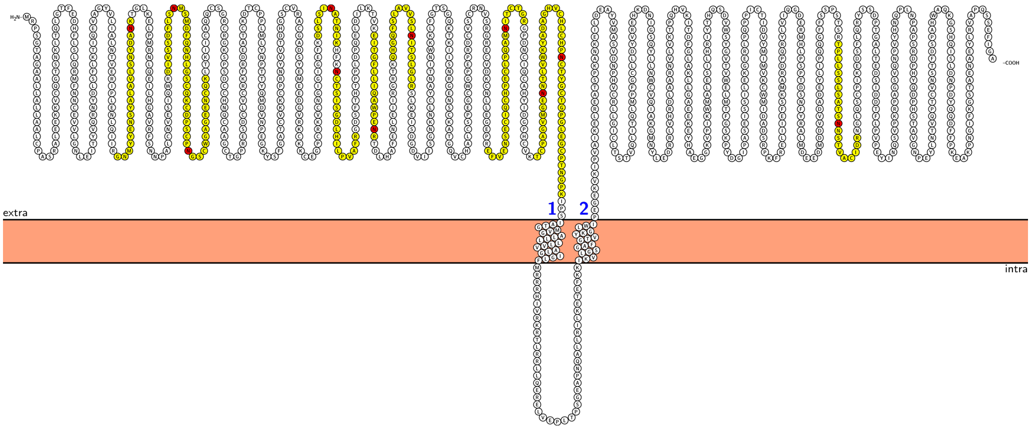

P04156

original Phobius topology

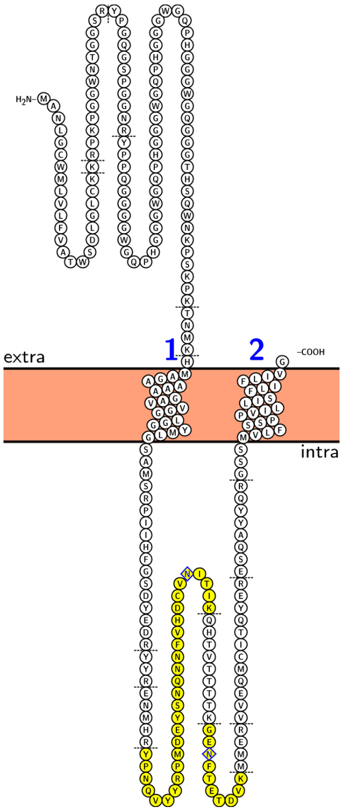

suggested corrected topology

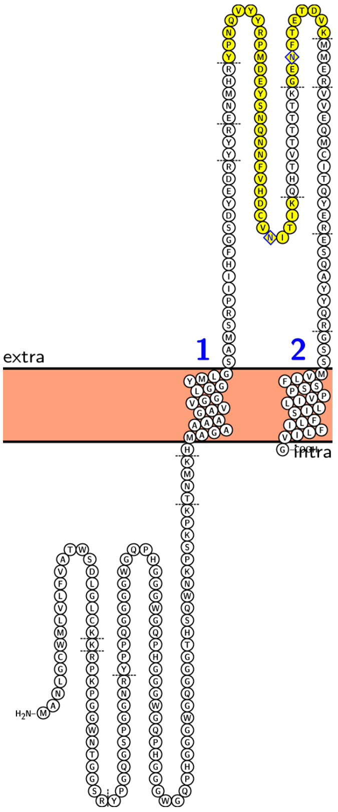

P05023

original Phobius topology

suggested corrected topology

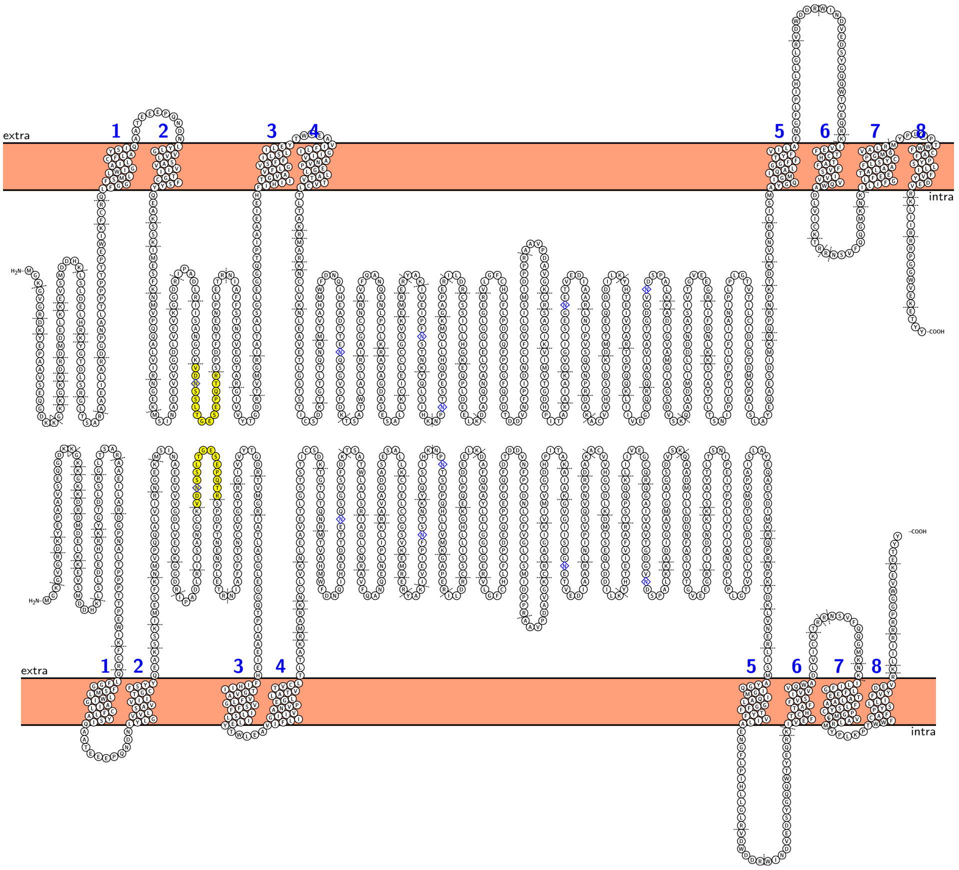

P08575

original Phobius topology

suggested corrected topology

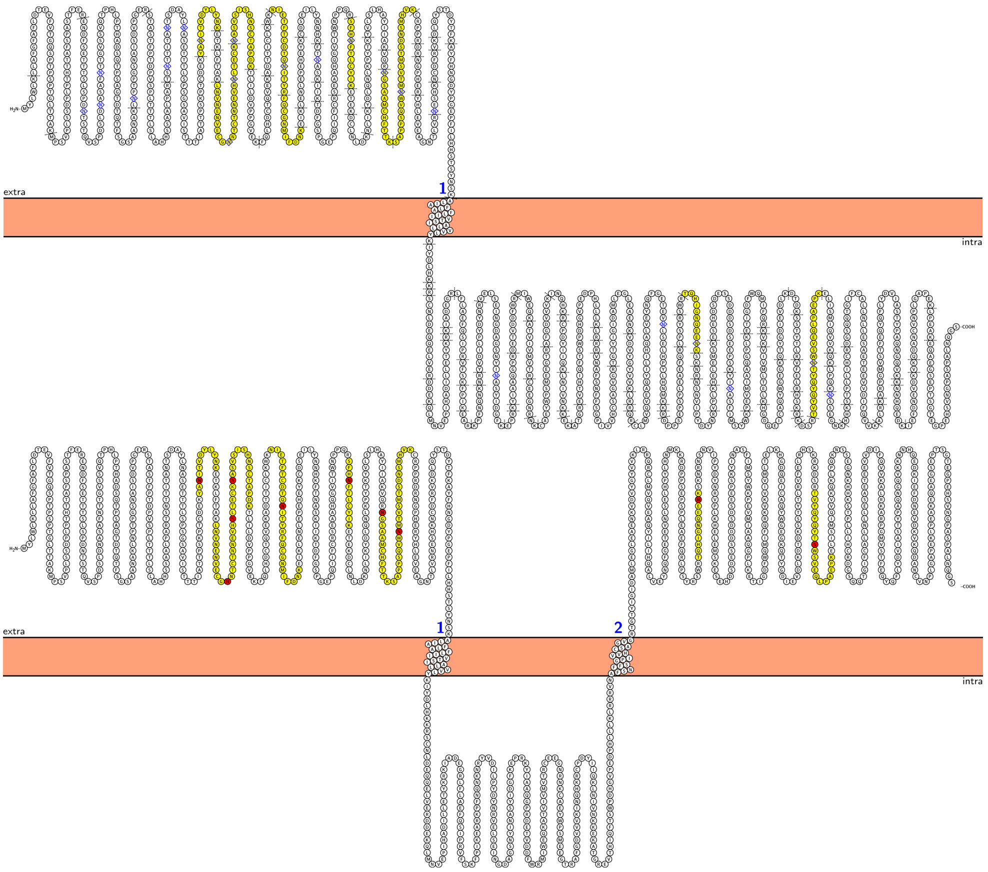

P09110

original Phobius topology

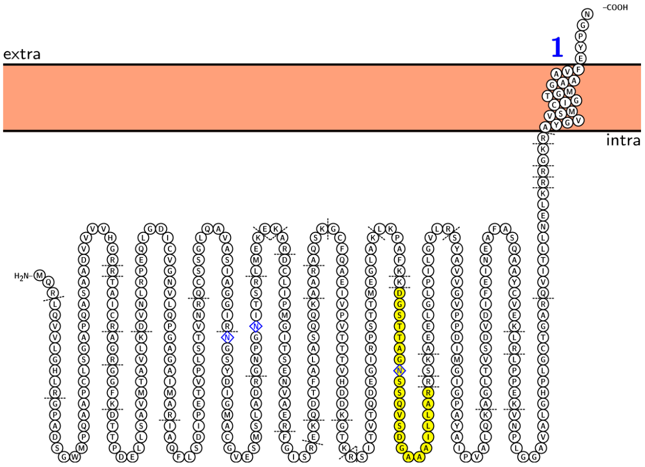

suggested corrected topology

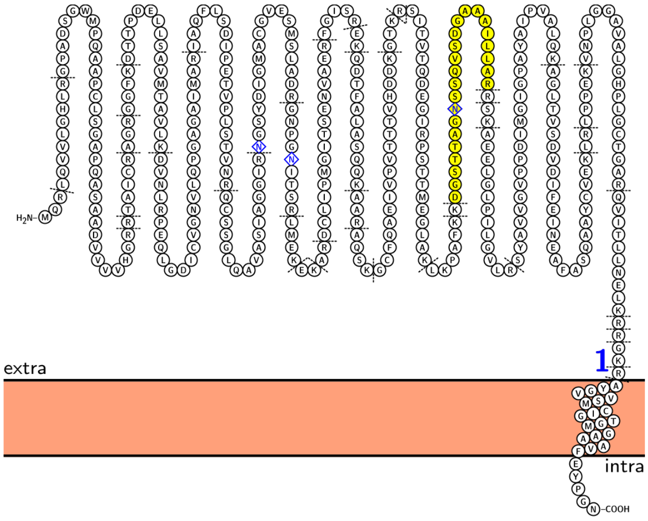

P16144

original Phobius topology

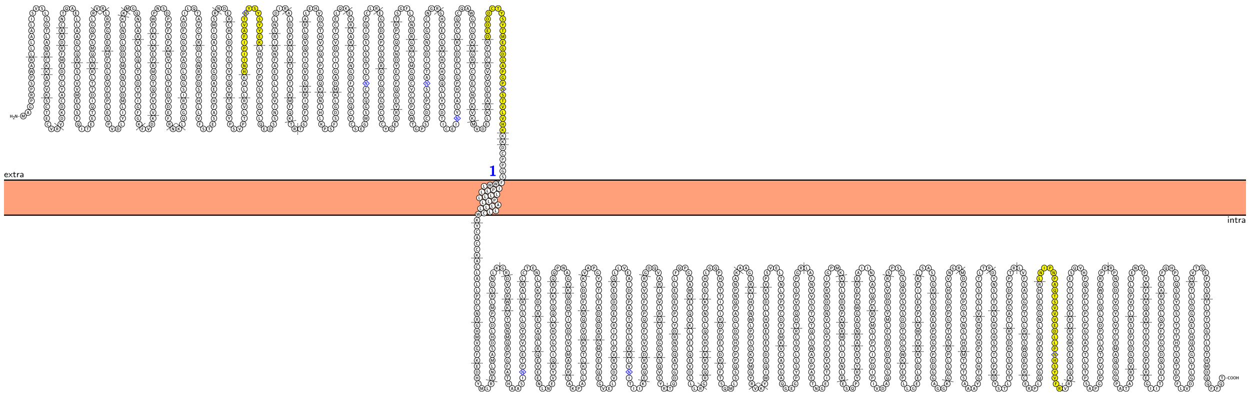

suggested corrected topology

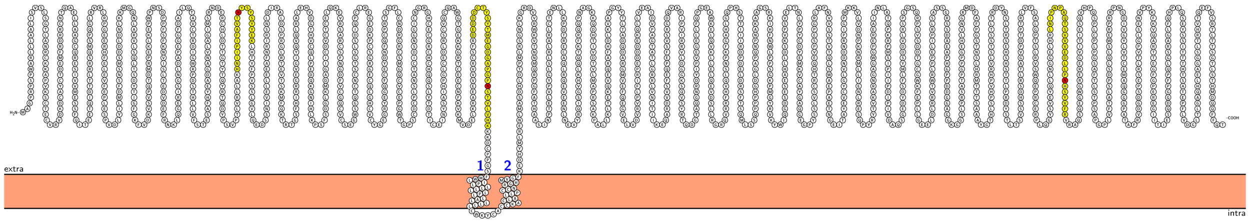

P17405

original Phobius topology

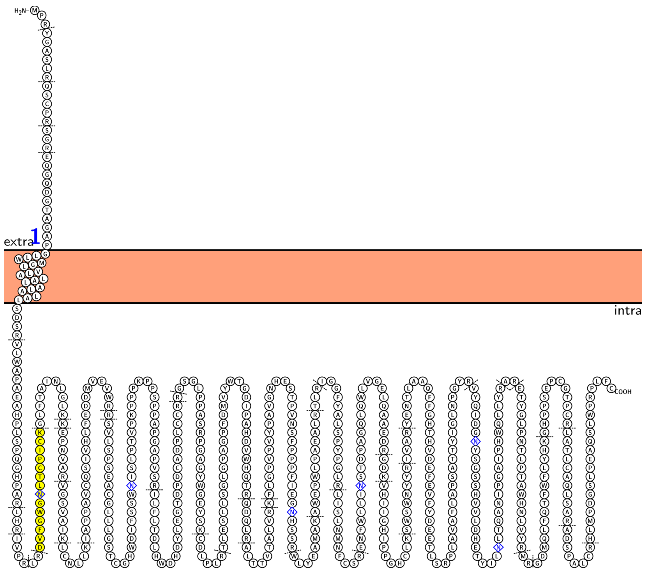

suggested corrected topology

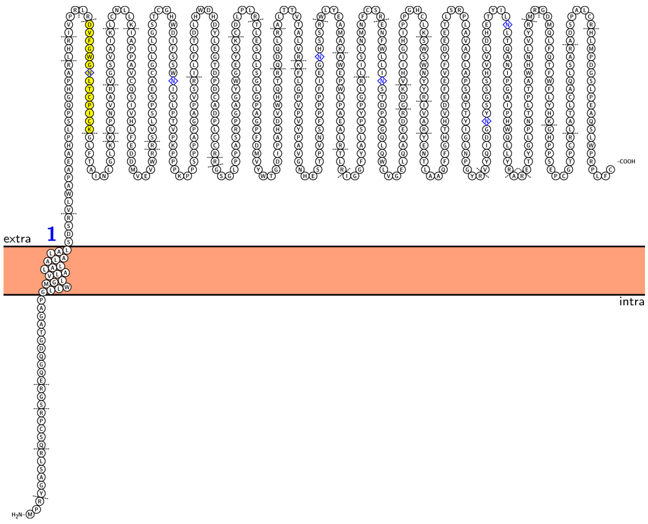

P27824

original Phobius topology

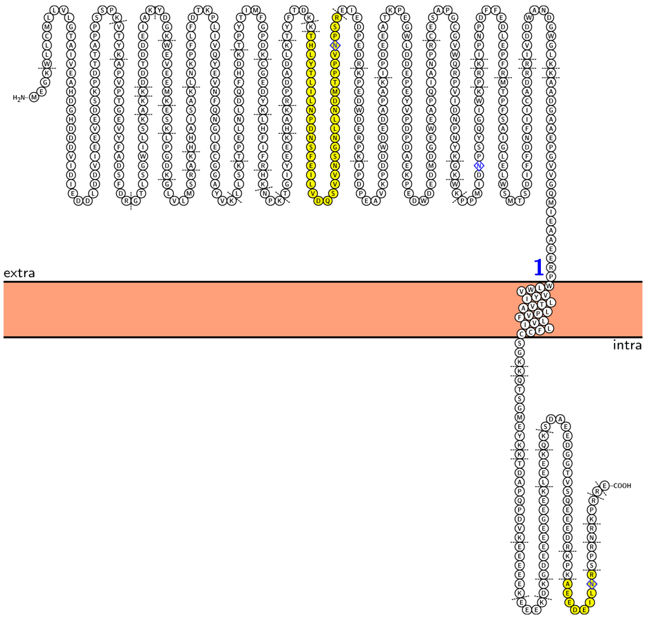

suggested corrected topology

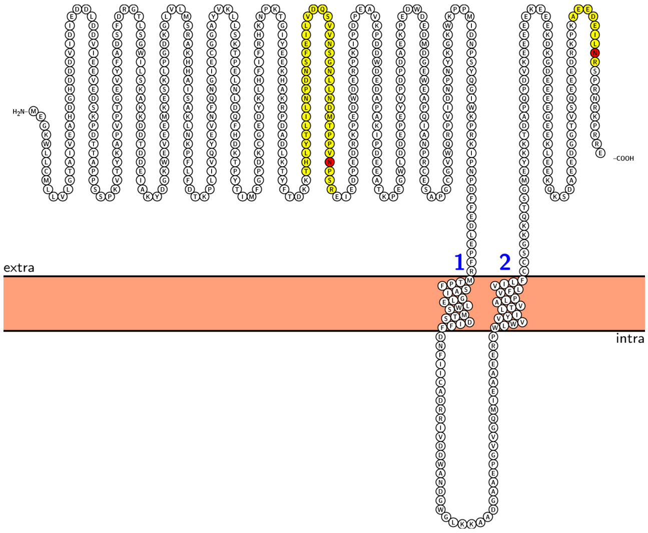

P34981

original Phobius topology

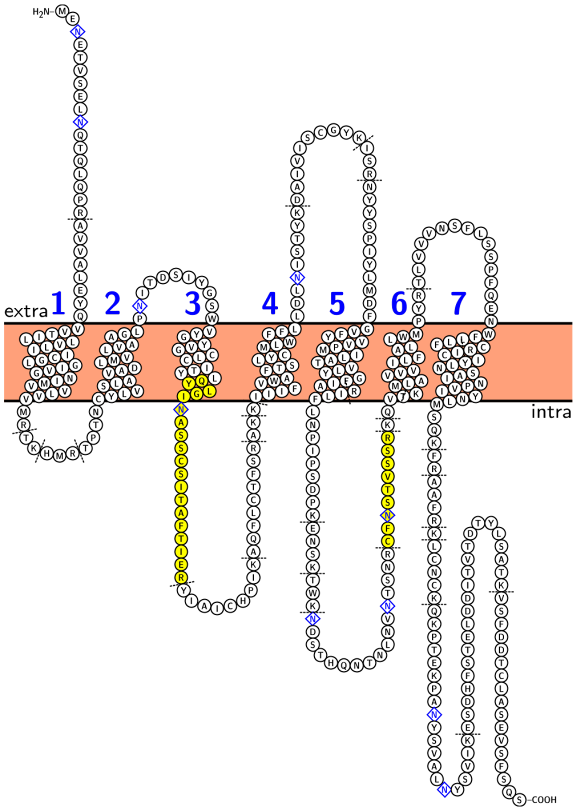

suggested corrected topology

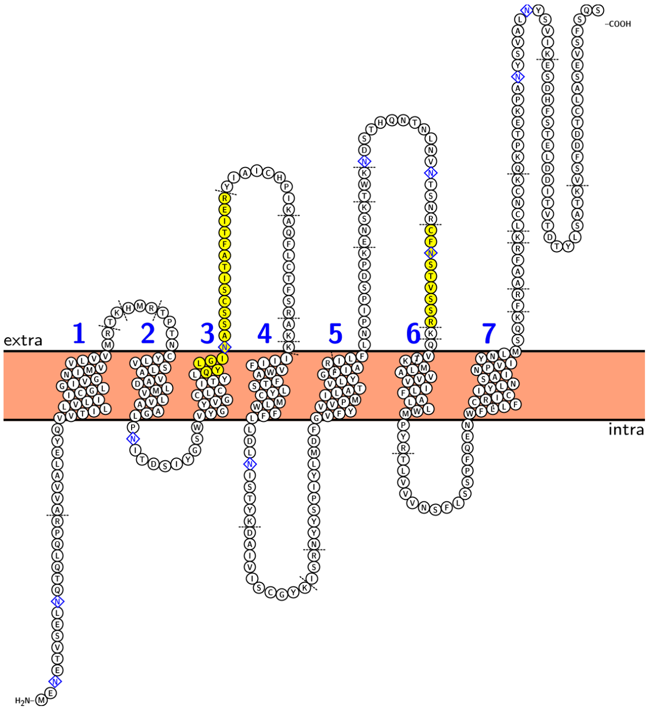

P37059

original Phobius topology

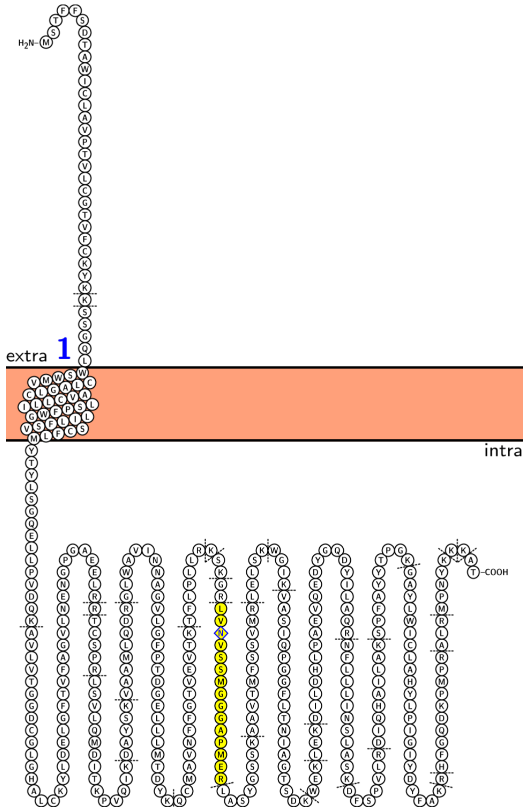

suggested corrected topology

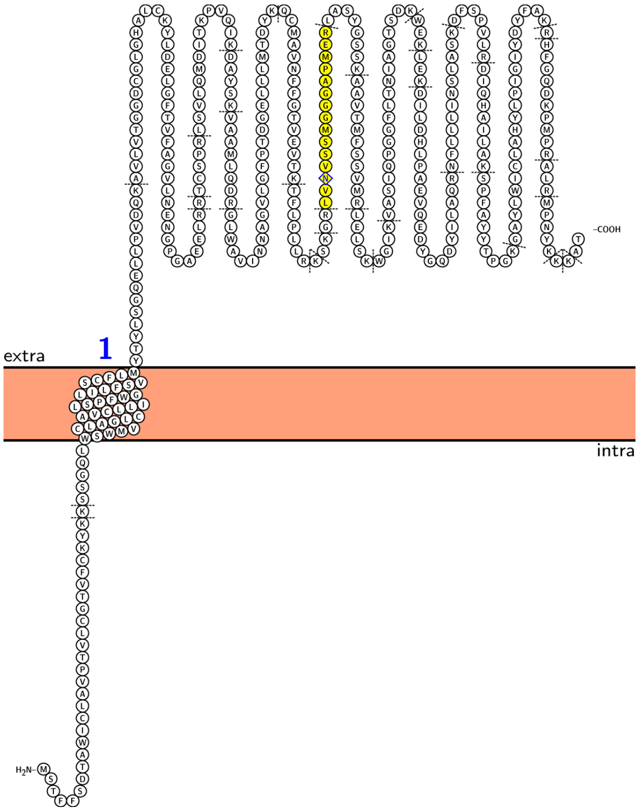

P43007

original Phobius topology

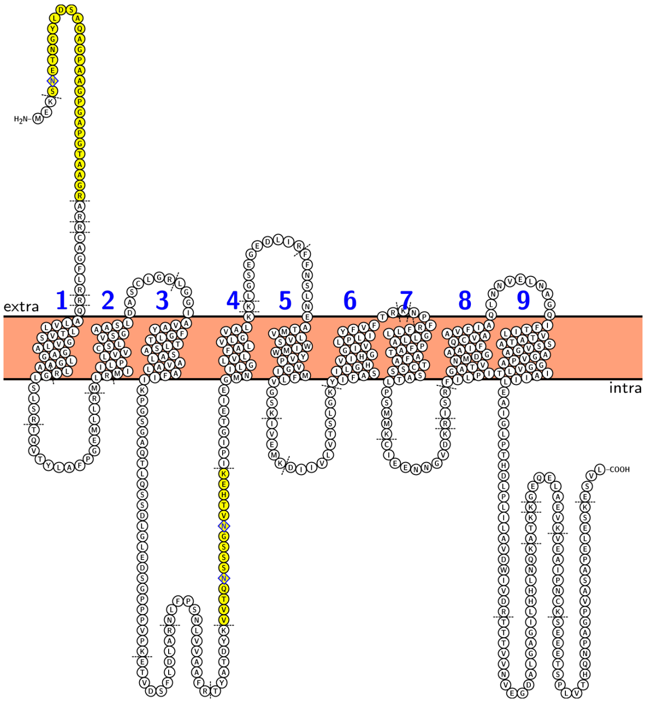

suggested corrected topology

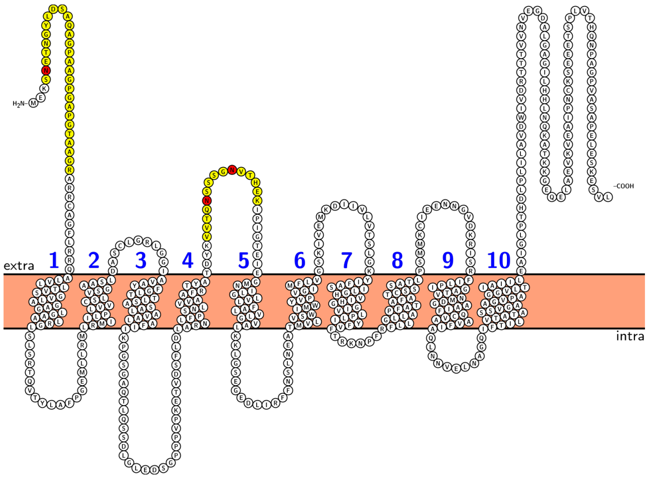

P50443

original Phobius topology

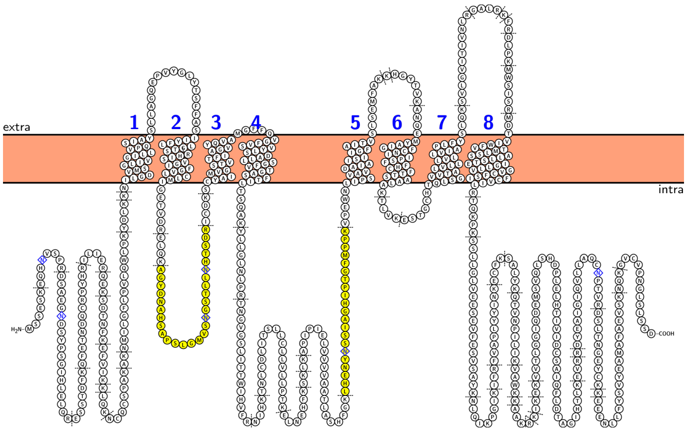

suggested corrected topology

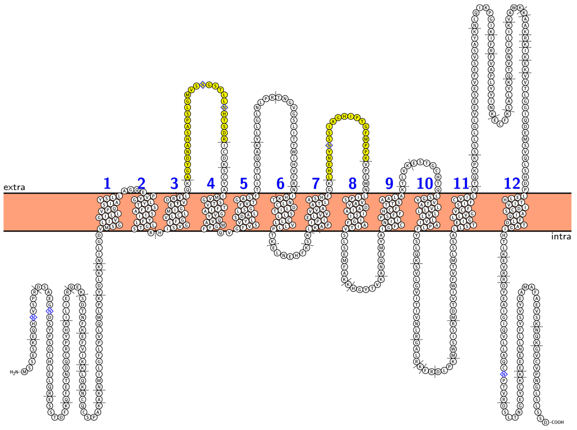

P56180

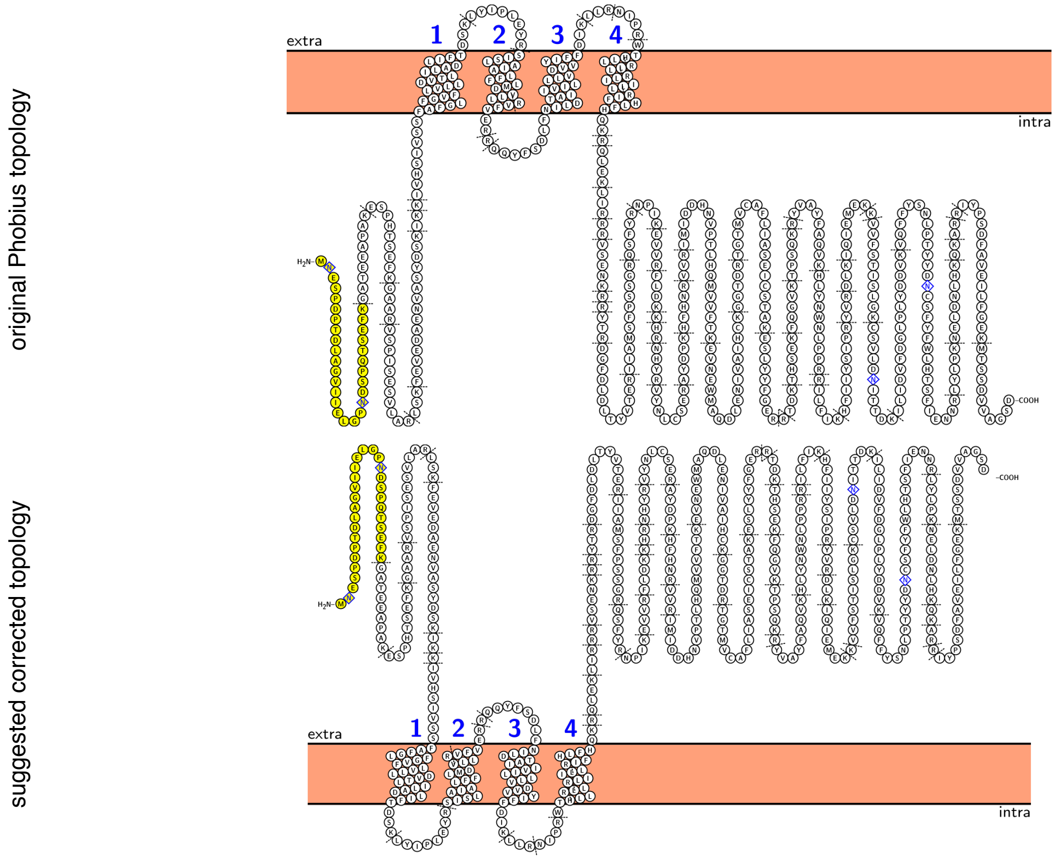

P56749

original Phobius topology

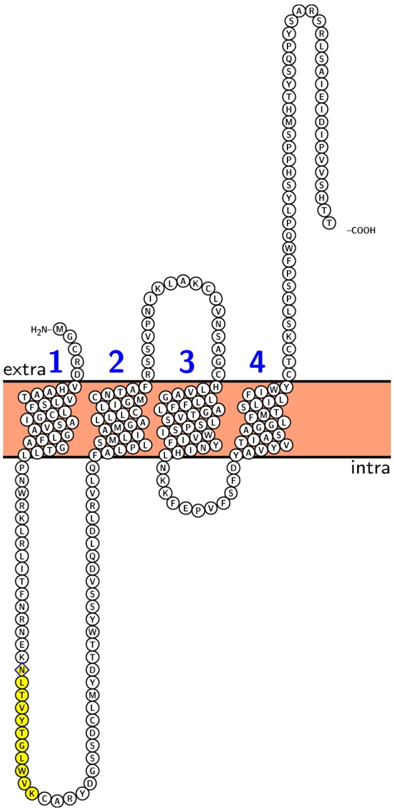

suggested corrected topology

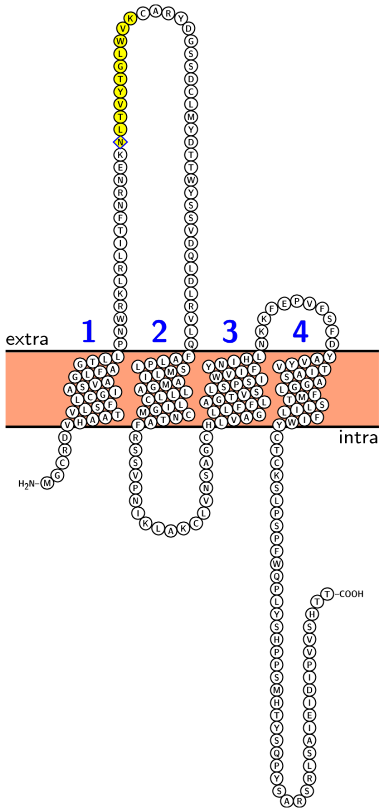

P58743

original Phobius topology

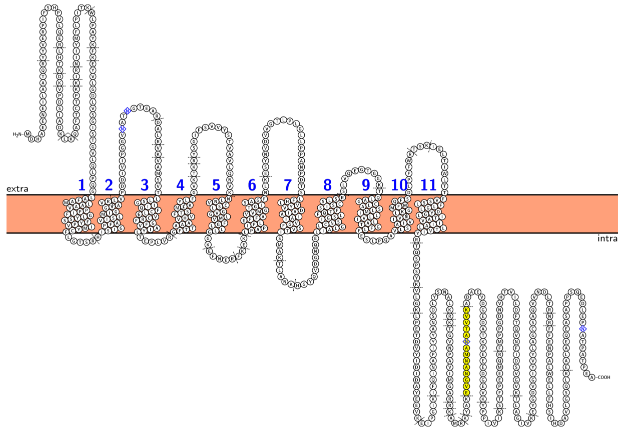

suggested corrected topology

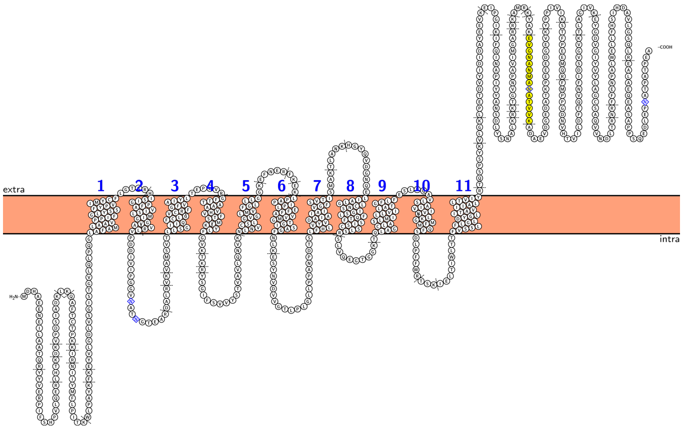

P81408

original Phobius topology

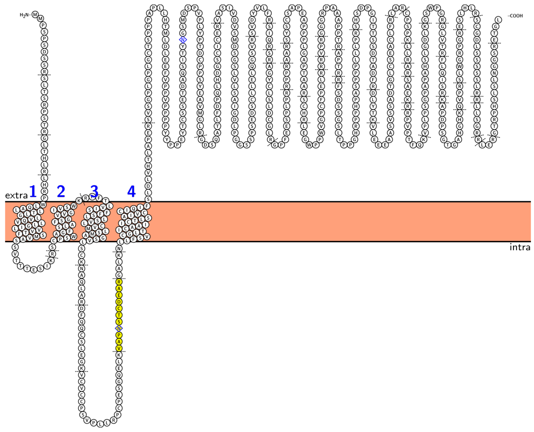

suggested corrected topology

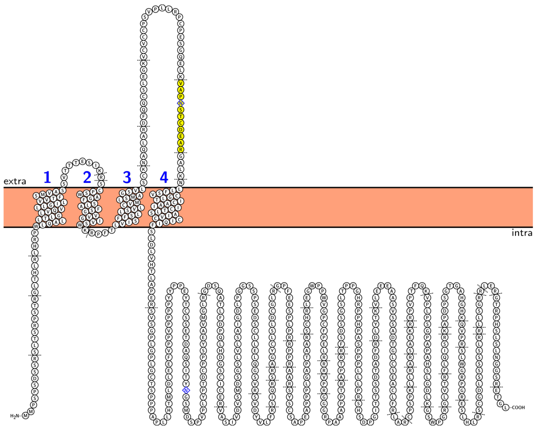

Q04941

original Phobius topology

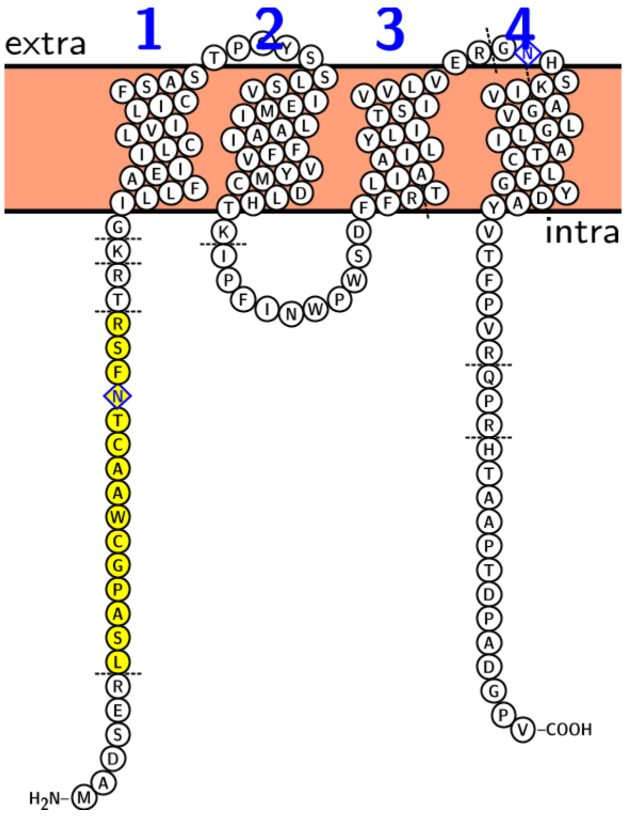

suggested corrected topology

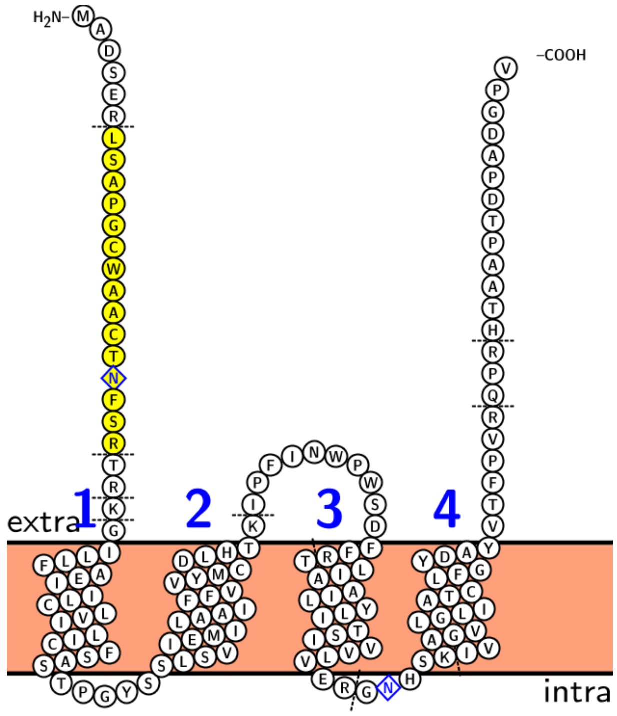

Q13286

original Phobius topology

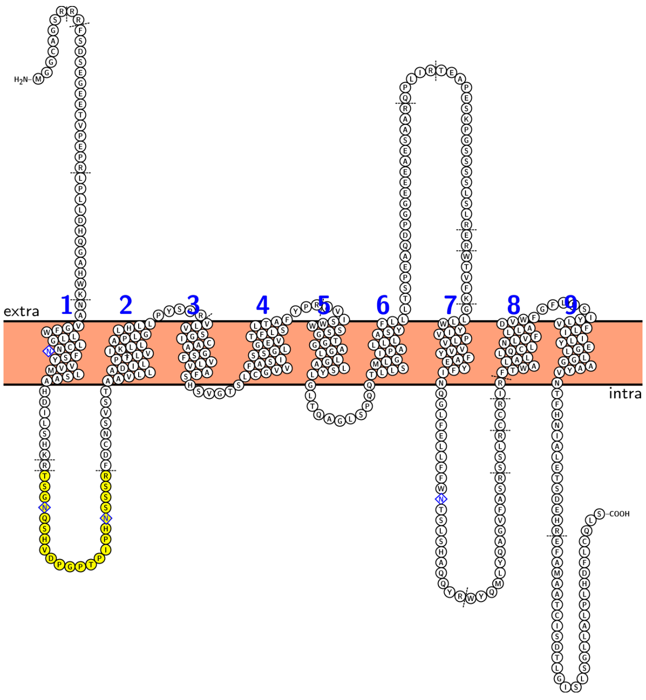

suggested corrected topology

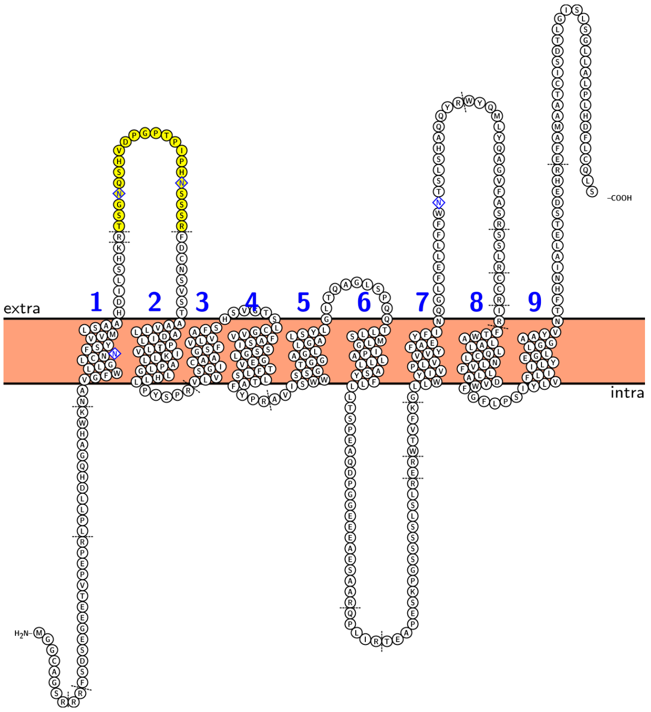

Q13336

original Phobius topology

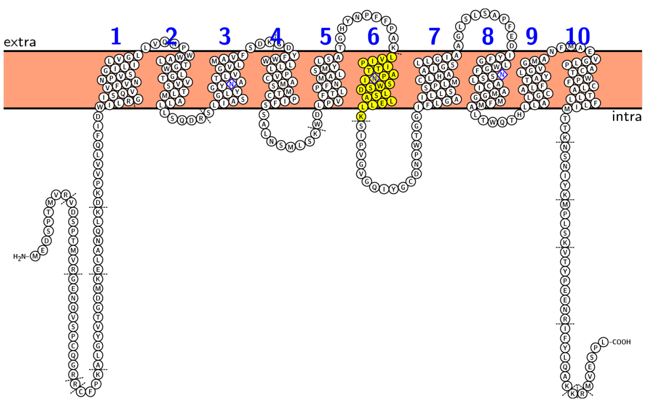

suggested corrected topology

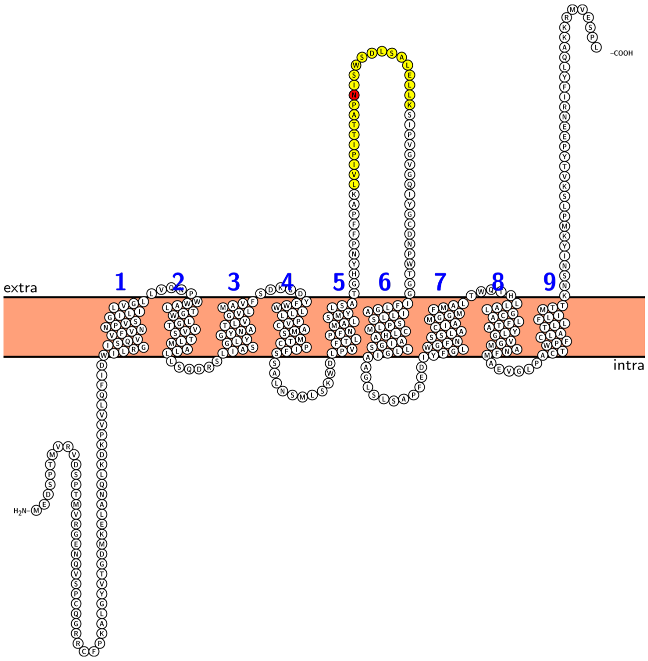

Q14314

original Phobius topology

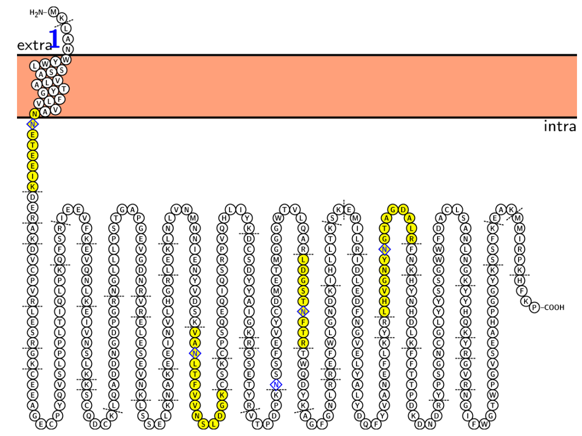

suggested corrected topology

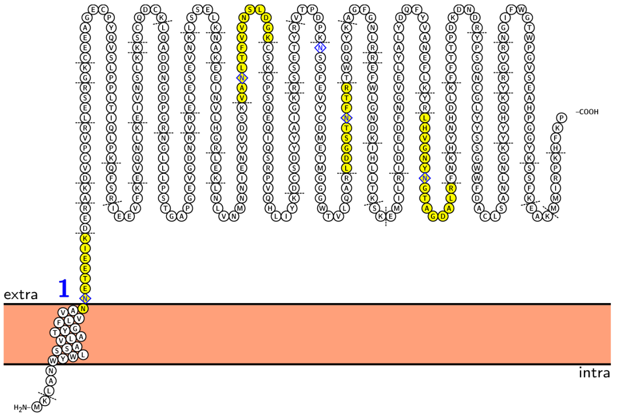

Q3MIRA

original Phobius topology

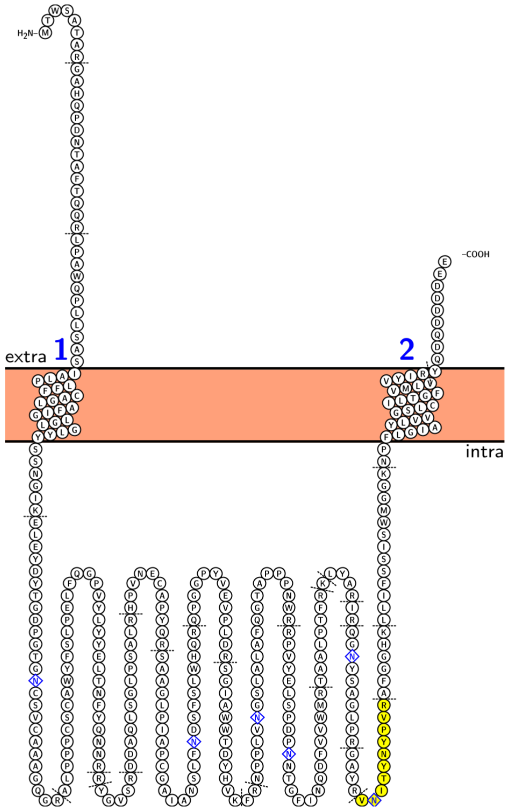

suggested corrected topology

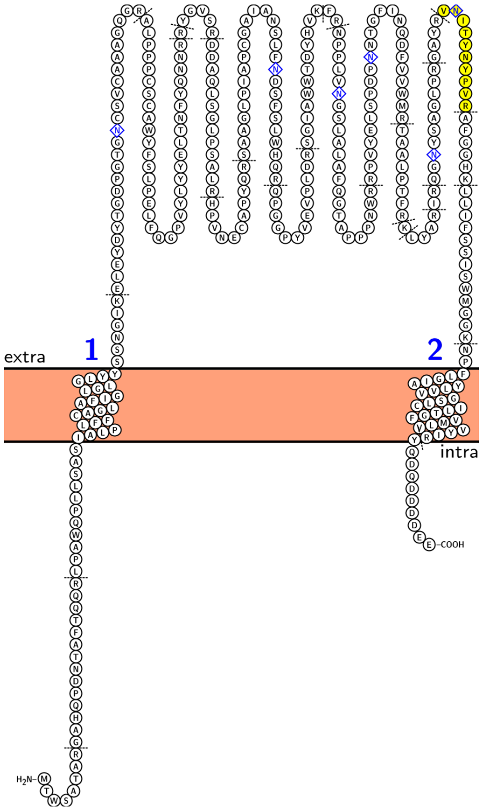

Q6ZS10

original Phobius topology

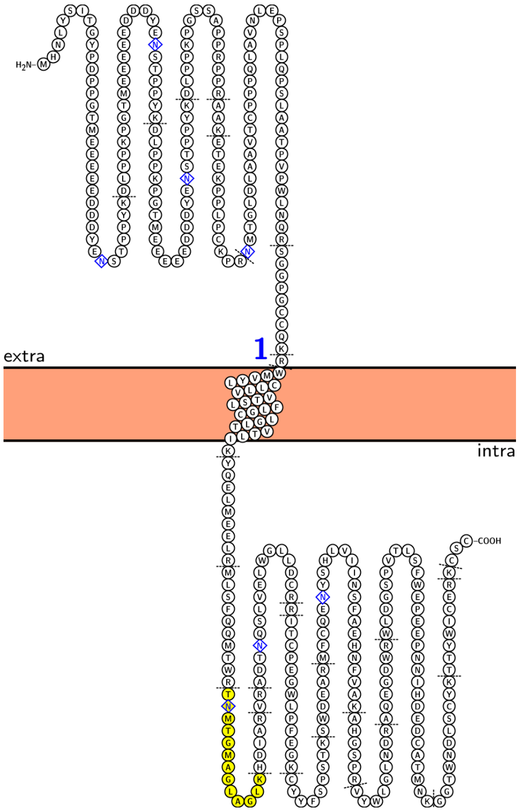

suggested corrected topology

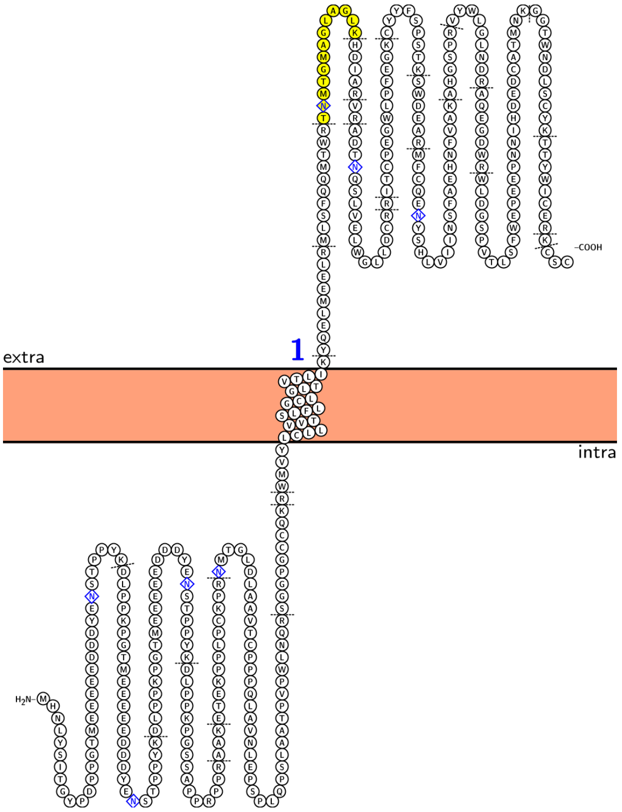

Q7Z388

original Phobius topology

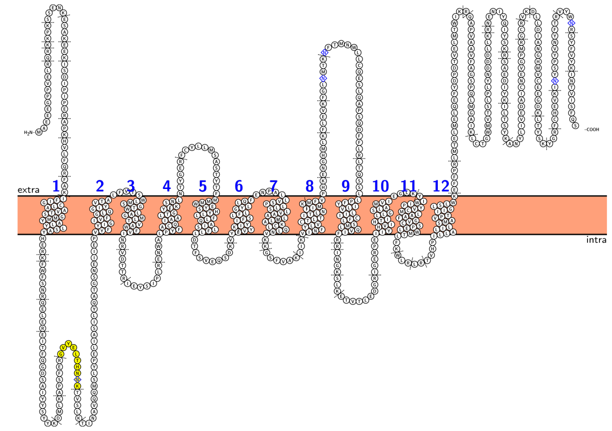

suggested corrected topology

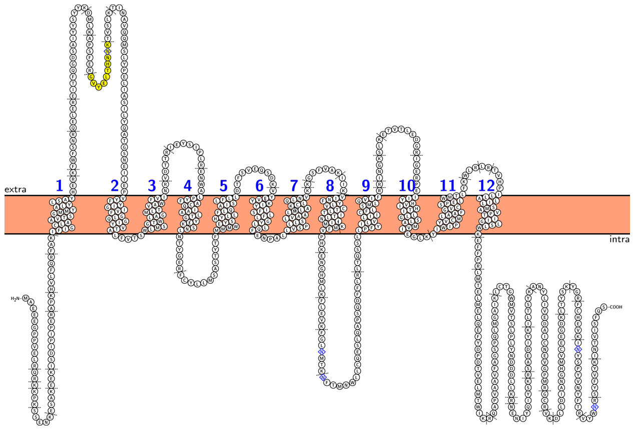

Q8IYP9

original Phobius topology

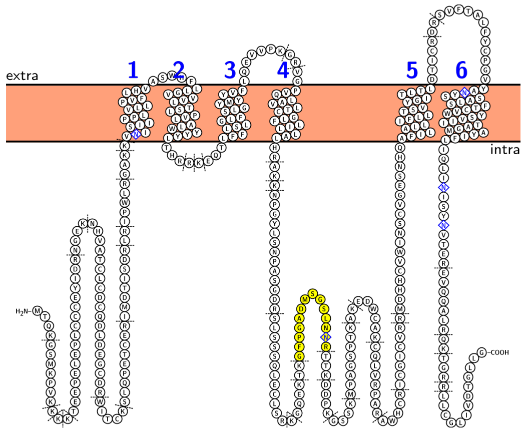

suggested corrected topology

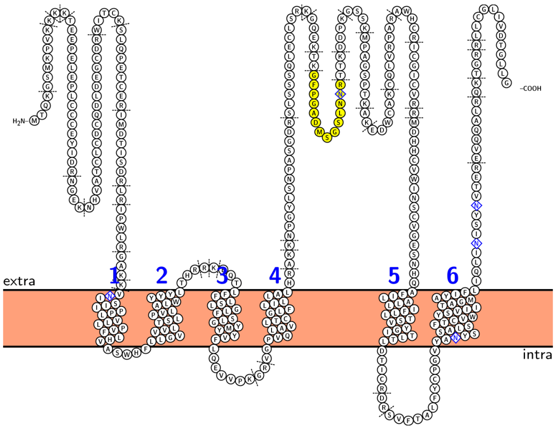

Q8NBI5

original Phobius topology

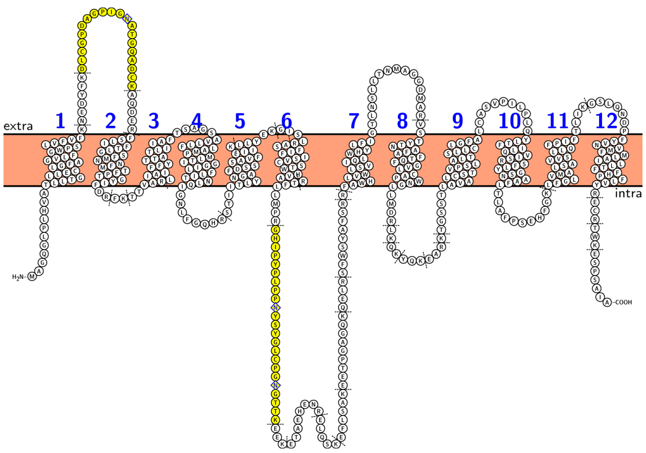

suggested corrected topology

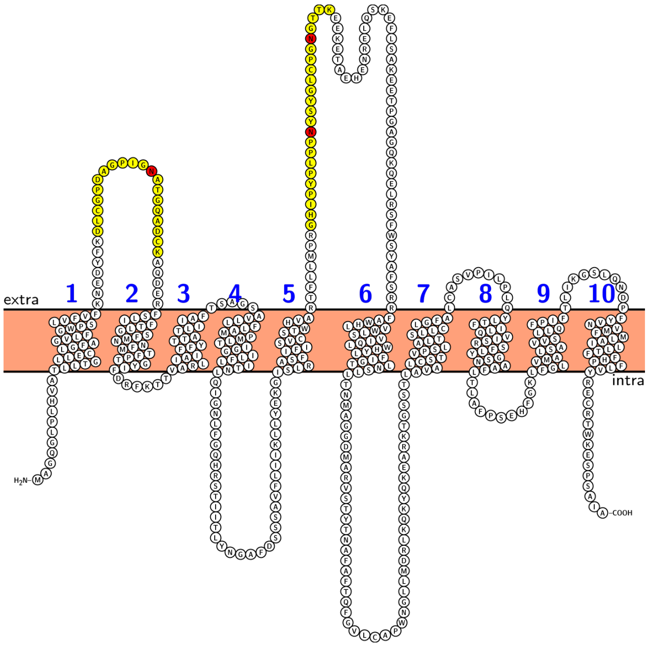

Q8N4VI

original Phobius topology

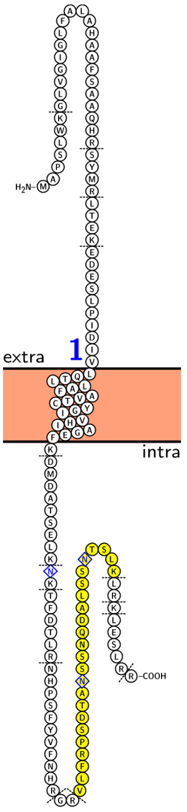

suggested corrected topology

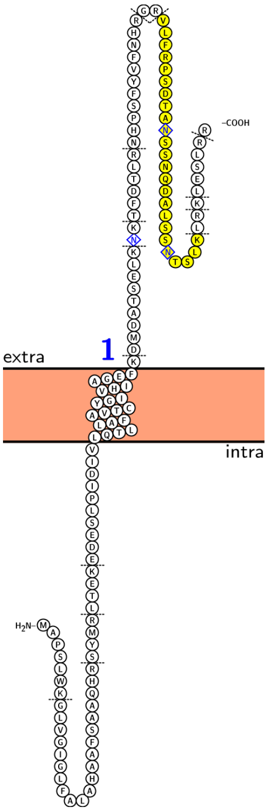

Q8NG11

original Phobius topology

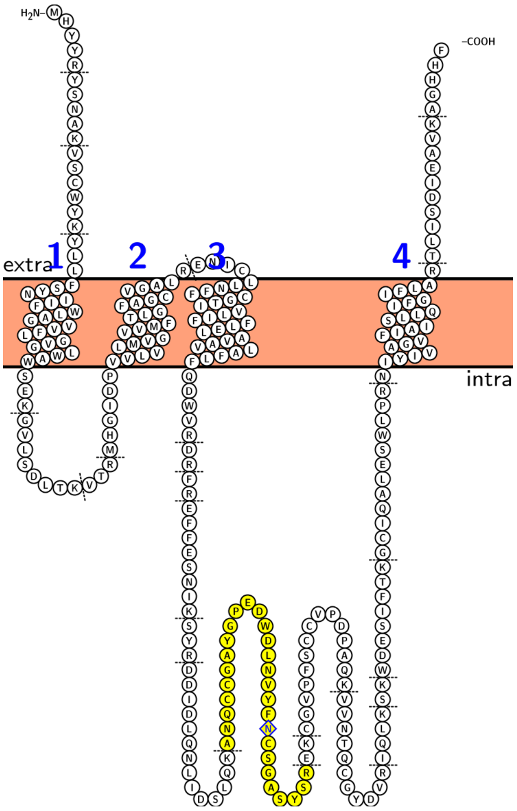

suggested corrected topology

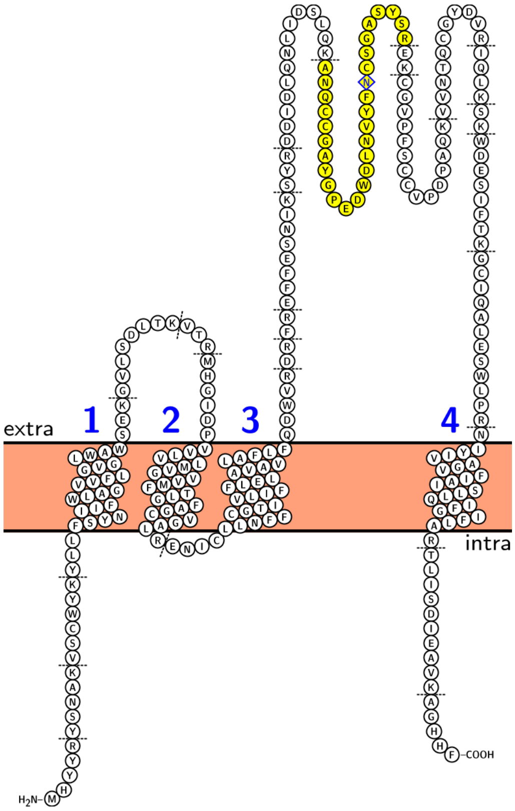

Q8TDW0

original Phobius topology

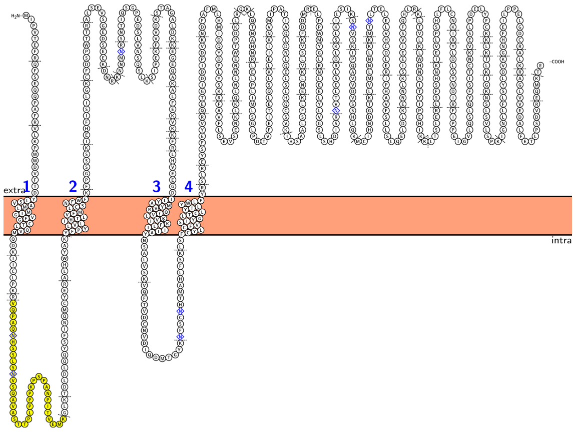

suggested corrected topology

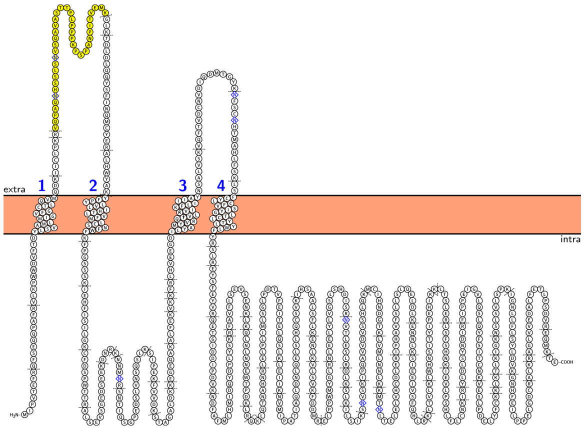

Q93050

original Phobius topology

suggested corrected topology

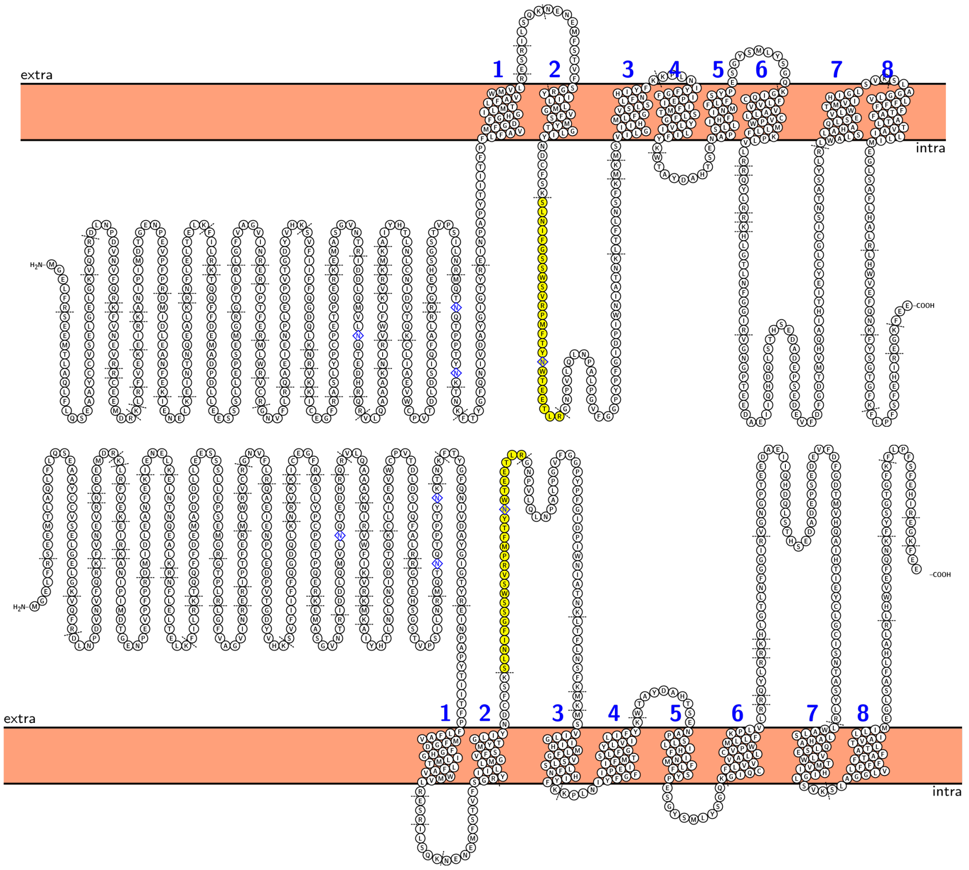

Q96T54

original Phobius topology

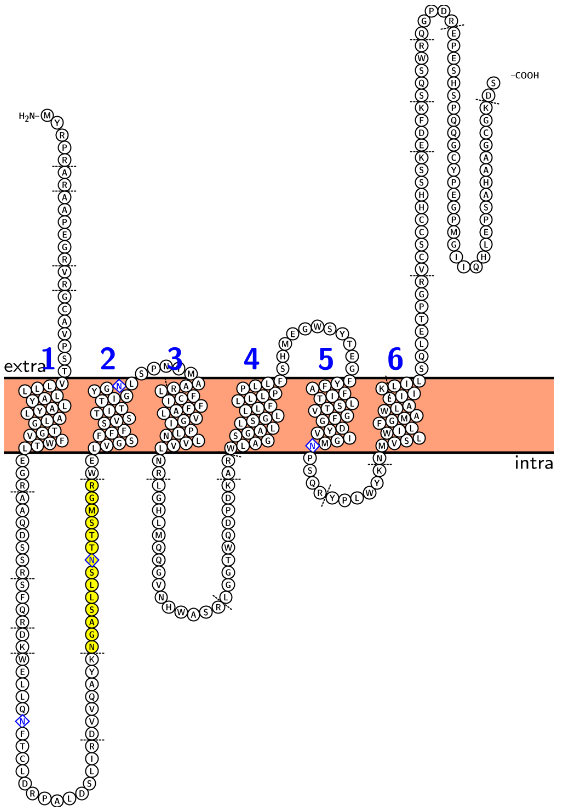

suggested corrected topology

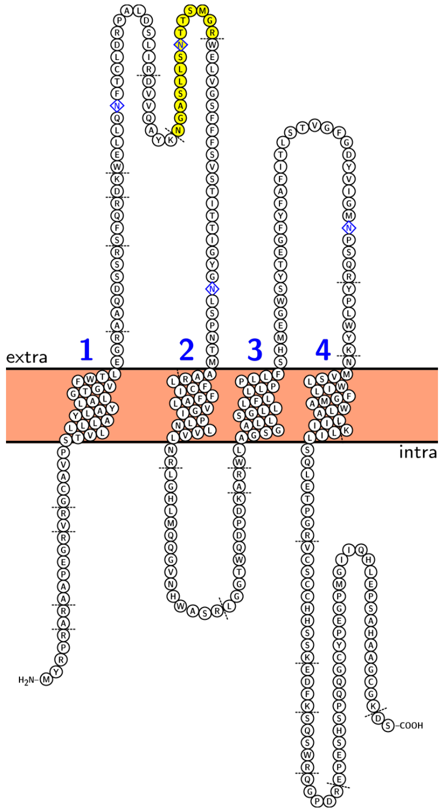

Q9BU23

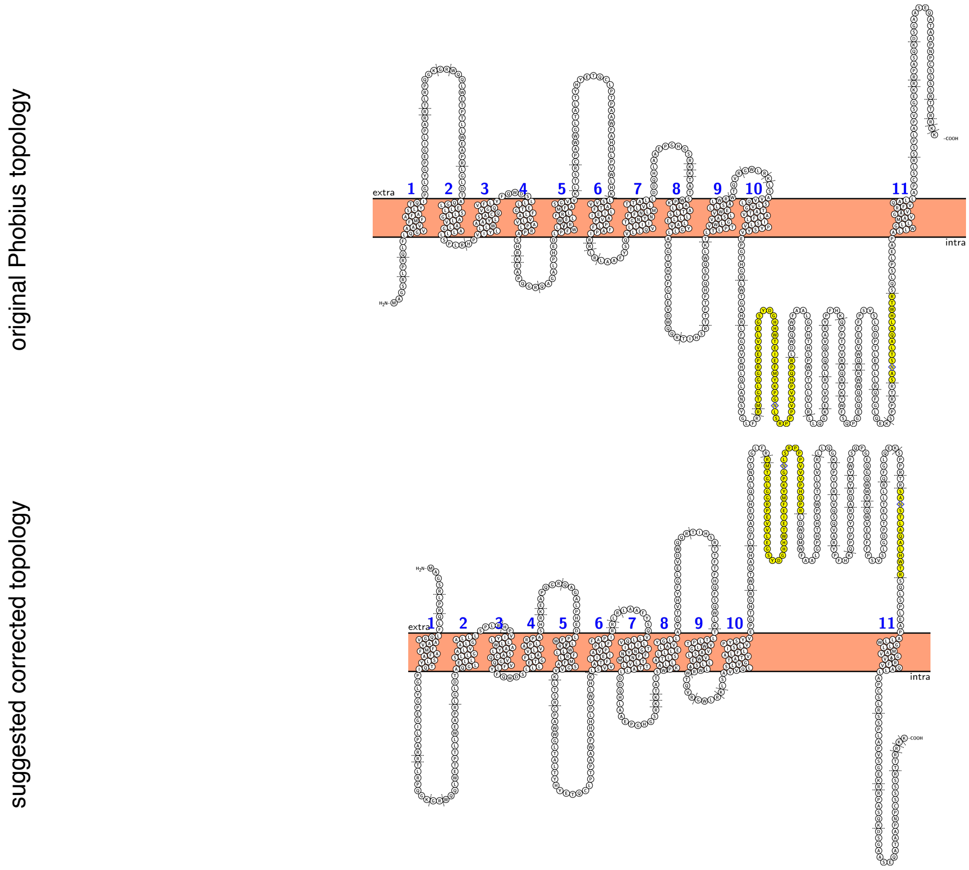

Q9BX74

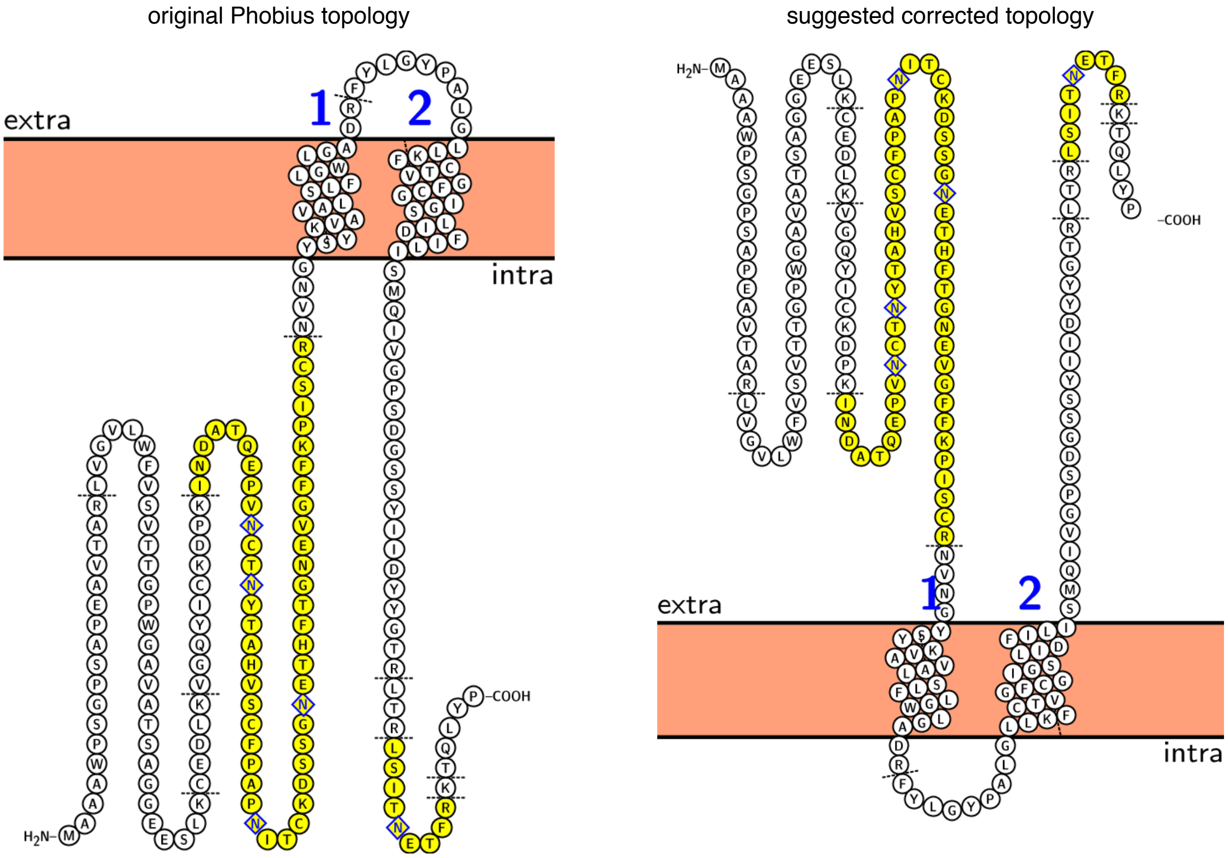

Q9BXS9

original Phobius topology

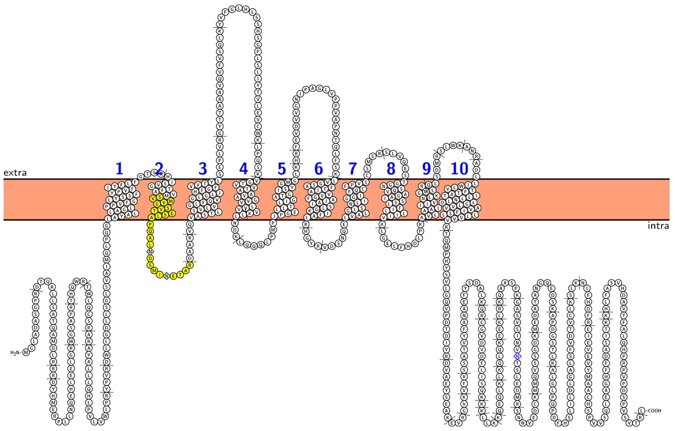

suggested corrected topology

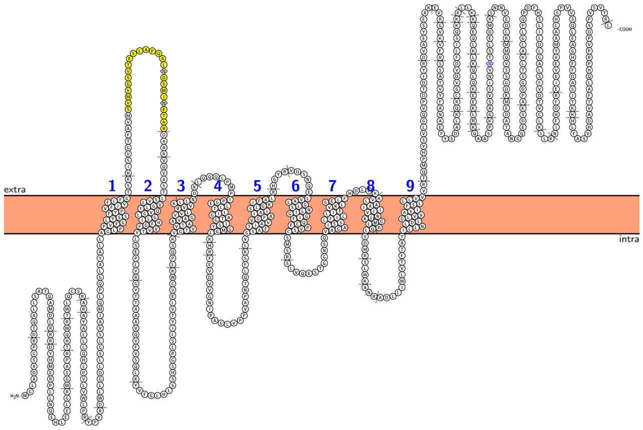

Q9BXT2

original Phobius topology

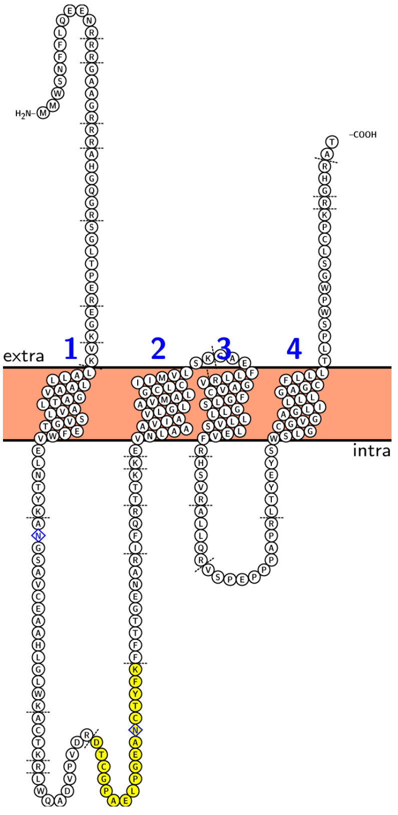

suggested corrected topology

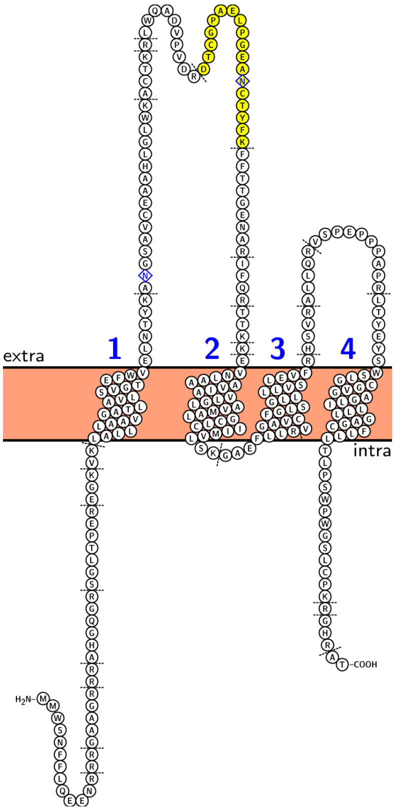

Q9C0B5

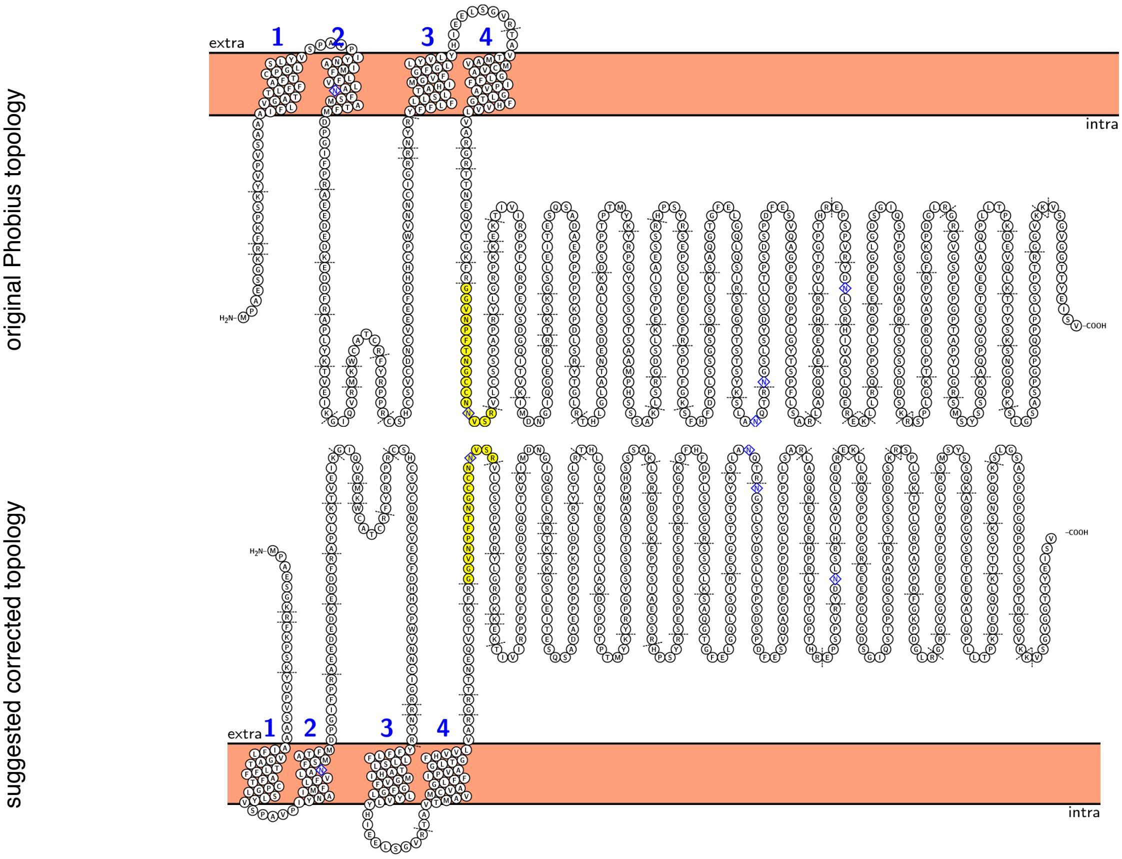

Q9GZU1

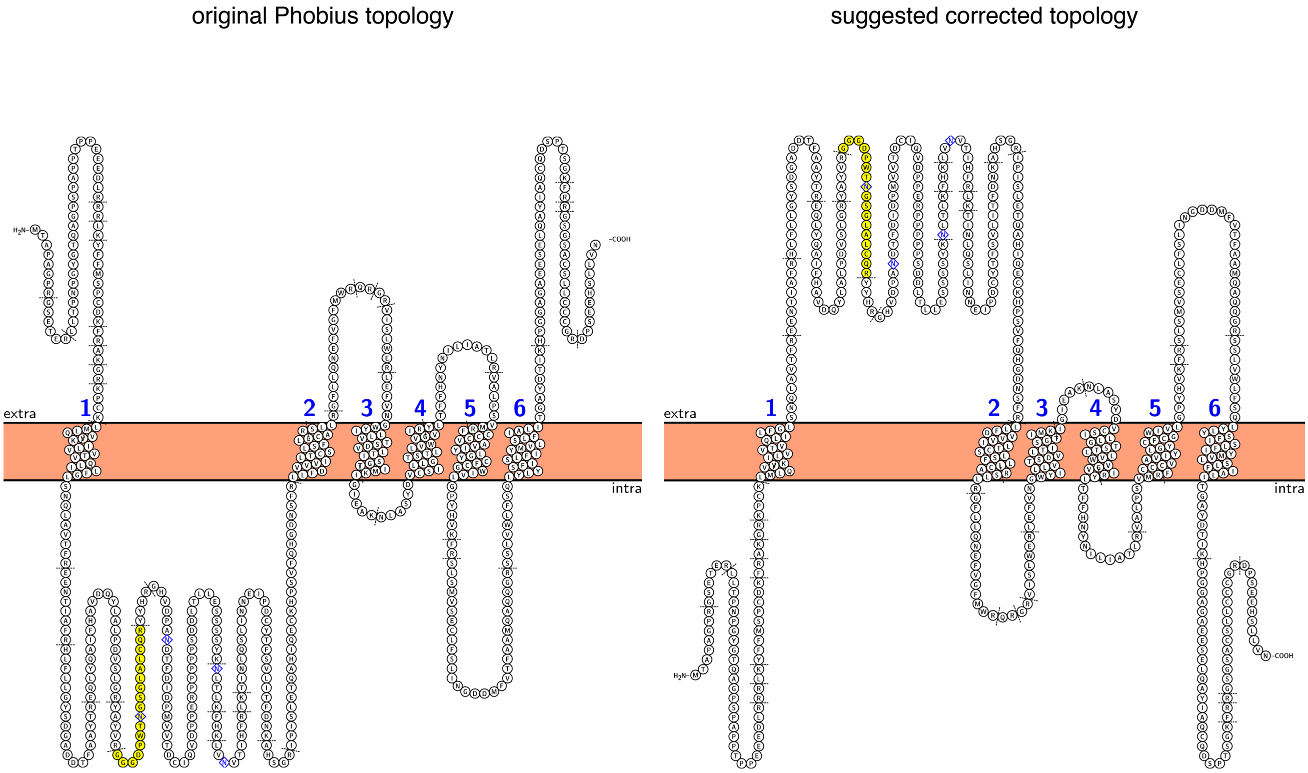

Q9H1C3

original Phobius topology

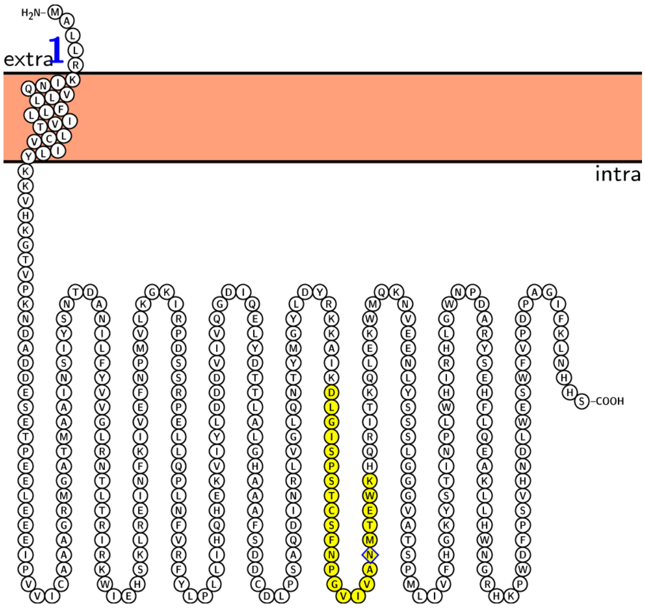

suggested corrected topology

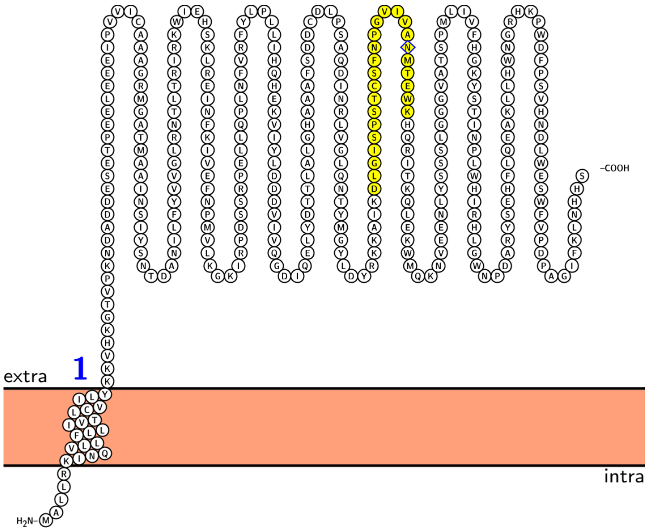

Q9H330

original Phobius topology

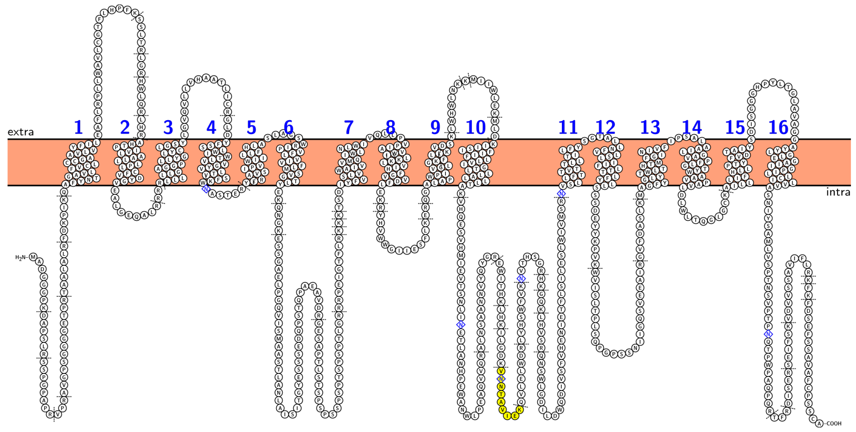

suggested corrected topology

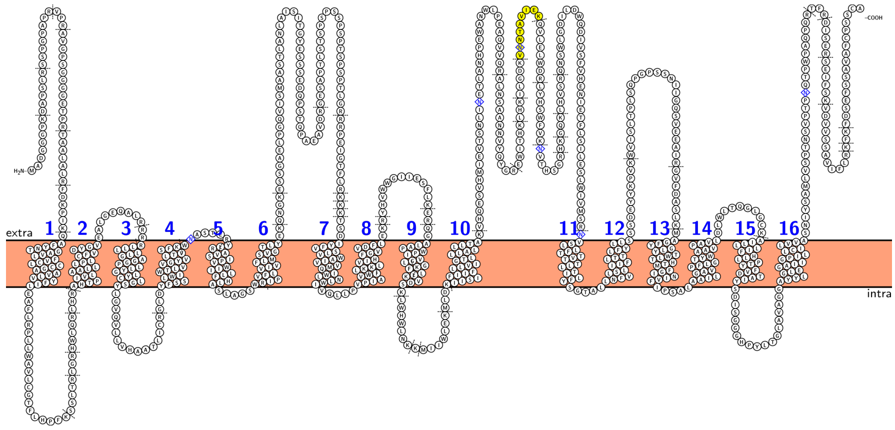

Q9H6L2

original Phobius topology

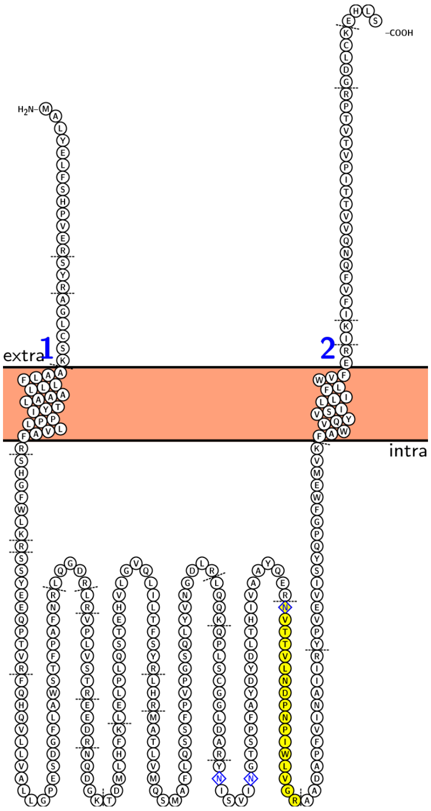

suggested corrected topology

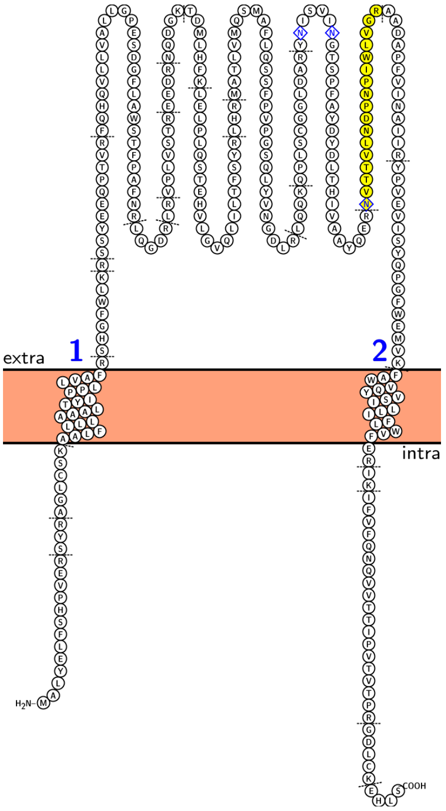

Q9HCM3

original Phobius topology

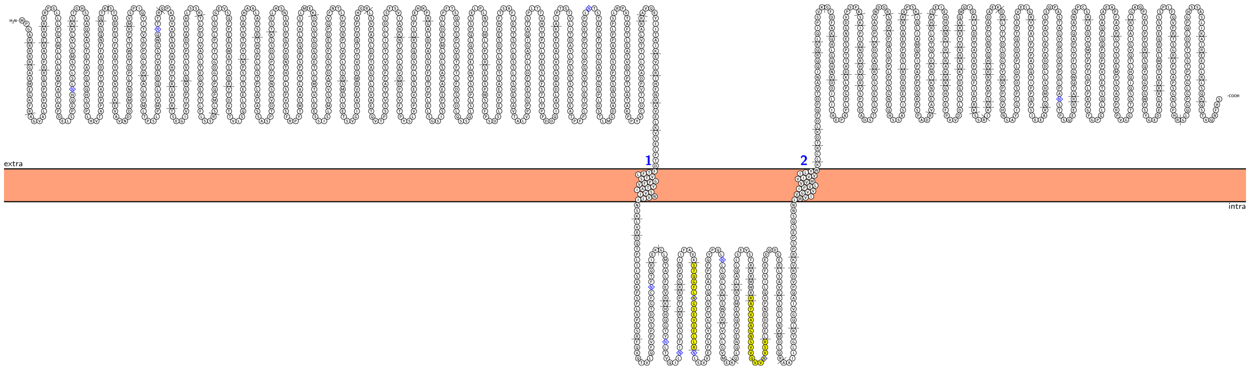

suggested corrected topology

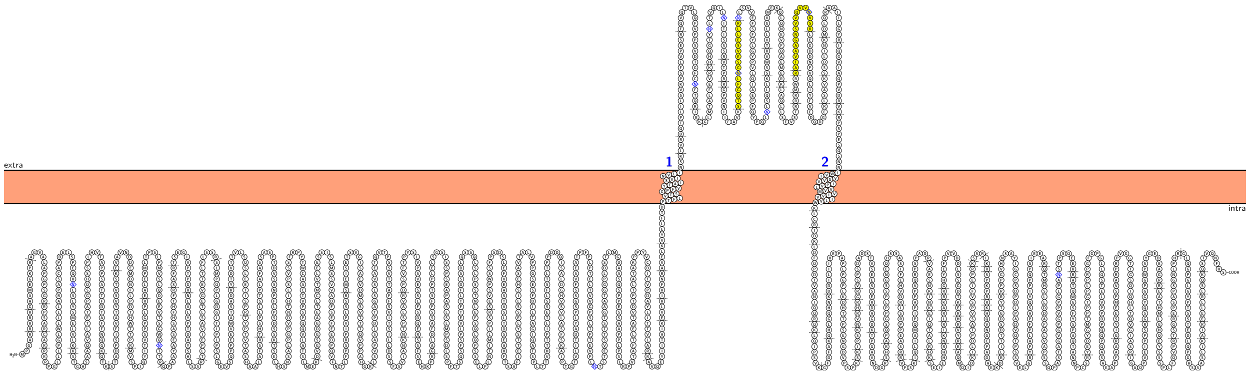

Q9HD45

original Phobius topology

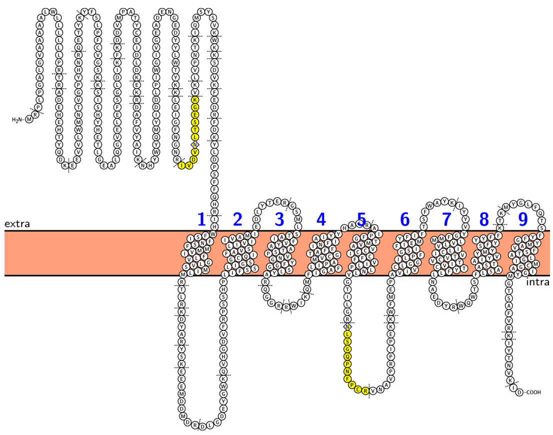

suggested corrected topology

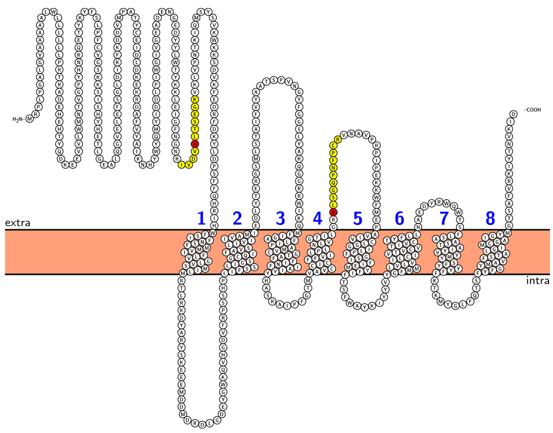

Q9NWD8

original Phobius topology

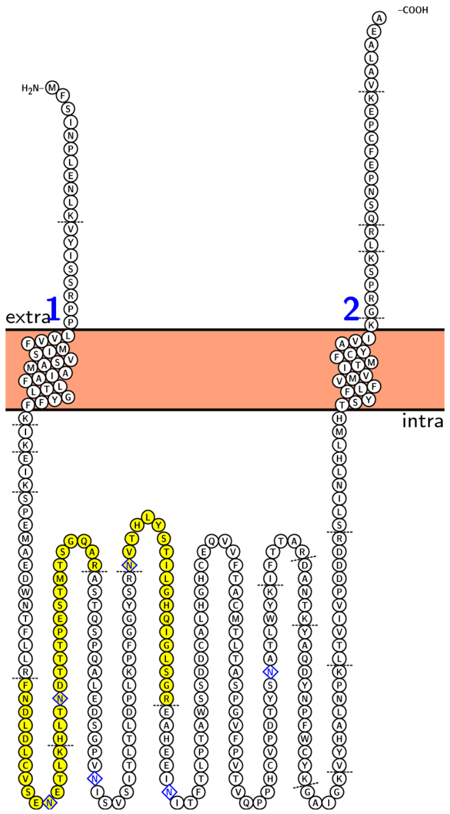

suggested corrected topology

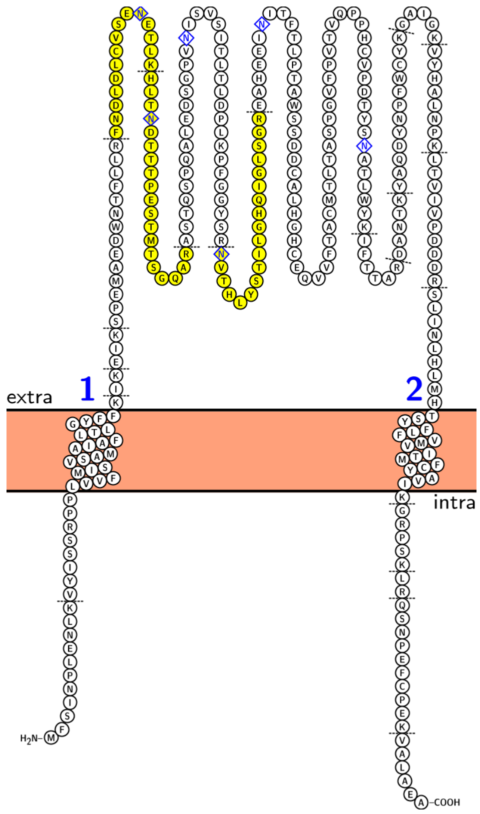

Q9UHW9

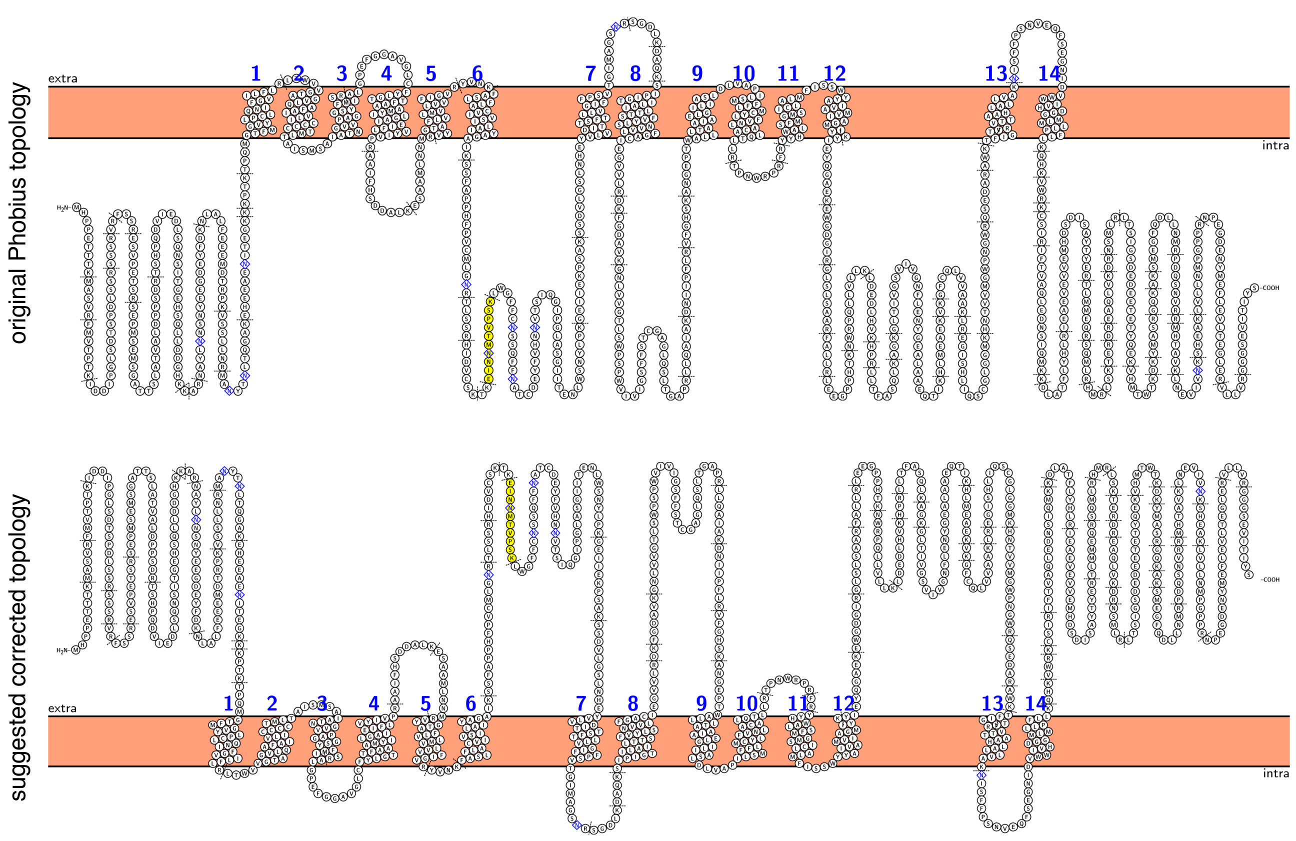

Q9UKY4

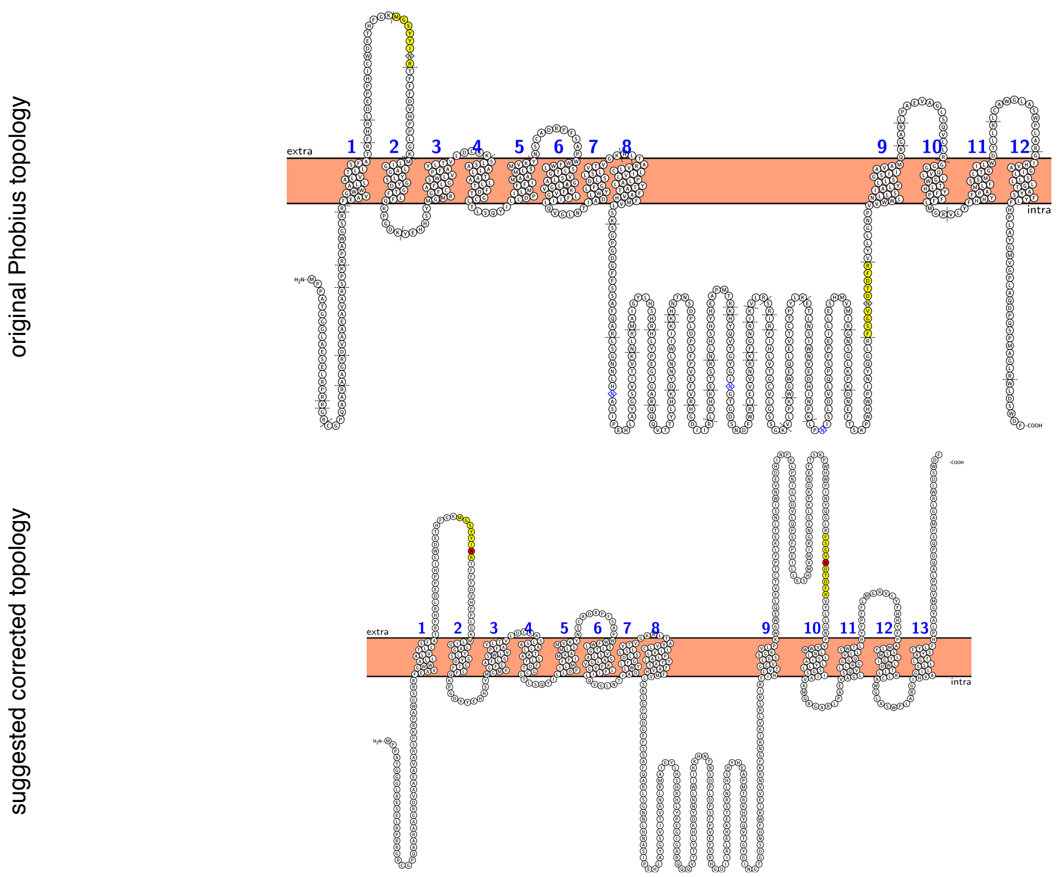

Q9UN70

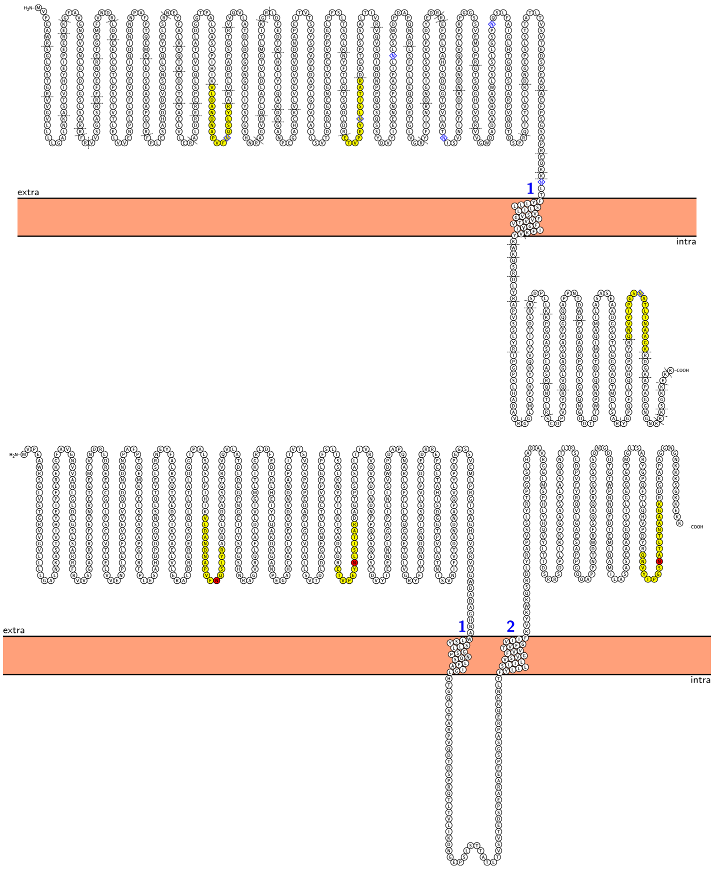

Q9Y6A1

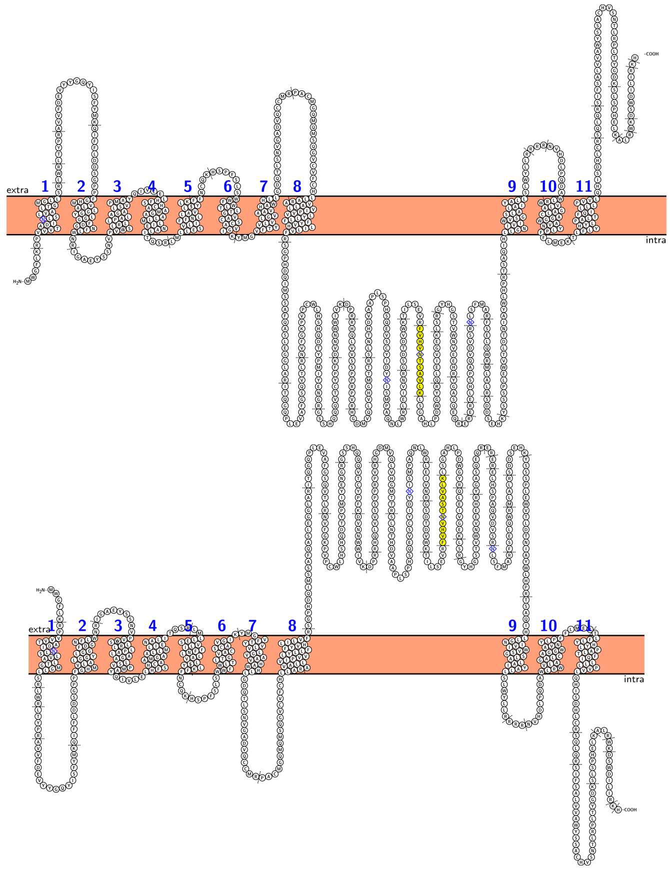

corrected topology
of mouse proteins

A2AJQ3

original Phobius topology

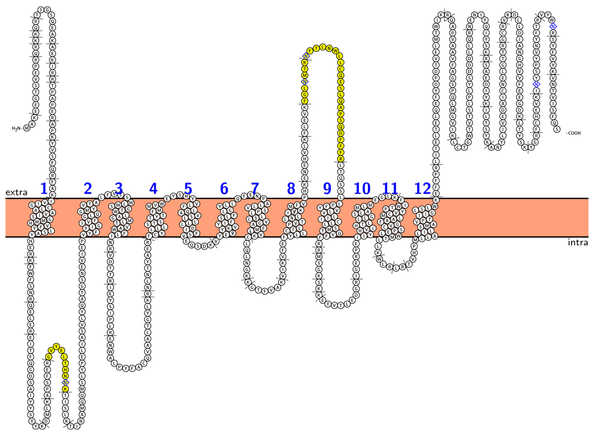

suggested corrected topology

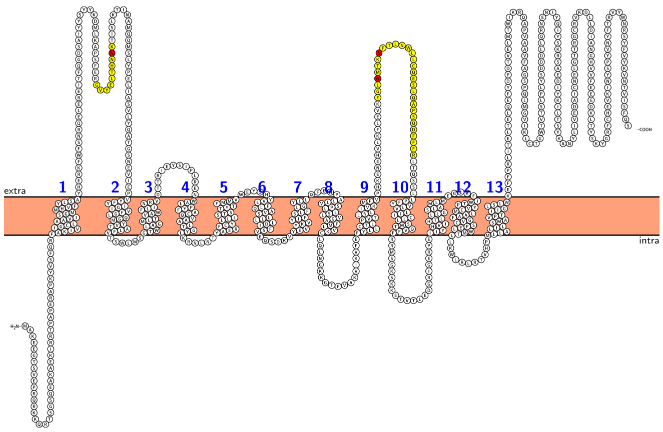

P04925

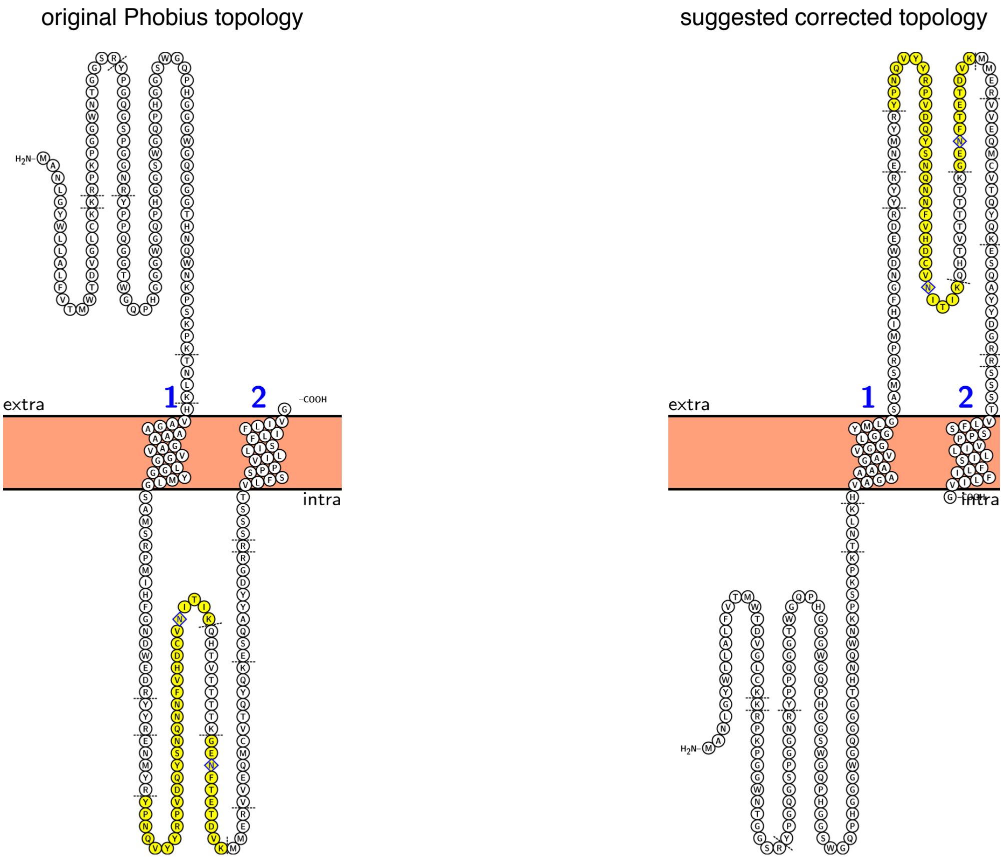

P46978

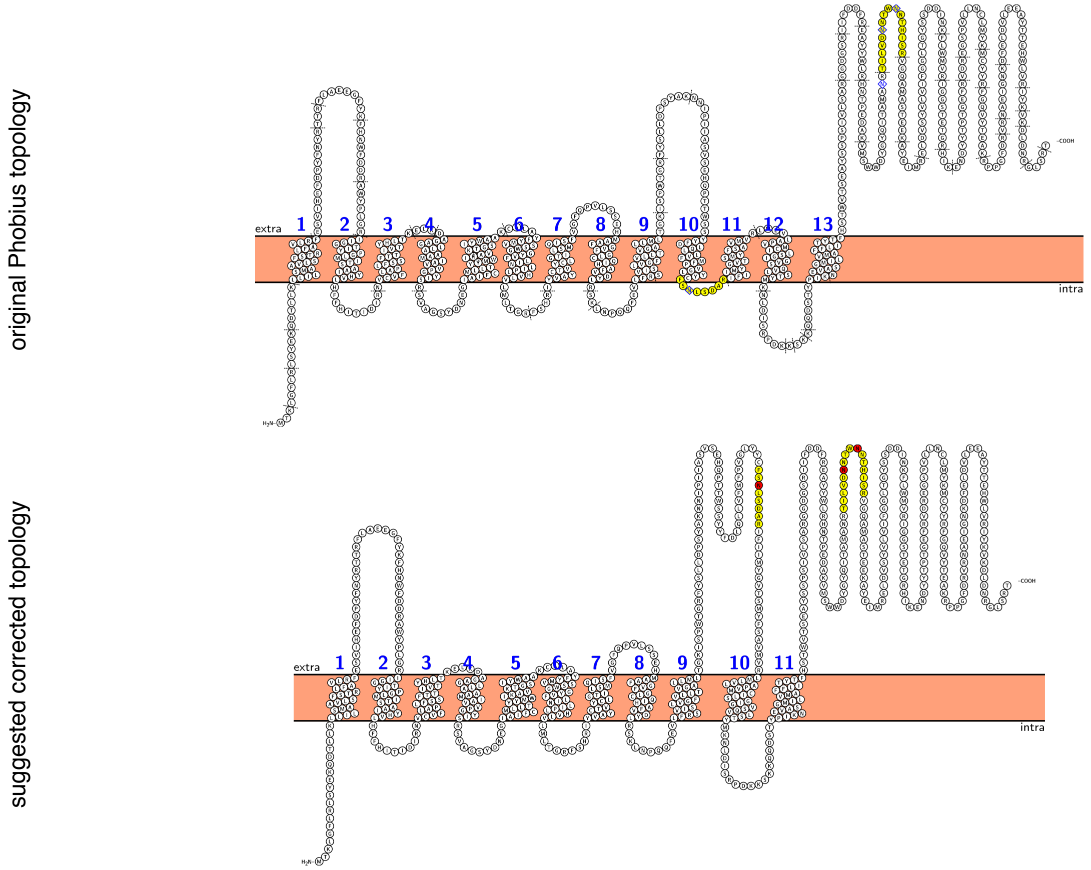

P55088

original Phobius topology

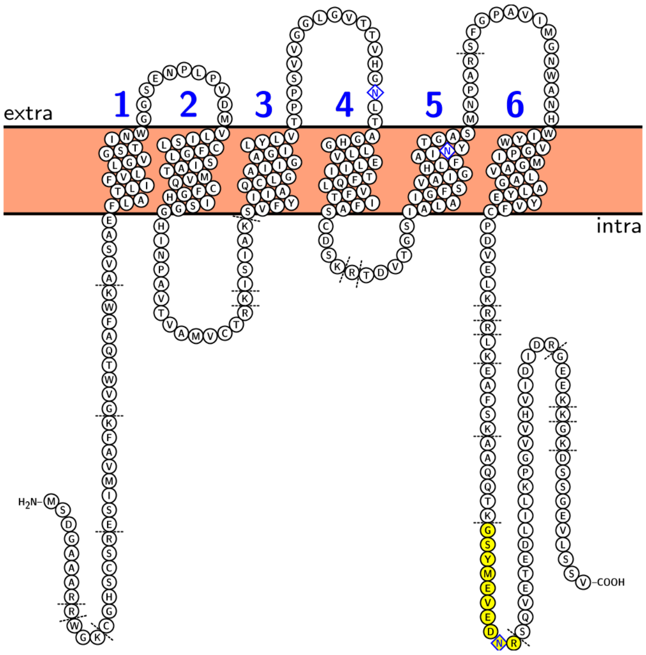

suggested corrected topology

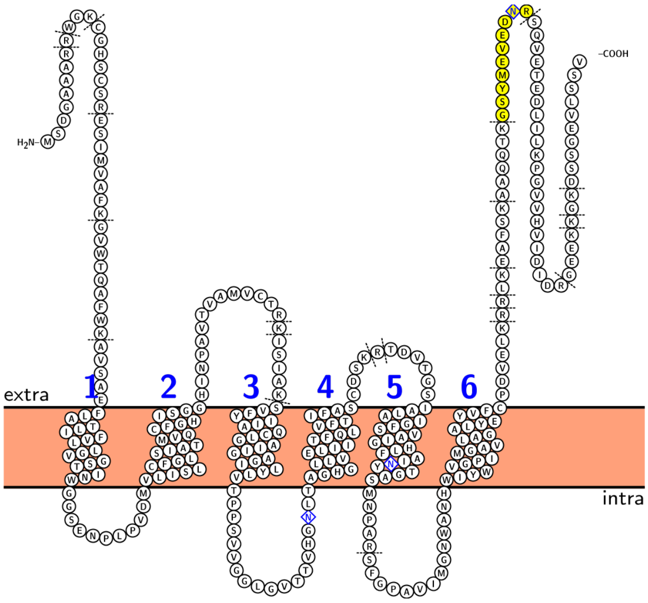

Q01237

original Phobius topology

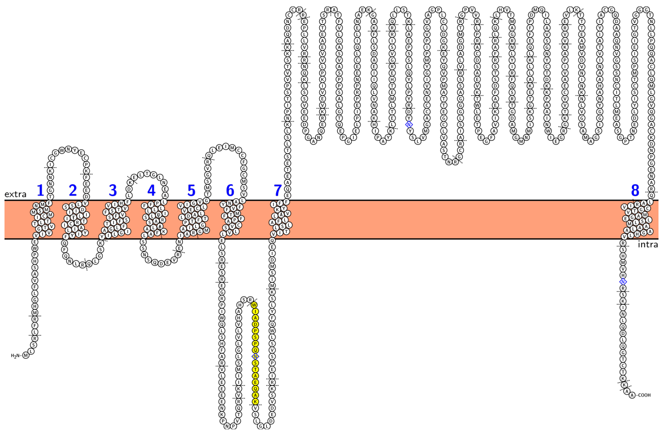

suggested corrected topology

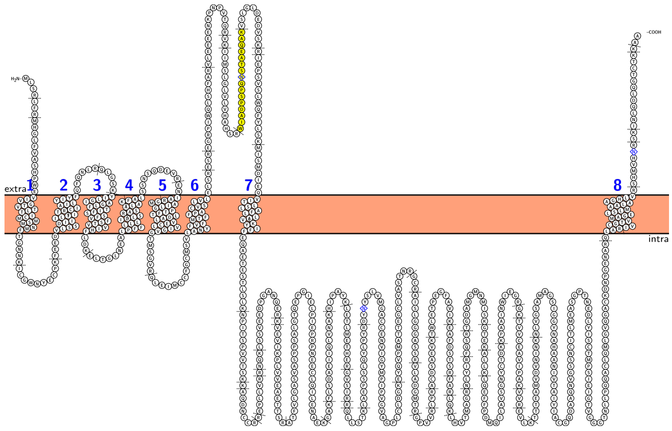

Q01279

original Phobius topology

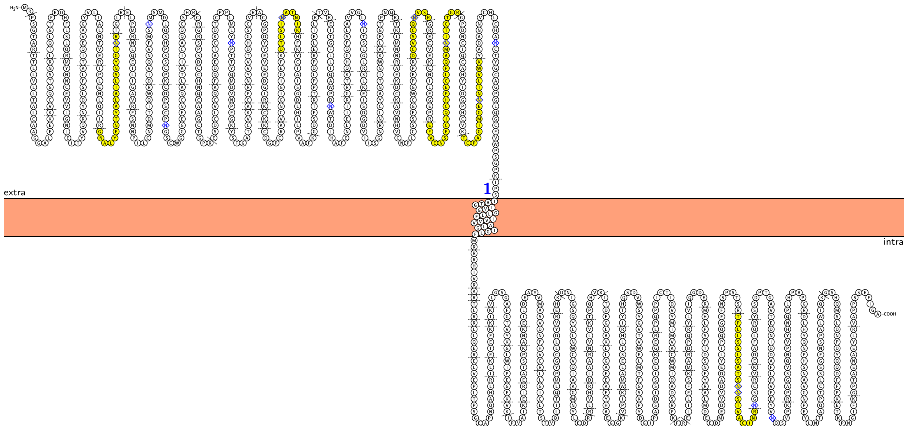

suggested corrected topology

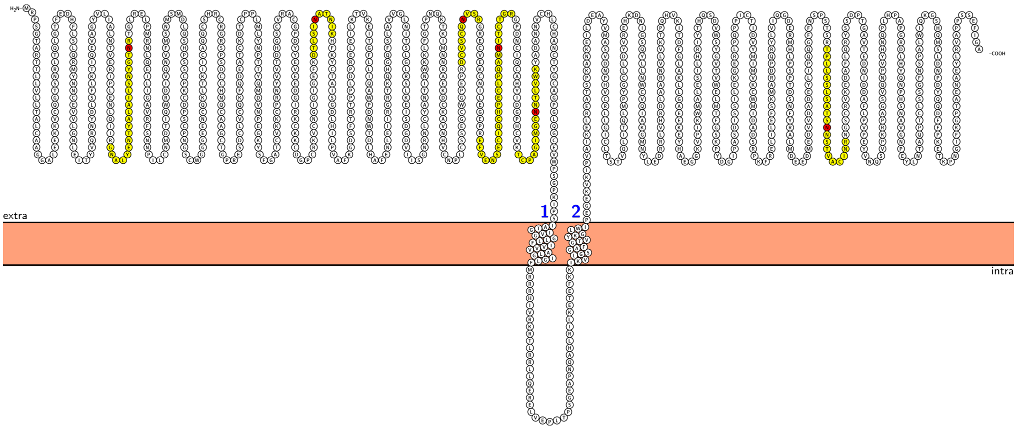

Q3TBN1

original Phobius topology

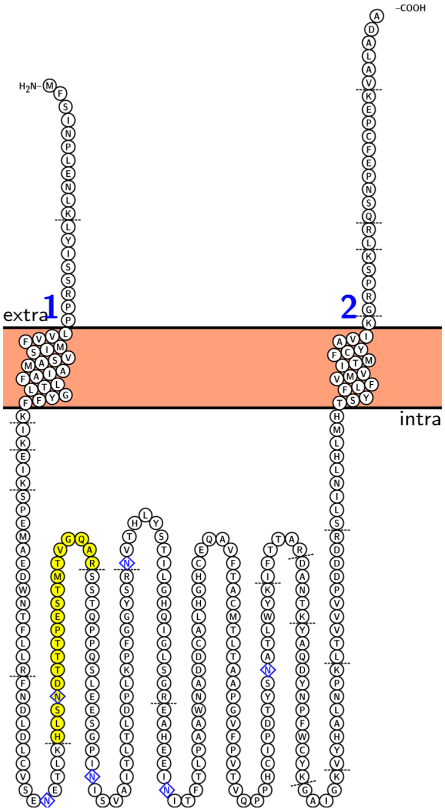

suggested corrected topology

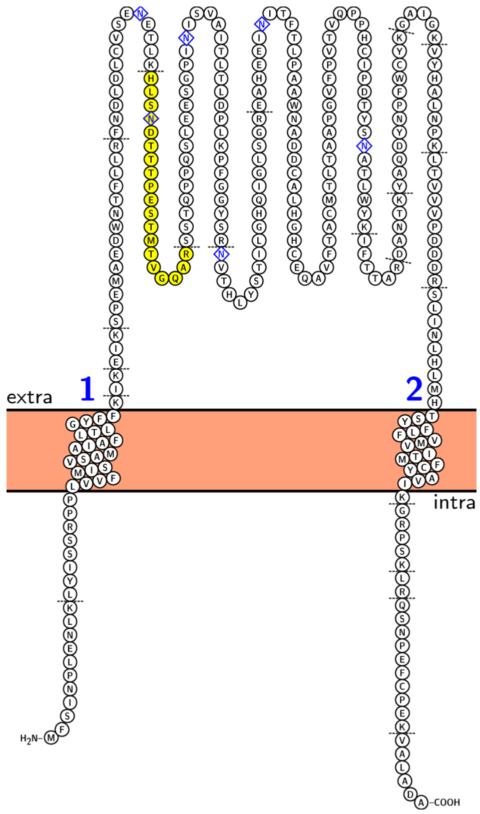

Q3TDN0

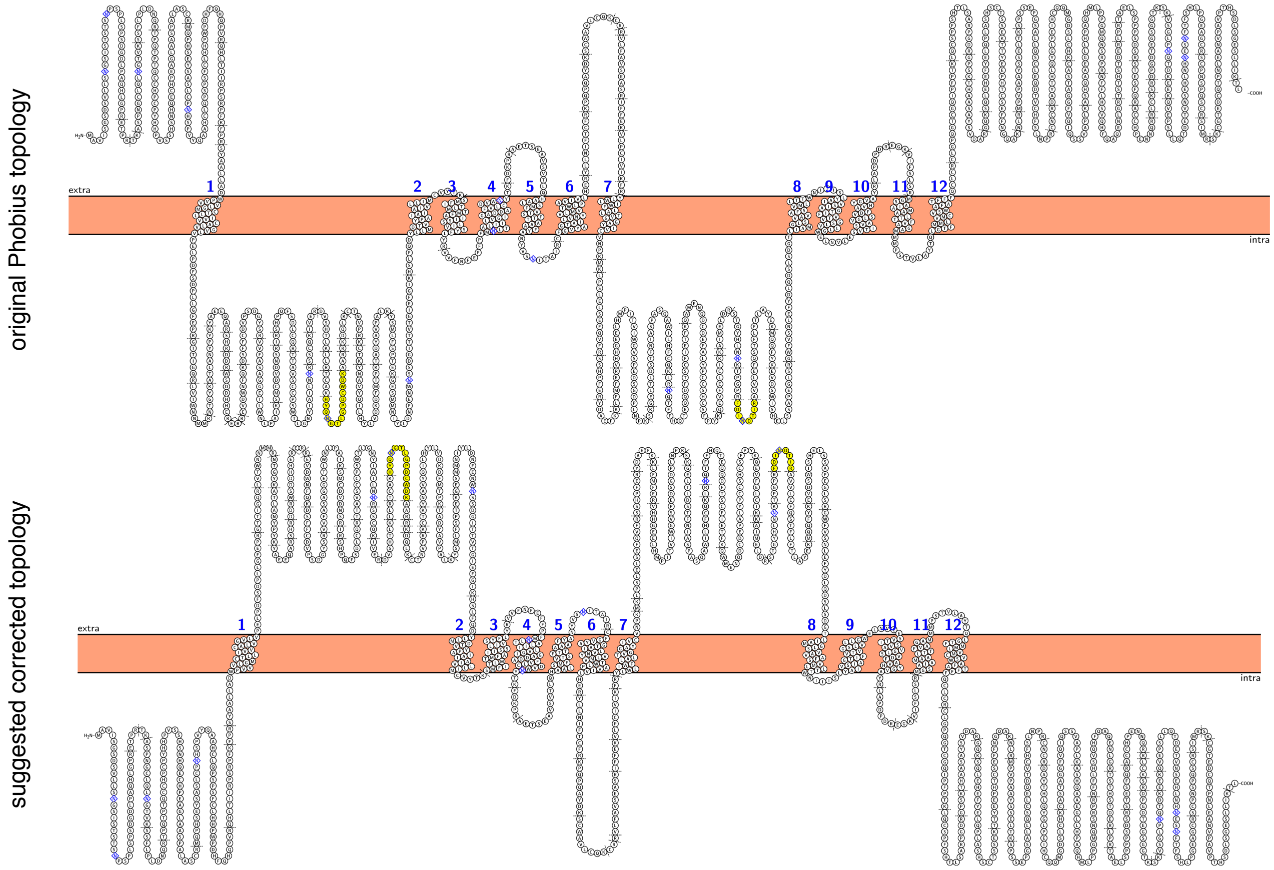

Q3U284

original Phobius topology

suggested corrected topology

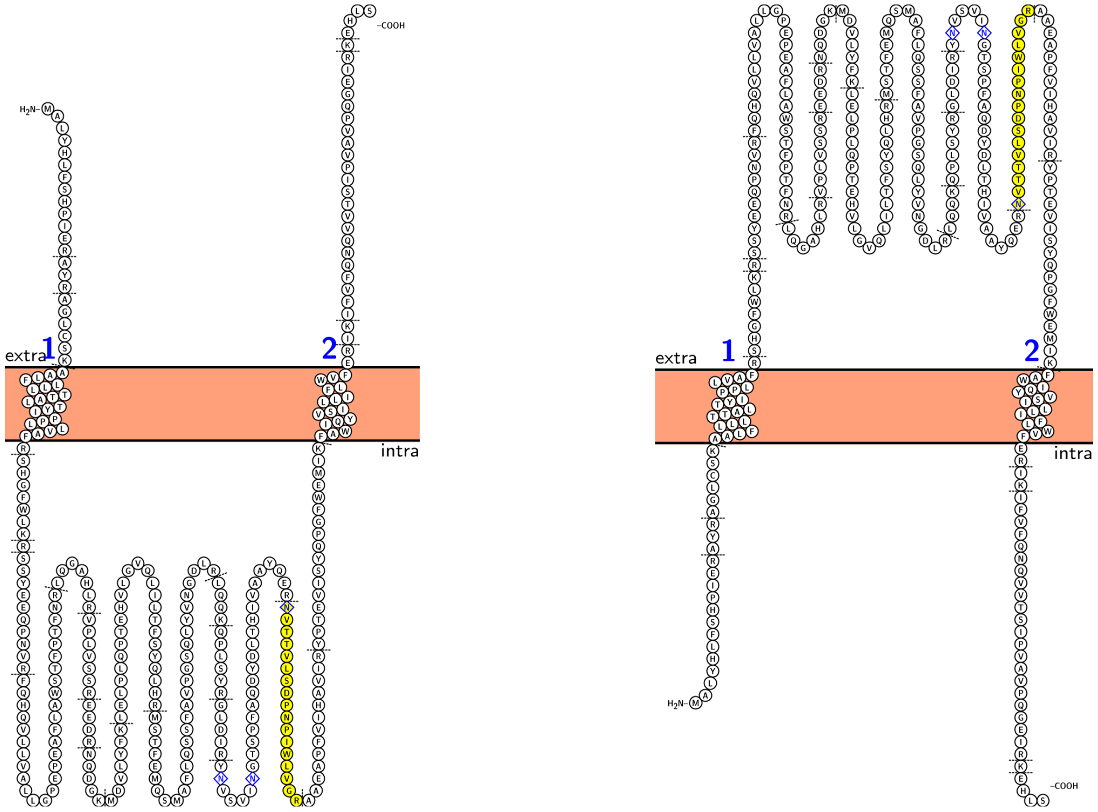

Q61029

original Phobius topology

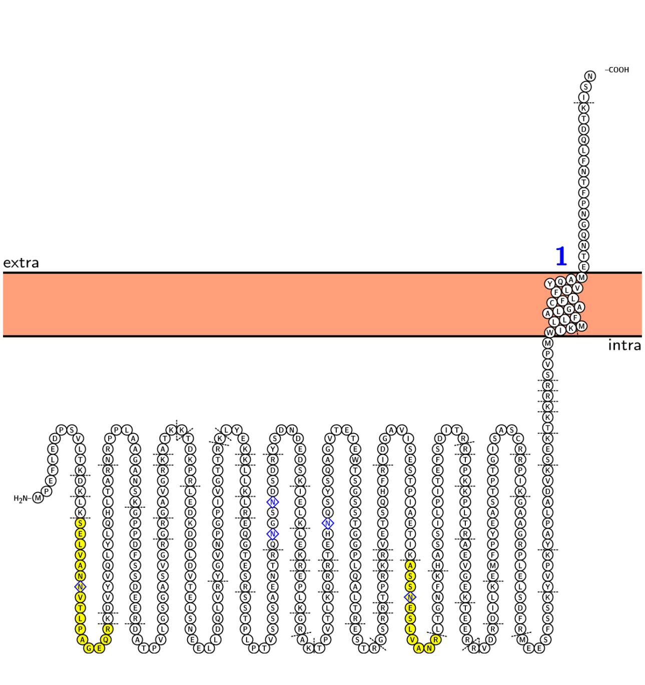

suggested corrected topology

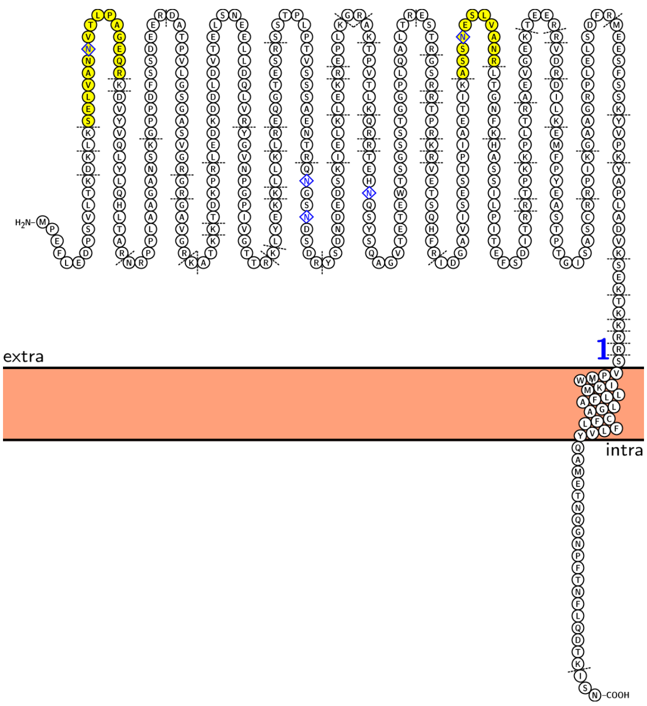

Q68FD9

original Phobius topology

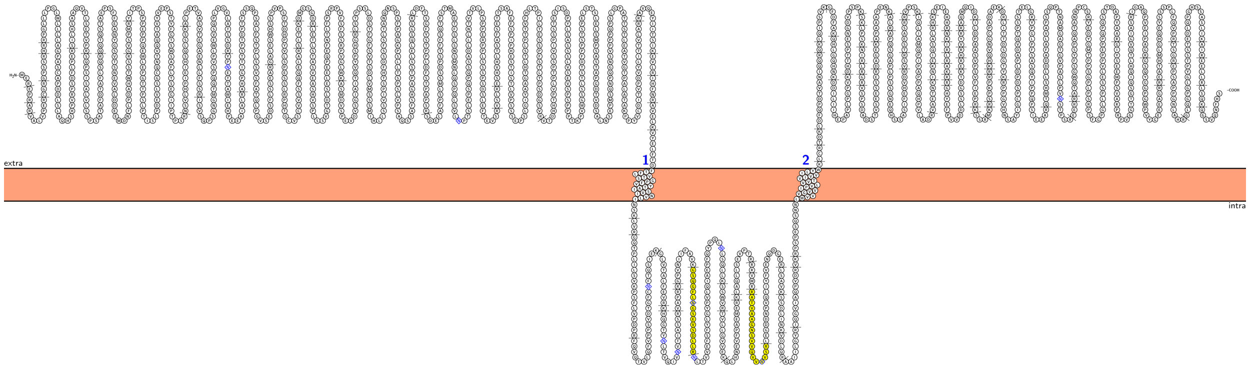

suggested corrected topology

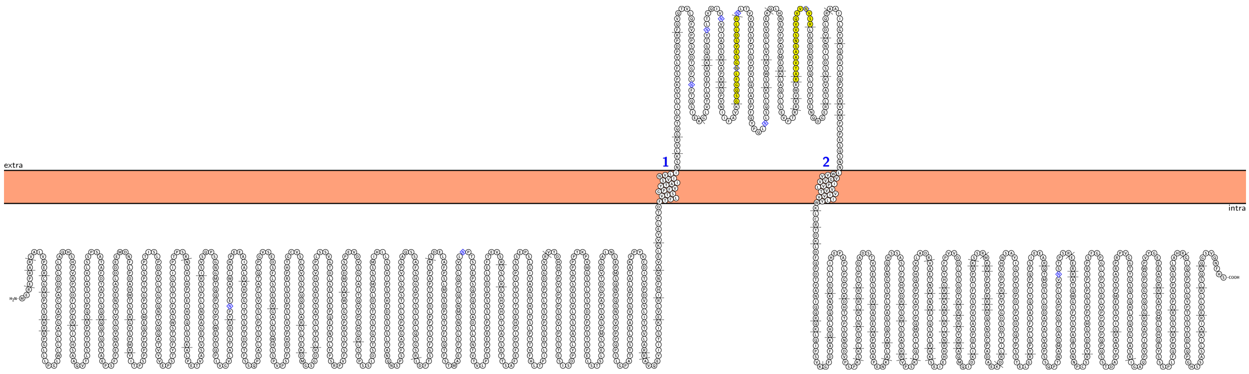

Q68FE2

original Phobius topology

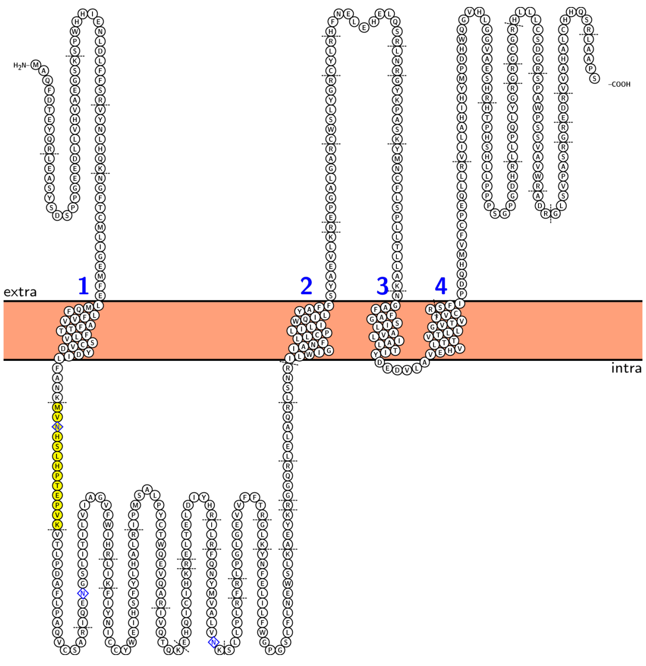

suggested corrected topology

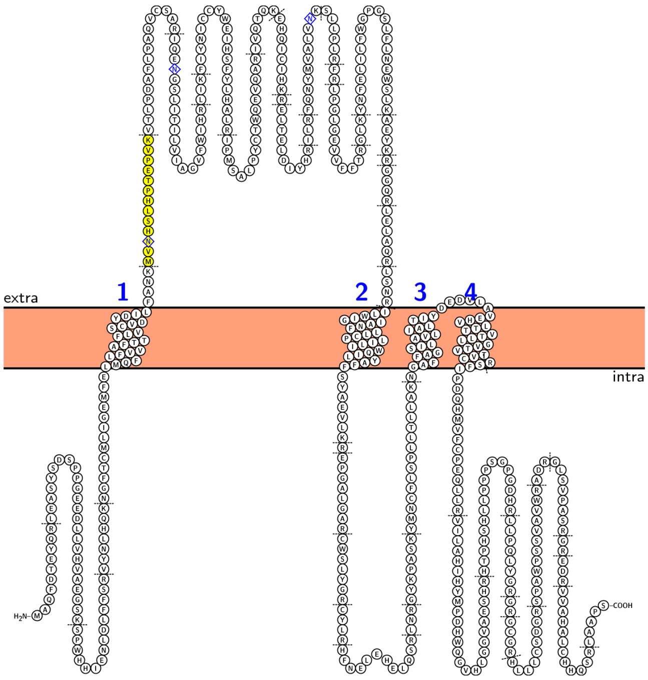

Q6PIC6

original Phobius topology

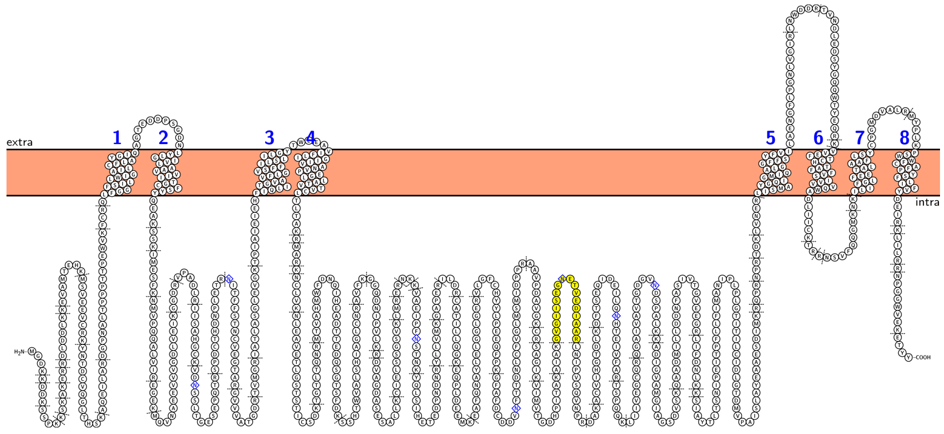

suggested corrected topology

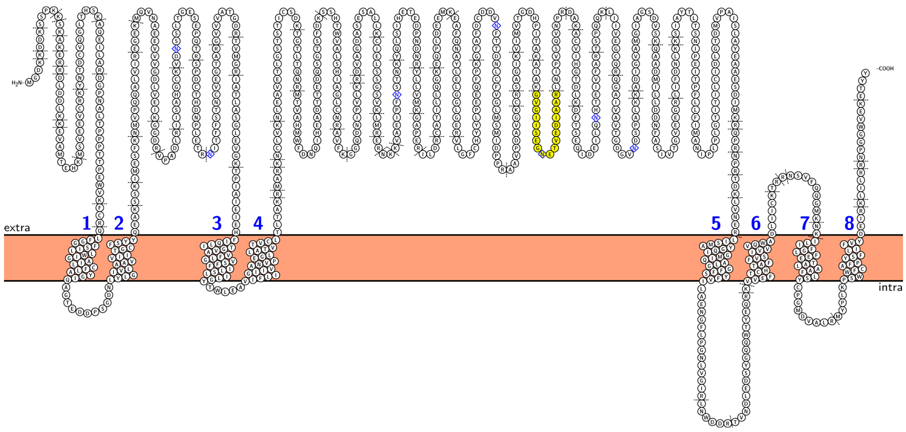

Q6ZQE4

original Phobius topology

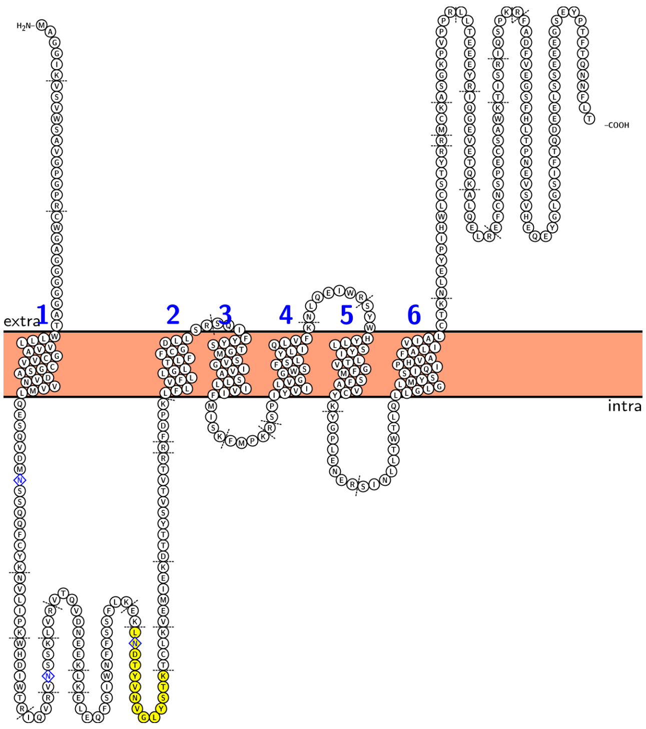

suggested corrected topology

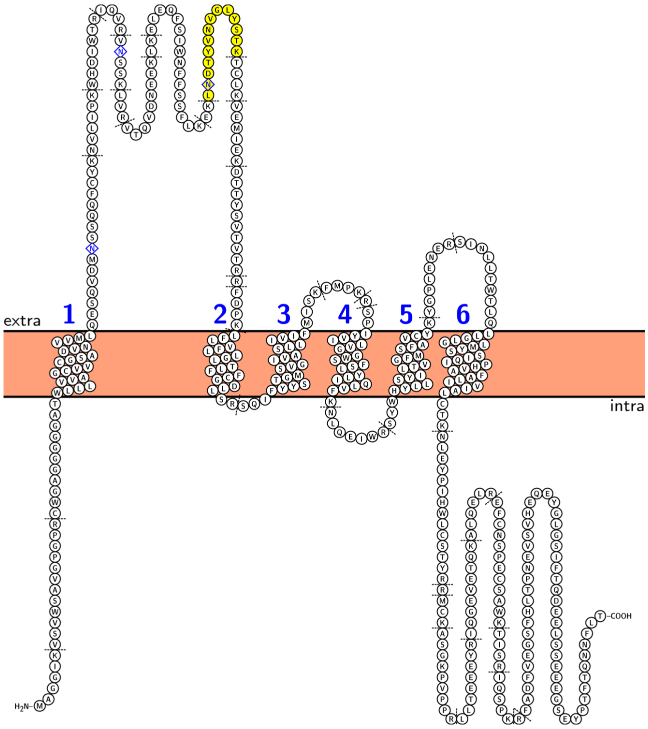

Q8BG18

original Phobius topology

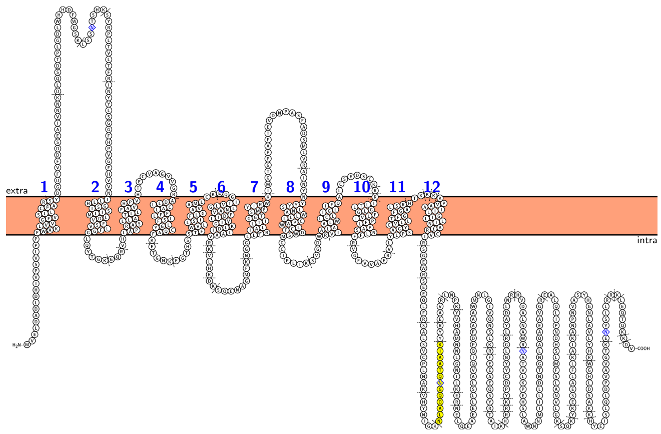

suggested corrected topology

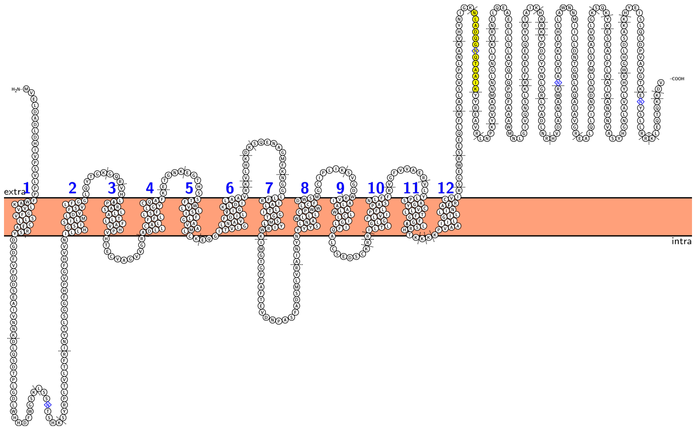

Q8BHG3

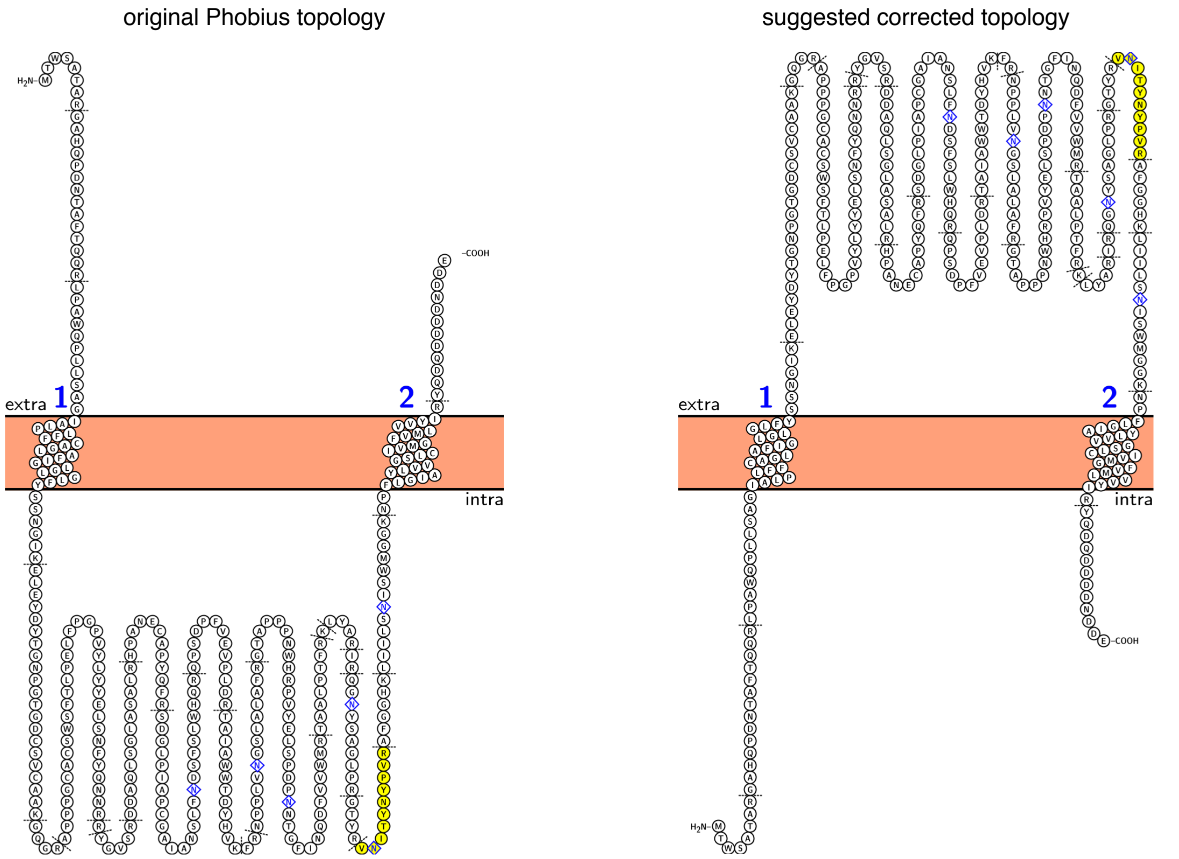

Q8BJ83

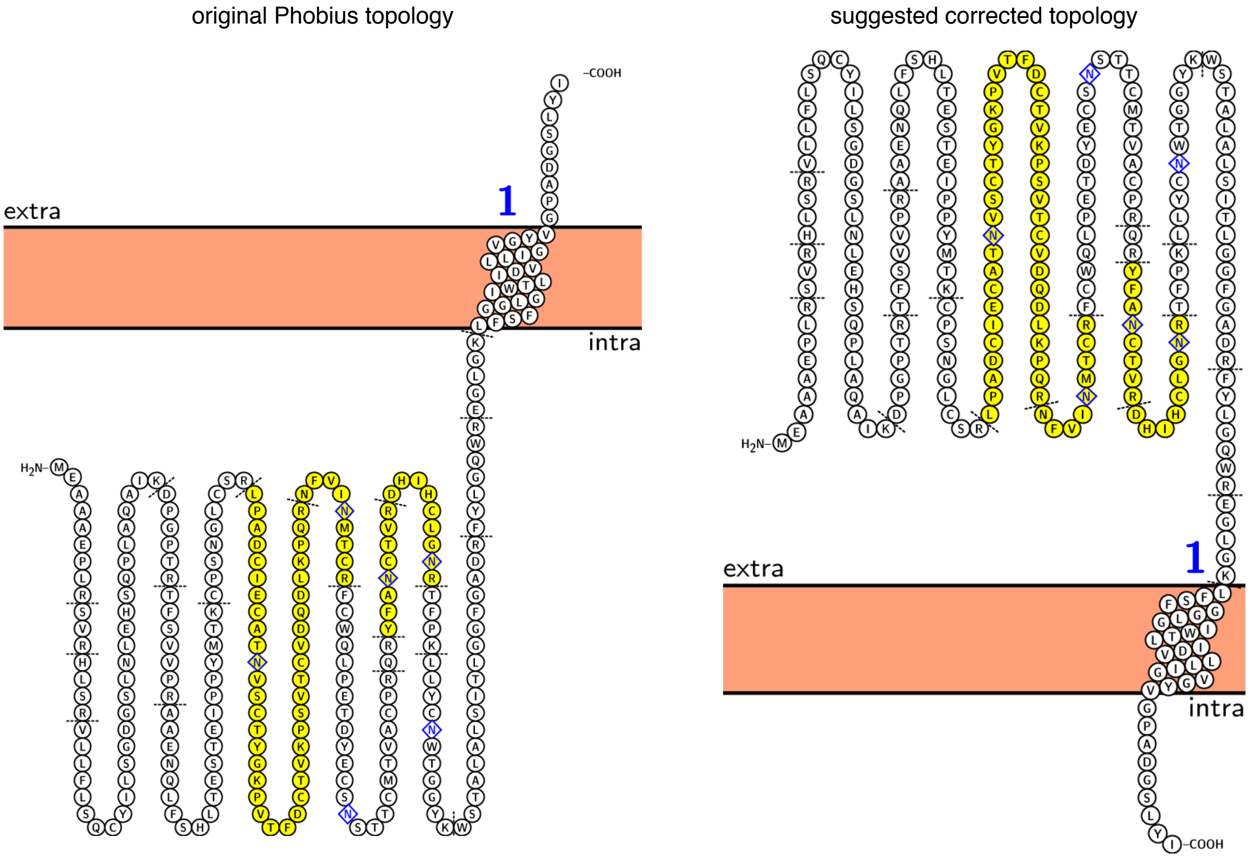

Q8BMK4

original Phobius topology

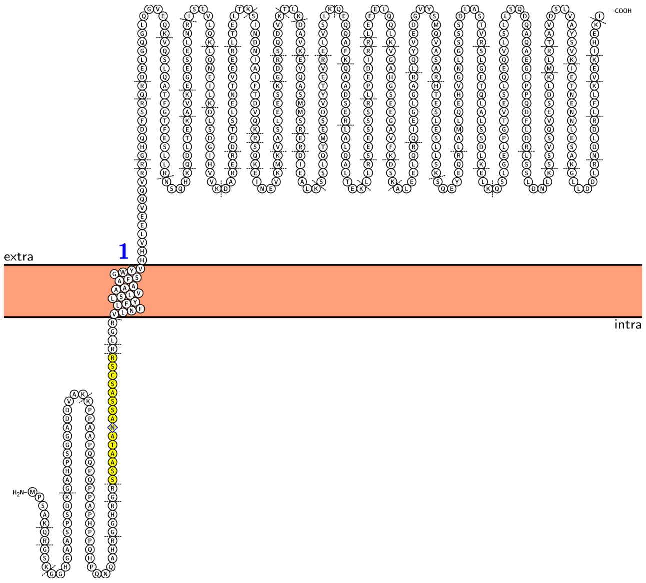

suggested corrected topology

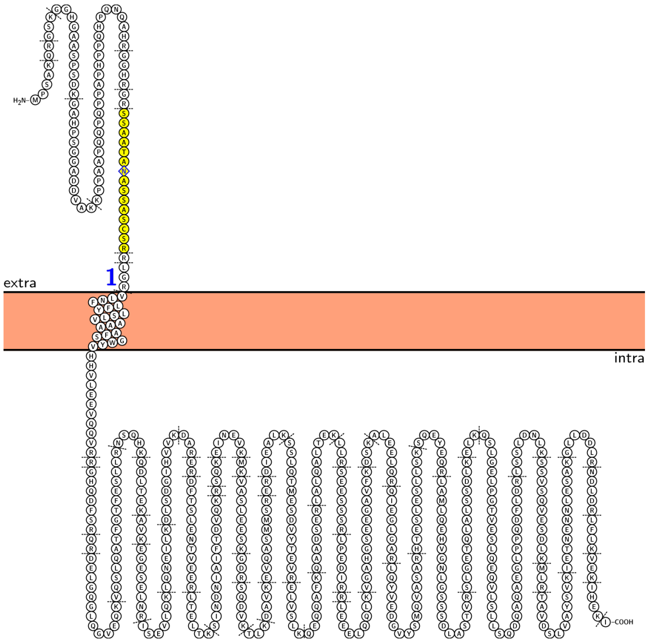

Q8C170

original Phobius topology

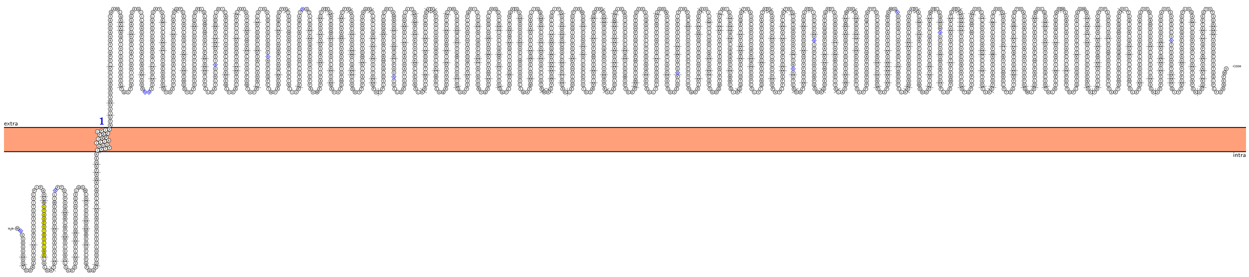

suggested corrected topology

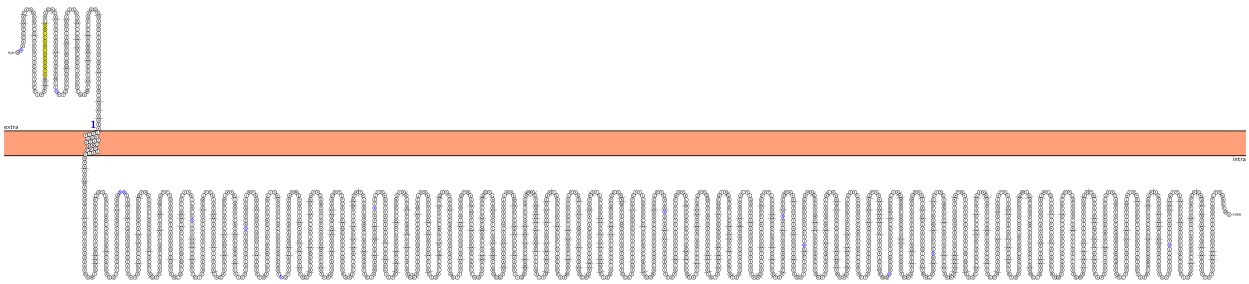

Q8C310

original Phobius topology

suggested corrected topology

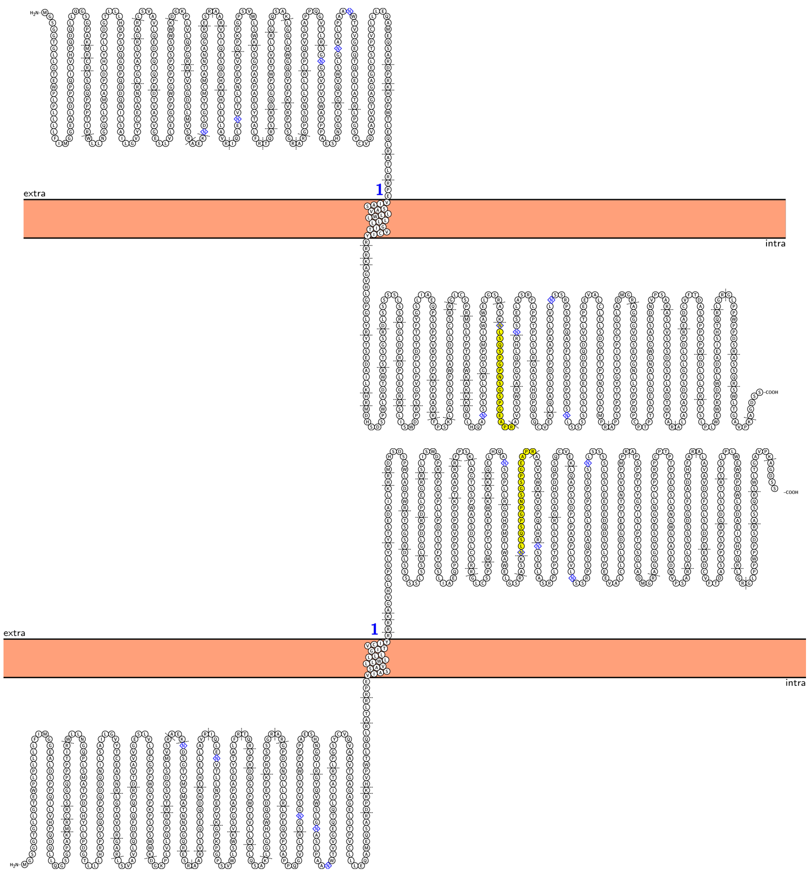

Q8CFE6

original Phobius topology

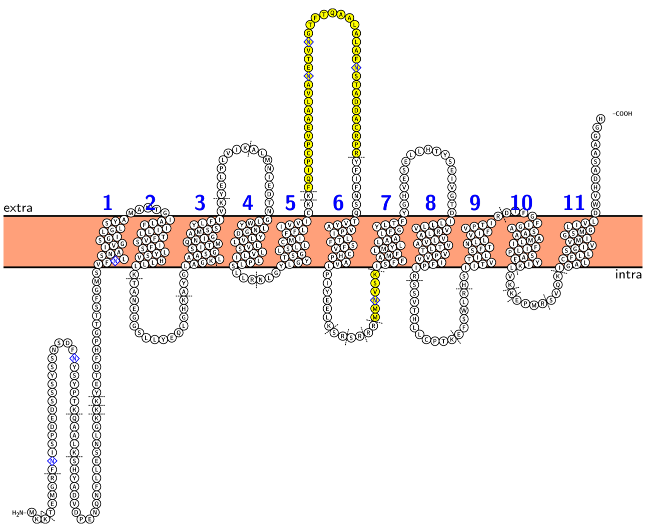

suggested corrected topology

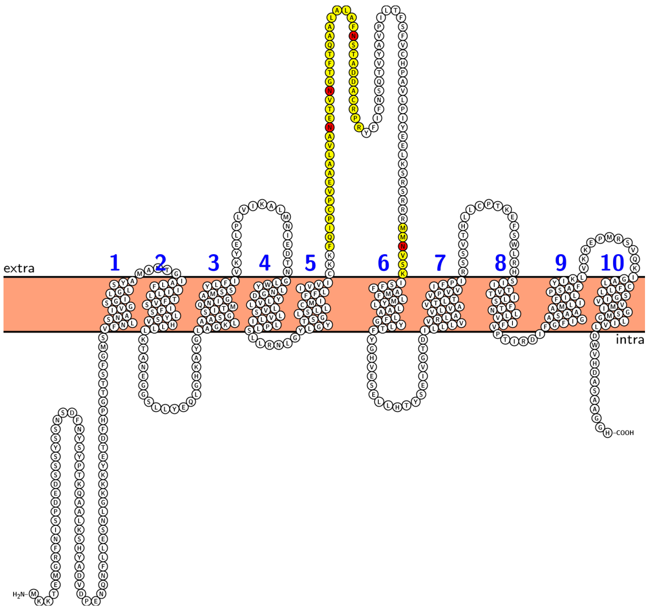

Q8CIV2

original Phobius topology

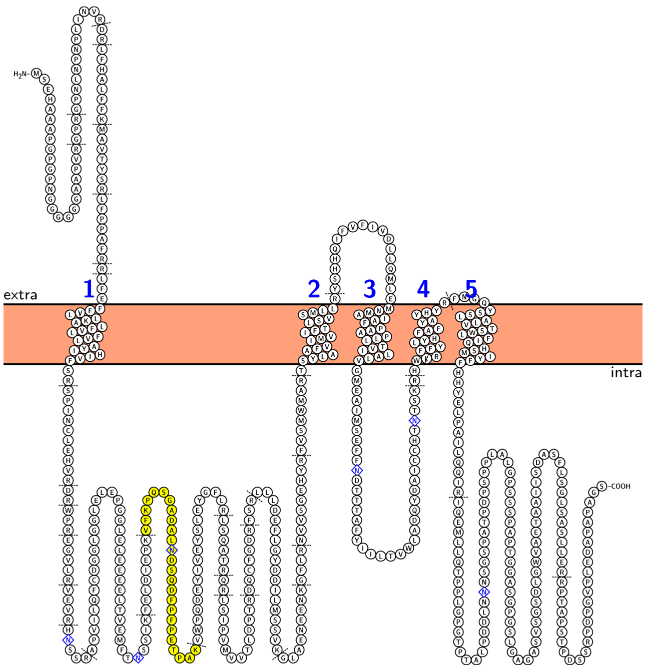

suggested corrected topology

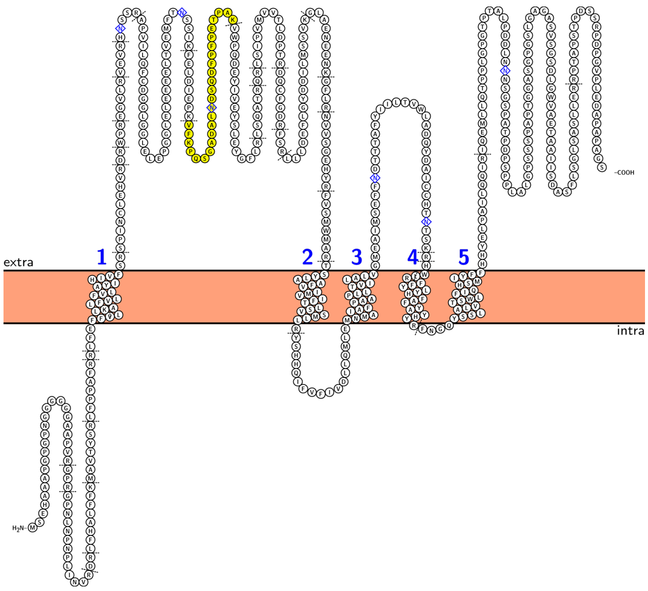

Q8K1G2

original Phobius topology

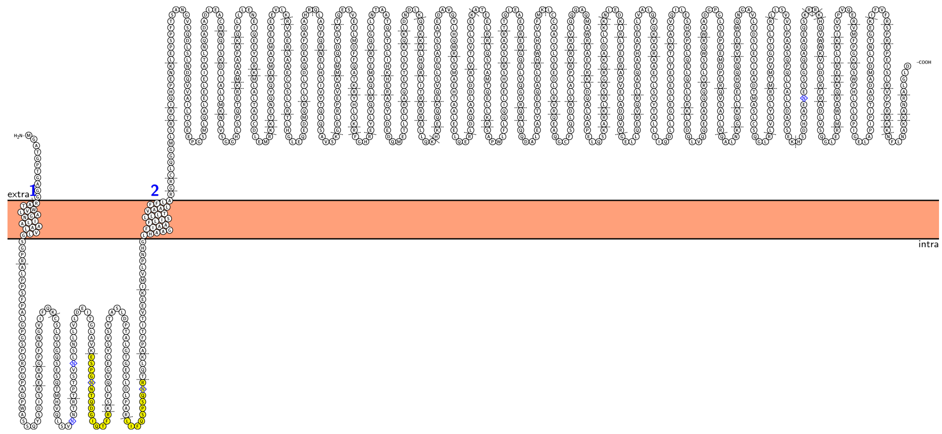

suggested corrected topology

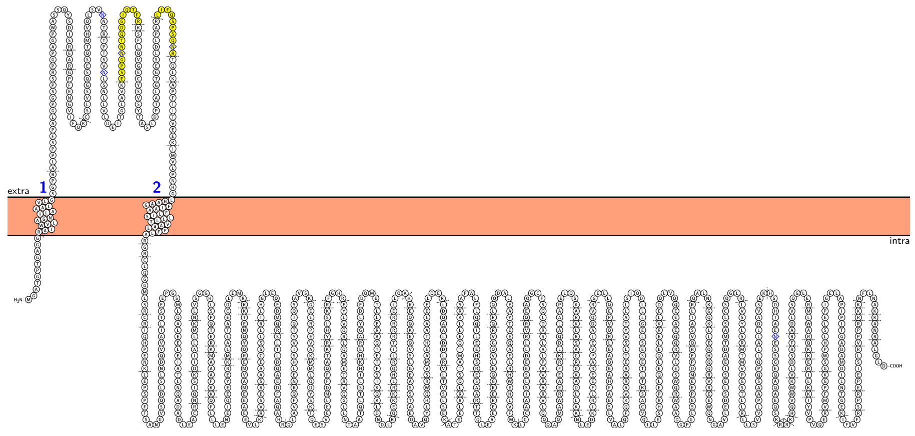

Q8K595

original Phobius topology

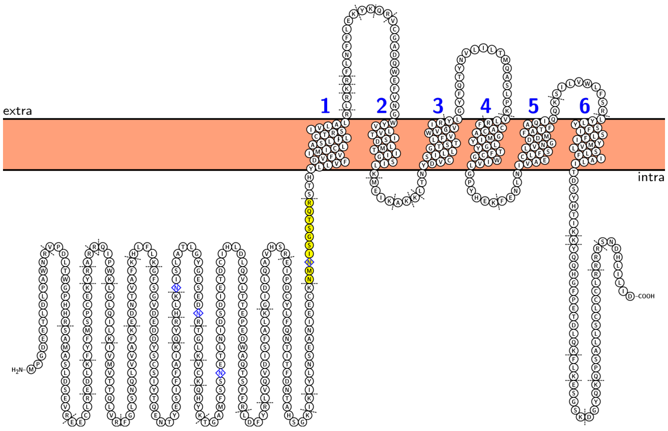

suggested corrected topology

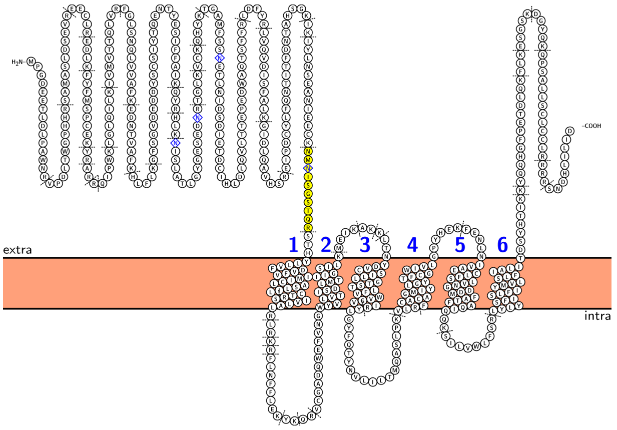

Q8R502

original Phobius topology

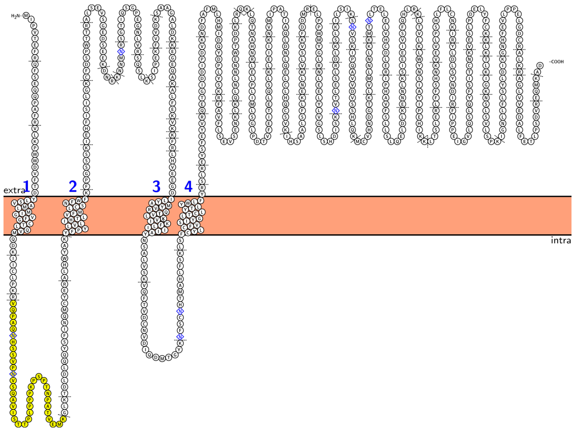

suggested corrected topology

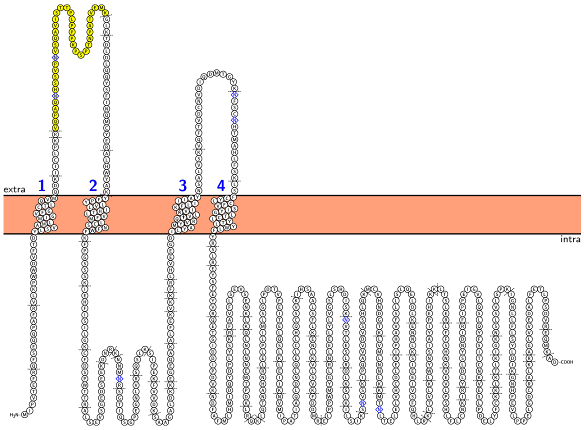

Q8VDN2

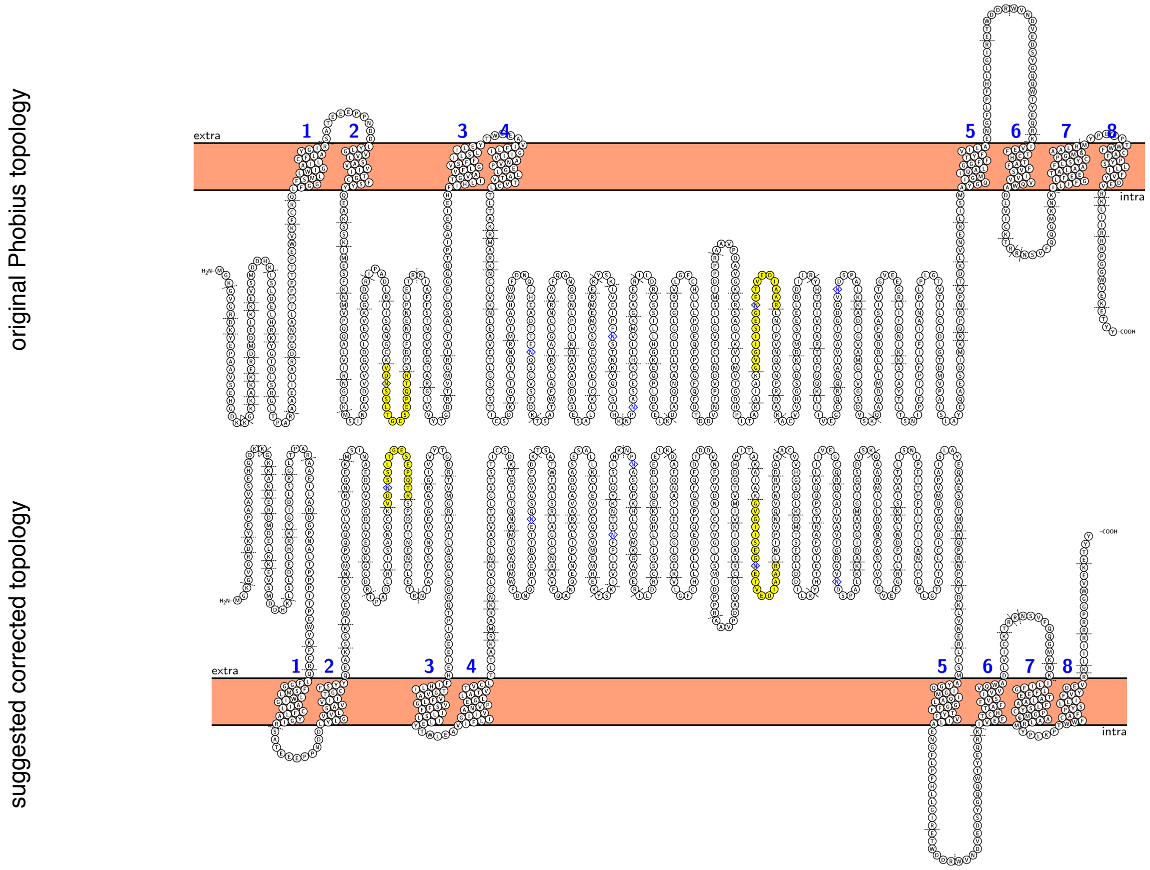

Q91V14

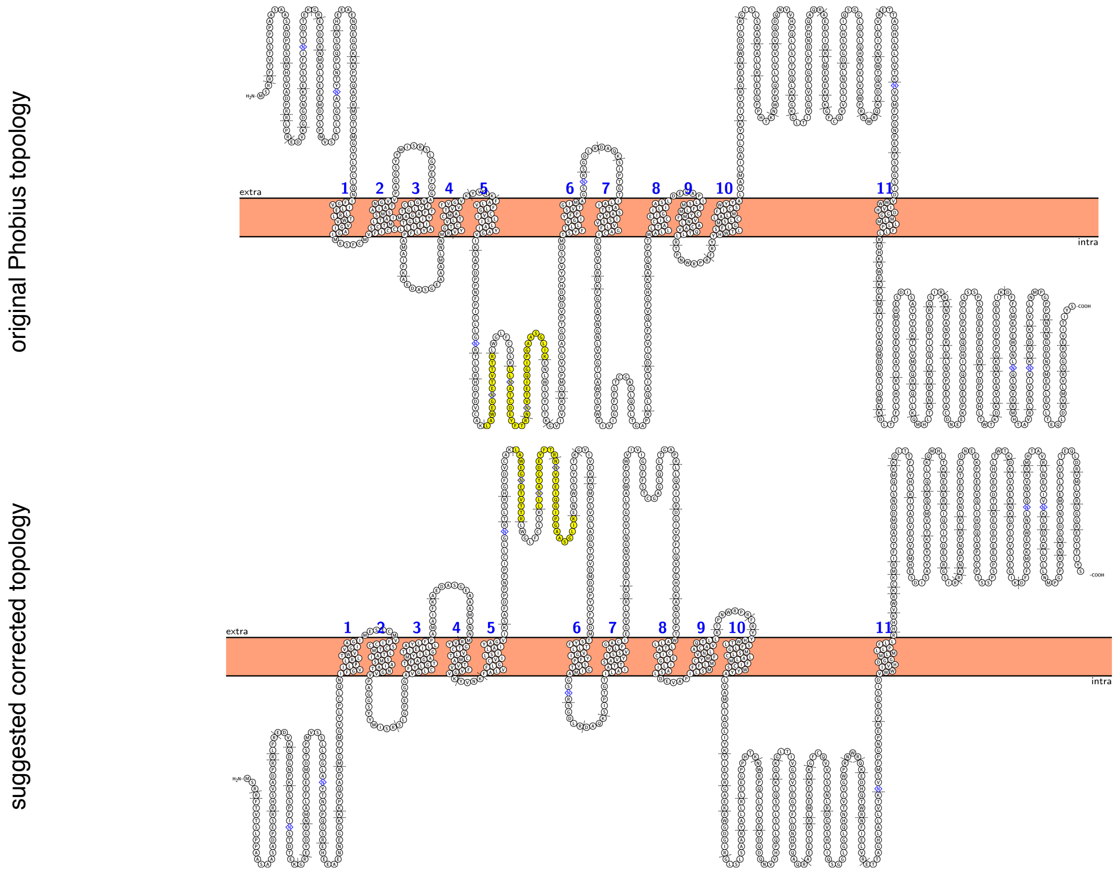

Q91W98

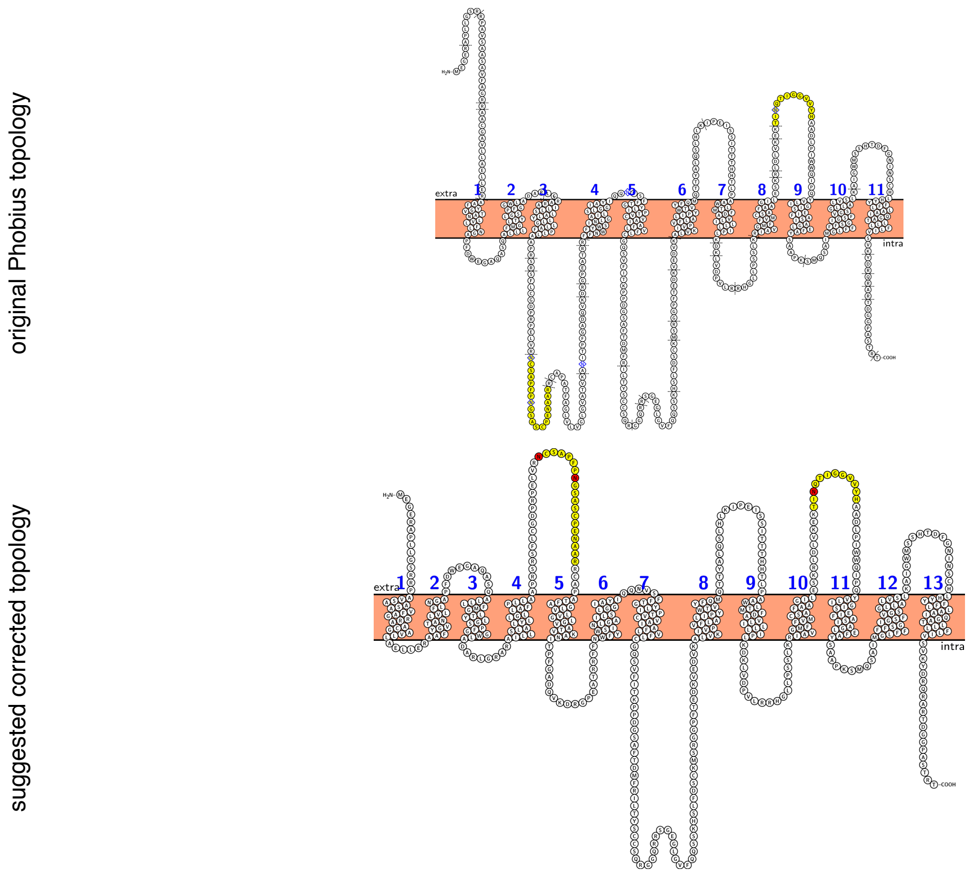

Q924N4

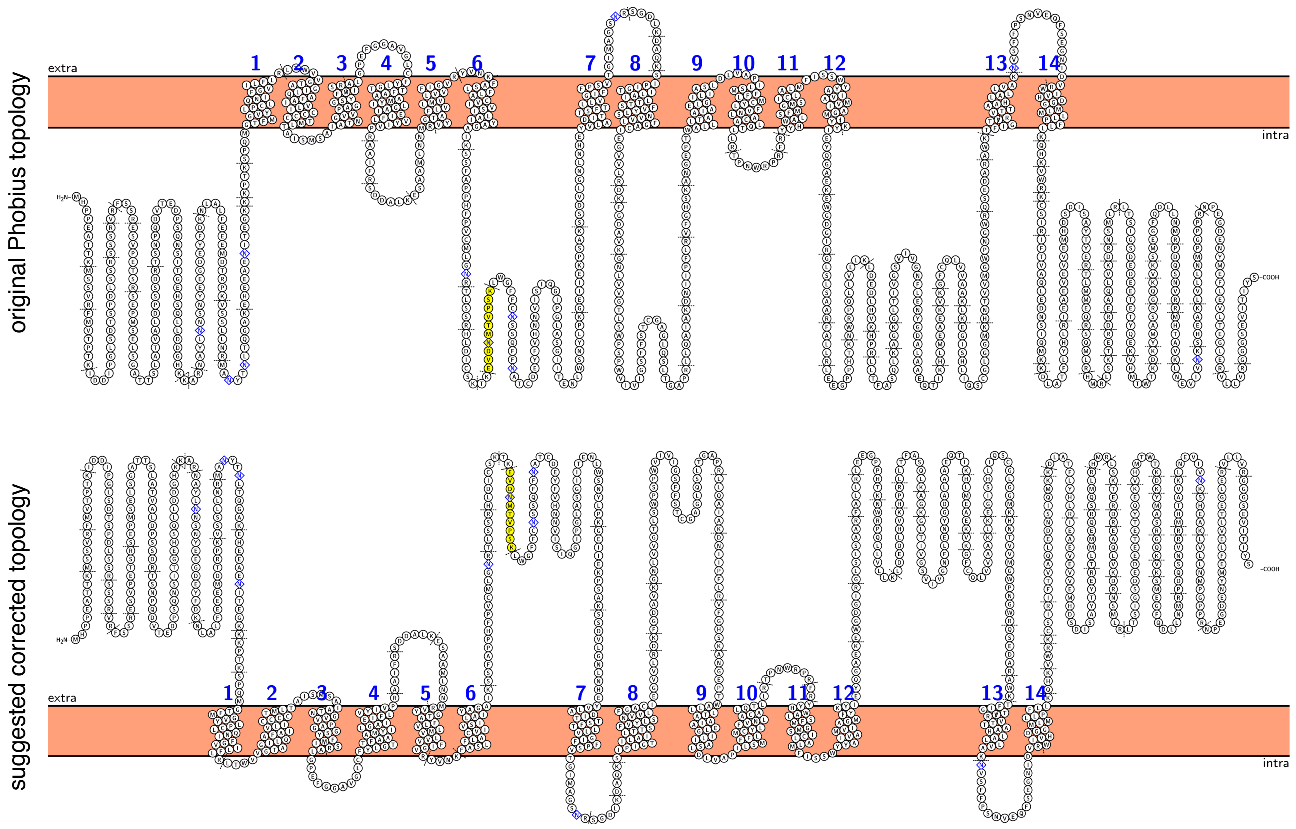

Q99MB3

original Phobius topology

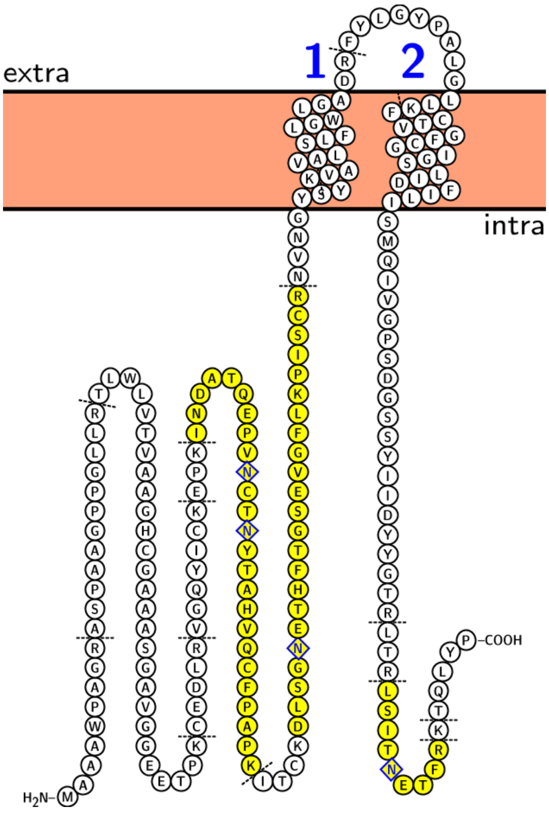

suggested corrected topology

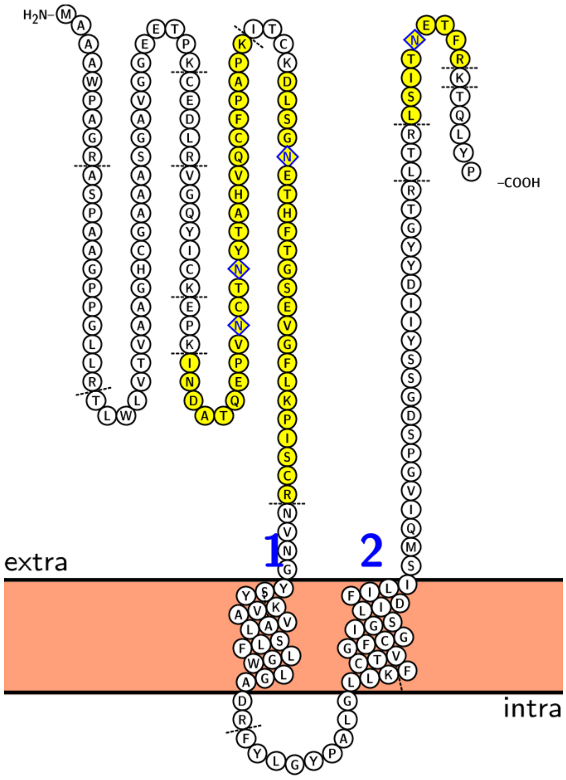

Q9CQX5

original Phobius topology

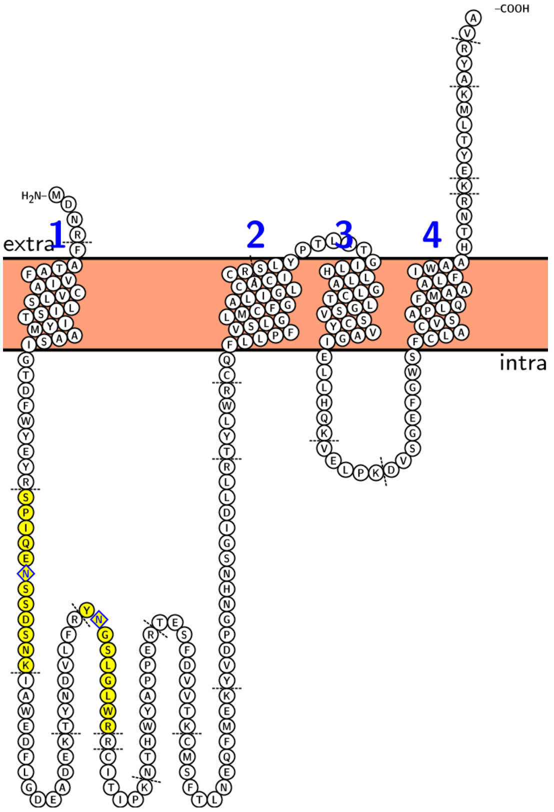

suggested corrected topology

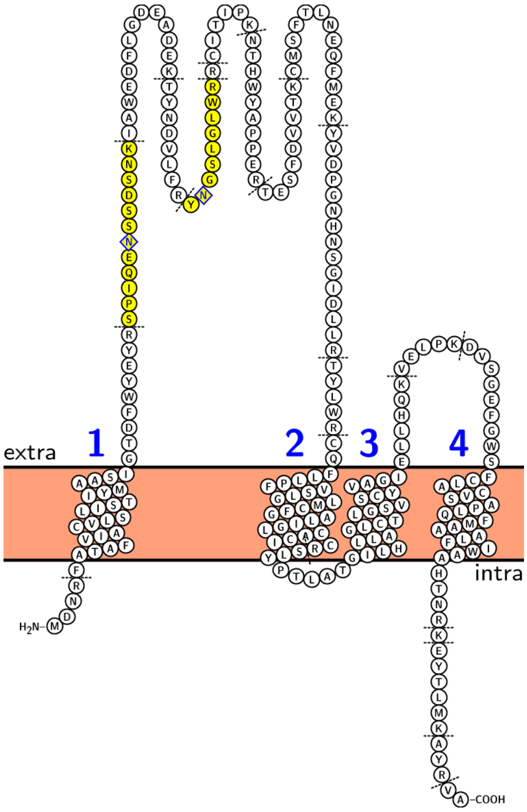

Q9DA75

original Phobius topology

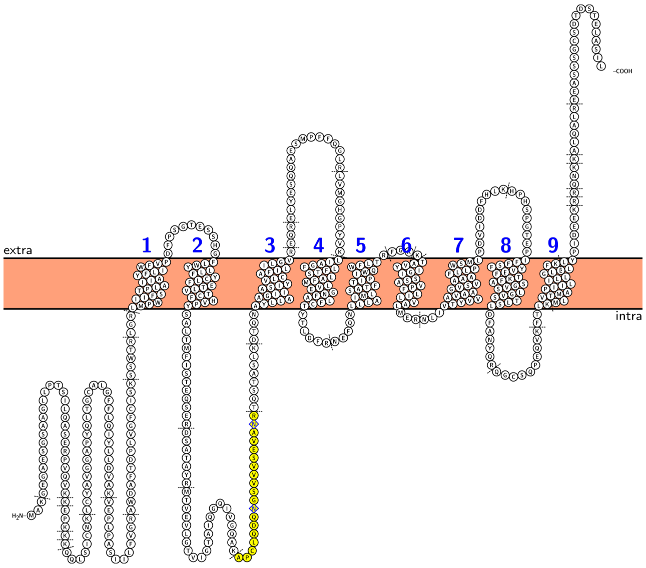

suggested corrected topology

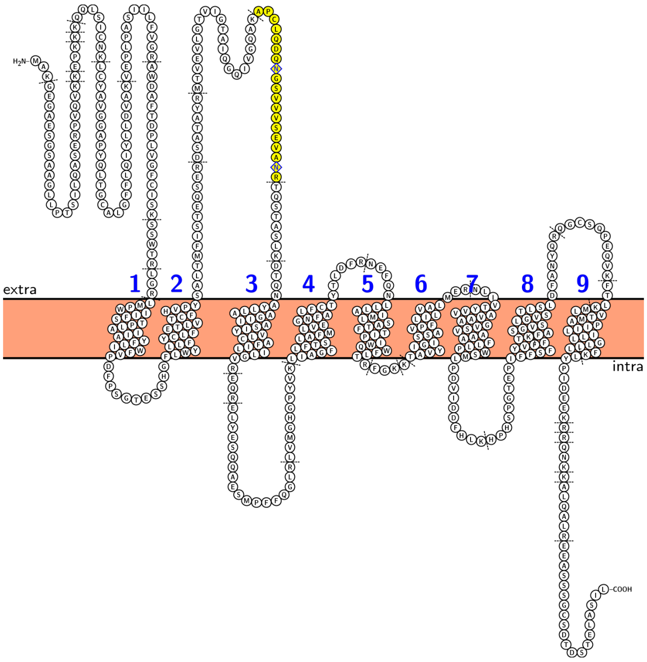

Q9JIP7

original Phobius topology

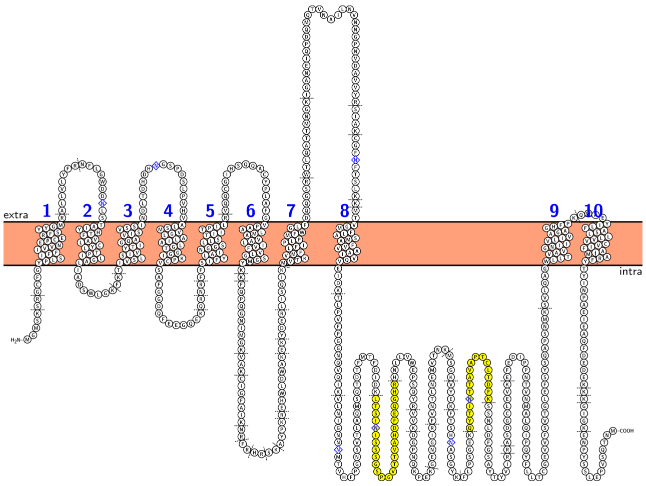

suggested corrected topology

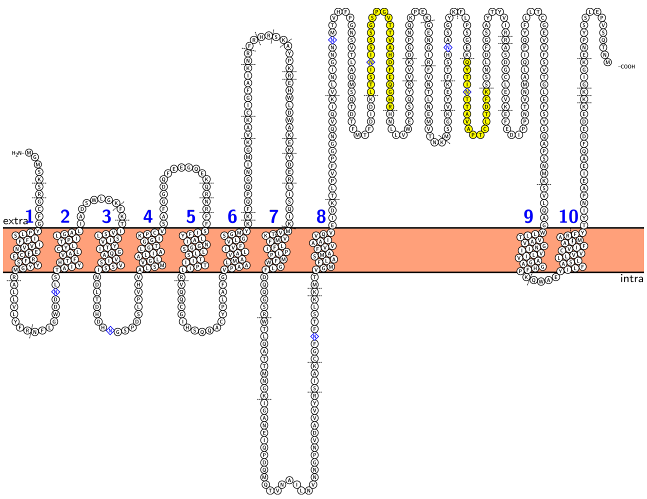

Q9JJI6

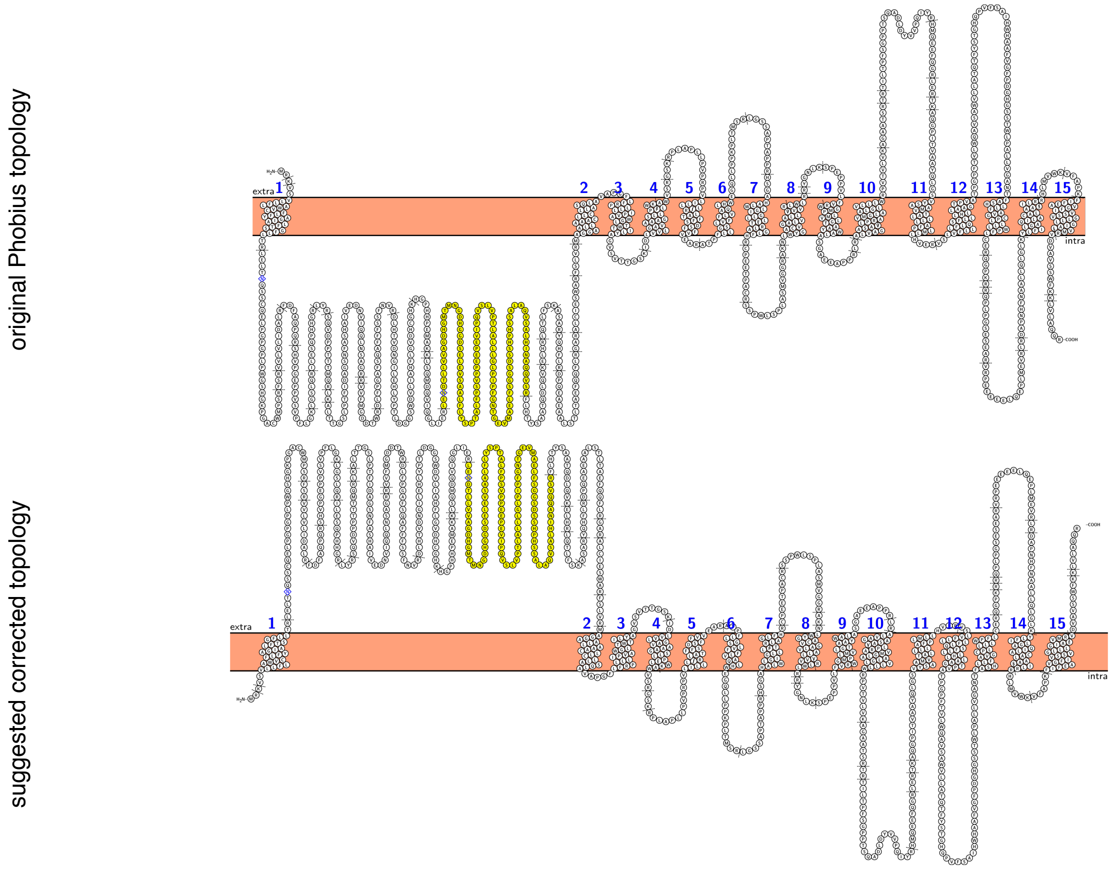

Q9JKZ2

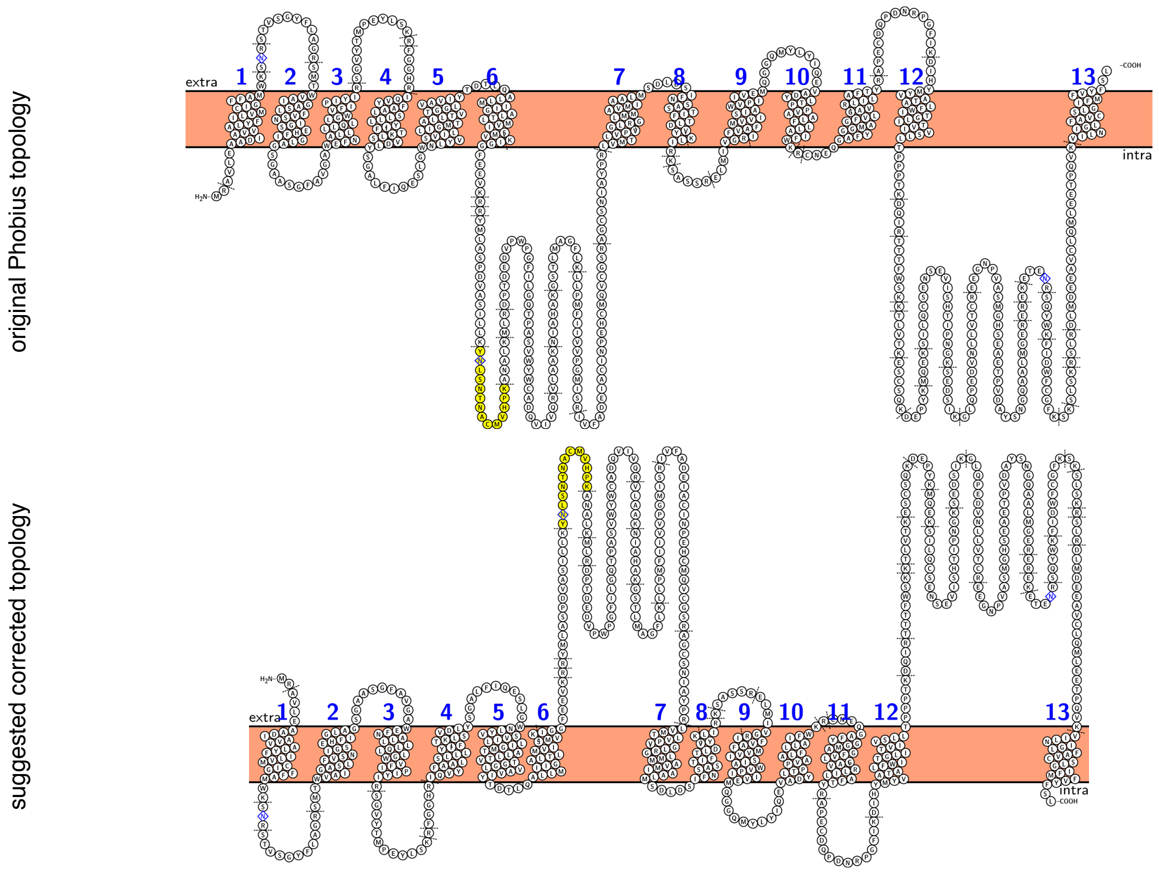

Q9JL99

original Phobius topology

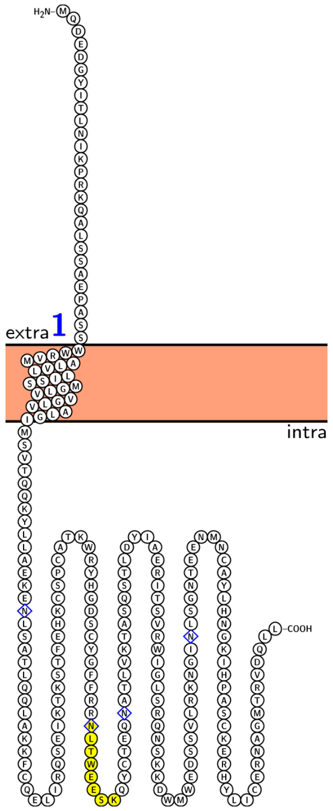

suggested corrected topology

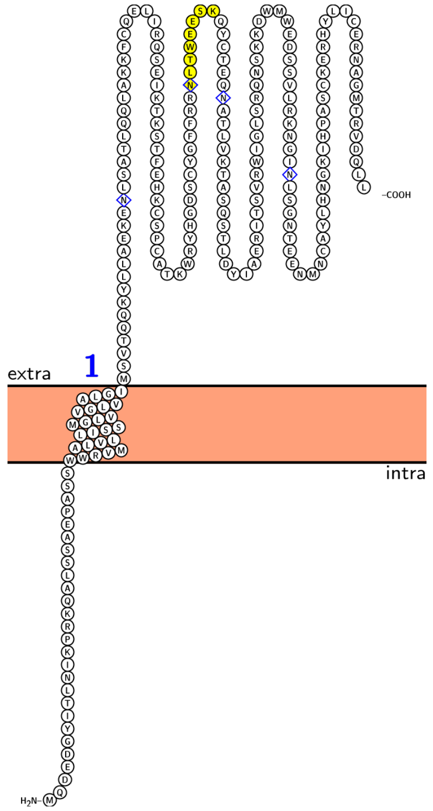

Q9WUB7

original Phobius topology

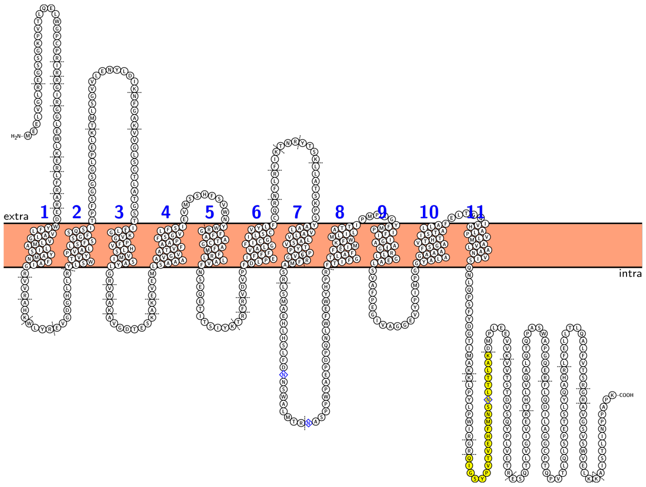

suggested corrected topology

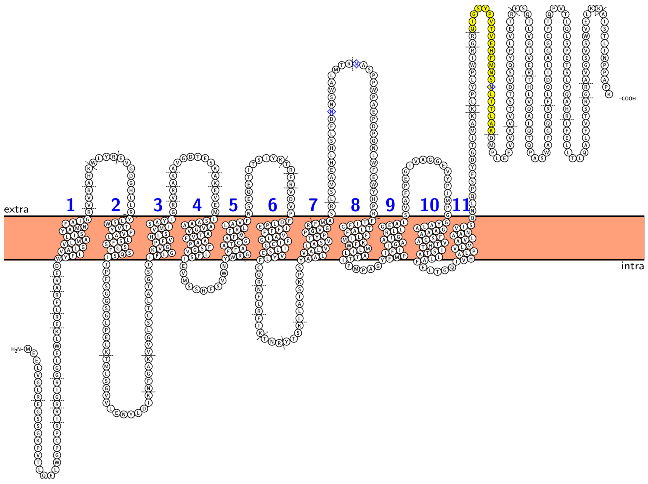

Q9WVD4

original Phobius topology

suggested corrected topology

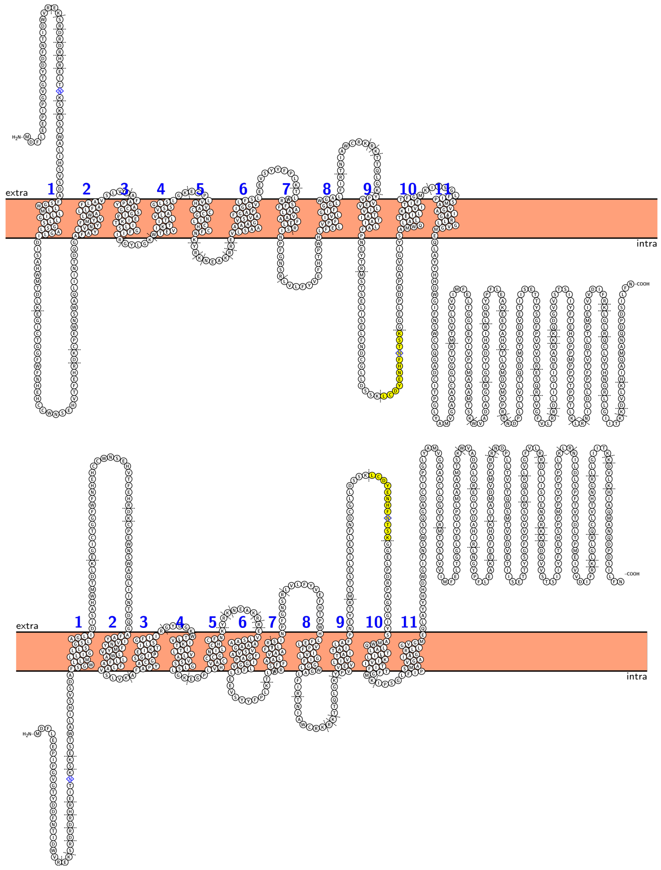

Q9WVL3

original Phobius topology

suggested corrected topology

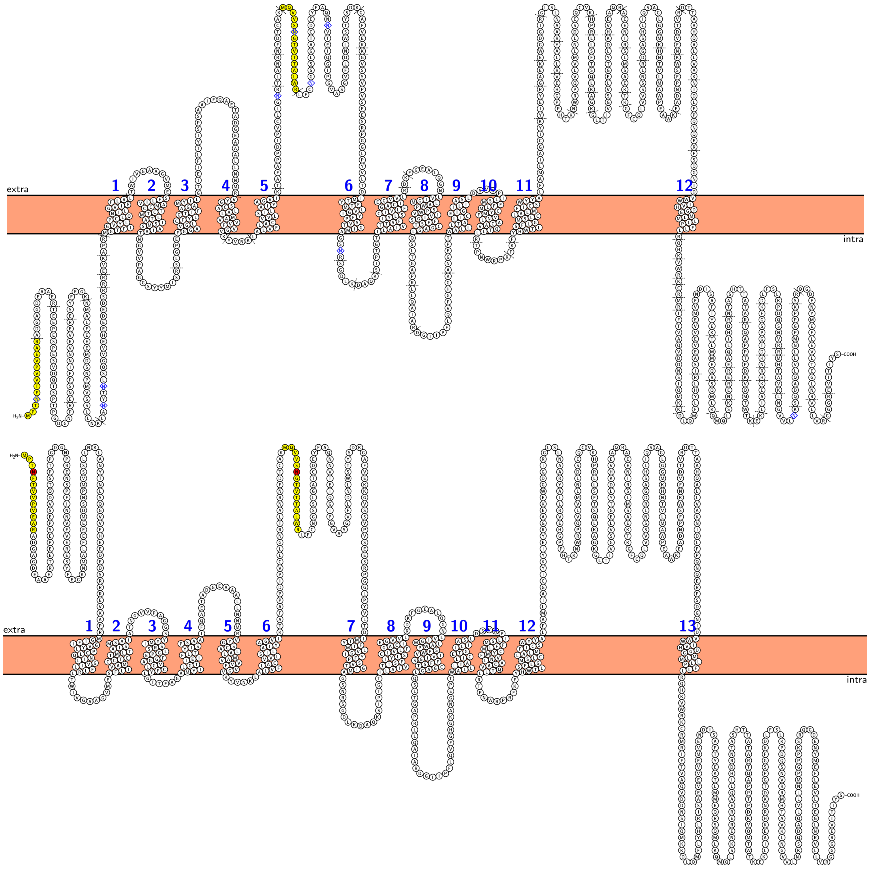

Supplement: S3 File — PDF files with original and based on N-glycopeptide identification corrected topology pictures of 51 human proteins and 39 mouse proteins. The pictures were created with PROTTER and identified N-glycopeptides were marked yellow. (PDF) [file pone.0121314.s004.pdf]
